# Mapping side chain interactions at protein helix termini

**DOI:** 10.1186/s12859-015-0671-4

**Published:** 2015-07-25

**Authors:** Nicholas E Newell

**Affiliations:** Independent researcher, Reading, MA USA

**Keywords:** Helix caps, Helix capping, Helix terminus, Helix termini, Helix-terminal loop, Data mining, Statistical feature detection, Statistical motif detection, Asx N-cap, ST N-cap, Capping box, Big box, CxxC motif

## Abstract

**Background:**

Interactions that involve one or more amino acid side chains near the ends of protein helices stabilize helix termini and shape the geometry of the adjacent loops, making a substantial contribution to overall protein structure. Previous work has identified key helix-terminal motifs, such as Asx/ST N-caps, the capping box, and hydrophobic and electrostatic interactions, but important questions remain, including: 1) What loop backbone geometries are favoured by each motif? 2) To what extent are multi-amino acid motifs likely to represent genuine cooperative interactions? 3) Can new motifs be identified in a large, recent dataset using the latest bioinformatics tools?

**Results:**

Three analytical tools are applied here to answer these questions. First, helix-terminal structures are partitioned by loop backbone geometry using a new 3D clustering algorithm. Next, Cascade Detection, a motif detection algorithm recently published by the author, is applied to each cluster to determine which sequence motifs are overrepresented in each geometry. Finally, the results for each motif are presented in a CapMap, a 3D conformational heatmap that displays the distribution of the motif’s overrepresentation across loop geometries, enabling the rapid isolation and characterization of the associated side chain interaction. This work identifies a library of geometry-specific side chain interactions that provides a new, detailed picture of loop structure near the helix terminus. Highlights include determinations of the favoured loop geometries for the Asx/ST N-cap motifs, capping boxes, “big” boxes, and other hydrophobic, electrostatic, H-bond, and pi stacking interactions, many of which have not been described before.

**Conclusions:**

This work demonstrates that the combination of structural clustering and motif detection in the sequence space can efficiently identify side chain motifs and map them to the loop geometries which they support. Protein designers should find this study useful, because it identifies side chain interactions which are good candidates for inclusion in synthetic helix-terminal loops with specific desired geometries, since they are used in nature to support these geometries. The techniques described here can also be applied to map side chain interactions associated with other structural components of proteins such as beta and gamma turns.

**Electronic supplementary material:**

The online version of this article (doi:10.1186/s12859-015-0671-4) contains supplementary material, which is available to authorized users.

## Background

Alpha helices in proteins are stabilized by *i* → (*i*-4) hydrogen bonds between backbone amide groups and the backbone carbonyl groups behind them in the helix [1], but the first and last 4 residues of a helix cannot participate in these interactions because they lack bonding partners within the helix. To satisfy these unpaired groups, and to prevent the helix from fraying, capping structures are selected at helix termini [2, 3]. At the helical C-terminus, the backbone carbonyls of the helix are often satisfied by hydrogen bonds from main chain amide groups in the loop ahead, forming the Schellman and αL motifs [4–7], while at the N-terminus the unsatisfied backbone groups commonly form hydrogen bonds with side chains in the loop behind, as in the Asx/ST N-caps [8, 9], the capping box [10–16], and the big box [15]. At both ends of the helix, hydrophobic side chain interactions are also frequently found [17–23]. Electrostatic and hydrogen-bonding interactions between pairs of side chains are also present at helix termini, sometimes taking the place of hydrophobic interactions at the same positions [18, 22, 24–27].

Because the structures near the N- and C-termini can together span 8 residues of the helix, while the average helix has a length of about 12 residues [2], interactions near the termini make a substantial contribution to overall helix stability. Many studies have demonstrated the helix-stabilizing effects of interactions at helix termini [13, 16, 20–23, 28–40]. But terminal side chain interactions are not just selected to stabilize the helix; they are also likely to stabilize the extrahelical loops adjacent to the helix and help shape the loops into particular geometries that are important for structure and function. Terminal interactions can play important roles in the stabilization of key structural motifs such as the leucine zipper [41, 42], other coiled-coils [23], zinc fingers [43], and EF hands [44]. More generally, since the loops at both ends of a helix can constitute a substantial proportion of all residues found between pairs of helices or between helices and beta strands in a protein, local interactions near the helix terminus that shape the loop backbone may make very substantial contributions to the supersecondary and by extension overall tertiary structure of proteins [18]. Capping structures are also likely to play an important role in the process of protein folding [17, 29, 39, 45]. The importance of capping interactions to structure has been highlighted by the demonstration that the mutation of a single capping residue can cause misfolding leading to serious illness [46].

Side chain interactions near the helix terminus have been detected directly from structural surveys which identify and count motifs [2, 8, 9, 47], or indirectly, from studies which apply numerical analysis techniques to sequence data to find individual amino acids or patterns of amino acids that are overrepresented in structurally aligned sequence data. Early work in the second category ranked amino acids according to their frequencies of occurrence at different positions in secondary structure [48], and values of the propensities for amino acids to occur at individual positions near the helix boundary have subsequently been computed and updated [3, 18, 25, 26, 31, 49]. Motifs involving particular pairs of amino acids at specific positions near the helix terminus have been studied [18, 24–27, 49–51]. Some higher-order position-specific motifs involving three or more amino acids have also been analyzed [18, 20, 25–27, 50].

The extraction from sequence data of “first-order” motifs that involve single amino acids at particular positions is straightforward, since it requires only the calculation of the propensity of an amino acid to occur at a particular position in the structure. The propensity is the normalized frequency of occurrence of an amino acid at a particular position. This may be computed as the frequency of occurrence of the amino acid at the motif position divided by its frequency in the proteome as a whole. A directly related measure like the fractional overrepresentation may also be used (see Methods for details).

The detection of “second-order” motifs which specify pairs of amino acids at particular positions requires that the cooperative effect that results from the interaction between the amino acids in the pair be separated from the first-order structural effects produced by the presence of each individual member of the pair, using statistical modelling or an equivalent correlation analysis. The cooperativity associated with a motif reflects the interaction between the amino acids in the motif. Cooperativity can be either favourable, if the interaction stabilizes the structure or plays an important functional role, or unfavourable if the interaction destabilizes the structure. Motifs with favourable cooperativity can be detected in the dataset as patterns of amino acids which are overrepresented compared to expected counts for the patterns generated by null statistical models that represent the case of no interaction (see Methods for details). These motifs have abundances higher than their expected counts because their cooperativity confers an advantage which is selected for by evolution.

If the cooperativity associated with a pair motif is not separated from the underlying first-order effects, then it cannot be determined whether any overrepresentation of the motif is due to a genuine interaction or is merely an artefact of the overrepresentation of favourable first-order components. Some studies that have analyzed pair motifs [24, 27] have computed appropriate measures of pair cooperativity, but others have evaluated pair motifs using the same propensity methods used to evaluate single position motifs, or they have simply reported raw motif counts. Both of these techniques produce results that can mistakenly highlight artefacts generated by underlying first-order effects.

The detection of “higher-order” interactions between more than two amino acids is more involved than the pair detection procedure, because motifs involving more than two amino acids introduce multiple pairwise cooperative effects as well as higher-order effects into the data, and background models for the motifs must potentially include not only first-order effects, as is the case when evaluating pair motifs, but also pairwise or higher-order effects. Cascade Detection [27], a higher-order generalization of the pair detection method which is designed to evaluate the packet of cooperativity associated with multi-amino acid motifs using background models that include important underlying effects of all orders, was developed only recently, so work thus far on the detection of higher-order interactions from sequence data has relied on raw motif counts, propensity measures like those applied to first-order motifs [52], or background modelling with first-order effects alone [20]. The results obtained by these studies do not in general rigorously measure the cooperativity associated with higher-order motifs; they can instead highlight artefacts generated by underlying first-order effects and lower-order cooperative effects.

Past analyses have also not systematically addressed another key goal in the study of helix termini: the determination of how side chain interactions shape the backbone geometry of the loop structures at the helix ends. A detailed knowledge of the preferred loop geometries of particular side chain interactions will be necessary in order to efficiently utilize these interactions in the rational design of helix-terminal regions. Assuming that a side chain interaction plays a structural role, the loop conformations for which the interaction is likely to have the strongest stabilizing effect will be those in which the corresponding sequence motif has the greatest overrepresentation due to evolutionary selection. In order to extract detailed information from the structural database about how motifs are likely to shape the loop backbone, it is therefore necessary to map the distribution of the overrepresentation of each motif across the range of loop backbone geometries near the helix terminus. This has not yet been done. One study [53] has taken the important first step of partitioning the data by loop structure, but this work is not optimal for statistical motif detection (which the authors did not undertake), because it generates 900 clusters, which is too many for good statistics in each cluster. In addition, structures in this previous study are partitioned based on a combination of the dihedral angles in the loop and the angle formed between the loop backbone and the helical axis, while the clustering method described in the present work is based solely on the positional coordinates of the loop residues, which is the most natural representation for 3D mapping because it provides straightforward visualization.

In summary, a very substantial body of work, both analytical and experimental, has identified key helix-terminal motifs and has in some cases determined the degree to which motifs stabilize the helix. But higher-order cooperativities have not yet been comprehensively evaluated in the sequence data using methods designed to exclude artefacts generated by all lower-order effects. And most importantly, motifs have not yet been mapped to the particular loop geometries which they are likely to support. The present work addresses both of these goals. The study begins by applying a new least-squares 3D clustering algorithm to partition the helix-terminal loop structures in a large, recent dataset by loop backbone geometry and generate a set of structural exemplars that represents the range of loop conformations. The partitioning of the set of structures enables not only the mapping of known motifs to their preferred loop geometries, but also the detection of new motifs, since the more structurally specific motifs are overrepresented only in particular geometries, and therefore may not be detected by statistical methods applied to the global dataset. After partitioning, statistical motif detection is applied to the set of sequences in each cluster separately to compute the degree of overrepresentation of all sequence motifs up to order four with non-negligible abundances. Cascade Detection is utilized to evaluate the higher-order motifs while excluding artefact motifs produced by lower-order effects. The distribution of the abundance and overrepresentation of each motif is then projected onto the set of structural exemplars to produce CapMaps, which are 3D conformational heatmaps that reveal the loop conformations favoured by each motif. The side chain interaction, if any, that is associated with each significant motif is then characterized by the examination of structures from the clusters in which the motif is most overrepresented.

## Methods

### Data extraction

At the N-terminus, a set of 24,880 8-residue helix-terminal peptides was extracted from the set of all PDB chains with a threshold resolution of .20A, a maximum R-value of .20, and a maximum identity of 30 %. Since the peptides were extracted from the entire PDB, structural bias is minimized. In helix notation [3], the peptides encompass the positions *N'''*-*N''*-*N'*-*NCap*-*N1*-*N2*-*N3*-*N4*, where *N'''*, *N”* and *N'* are N-terminal to the helix, *NCap* is a partially helical transition residue, and *N1*…*N4* are the first four fully helical residues. The peptides were extracted from the chains by pattern matching the string XXNNHHHH, where H represents an α helical residue, N represents a non-helical residue (neither 3/10, α, nor pi), and X represents a residue with any structure. DSSP secondary structure assignments were used for the initial extraction, and the set of structures was then screened using the Ramachandran angle criteria for α helices as implemented in the Jmol program. This combined procedure was applied because examination of extracted examples of the common Asx and ST N-cap motifs revealed that the method produced alignments between sequence and helical structure that were superior to those produced by DSSP secondary structure assignments or PDB annotations alone, as judged by both backbone geometry and the hydrogen-bonding patterns of the motifs.

At the C-terminus, a set of 15,368 9-residue peptides covering the positions *C4*-*C3*-*C2*-*C1*-*CCap*-*C’*-*C”*-*C”’*-*C””* was extracted from the same set of PDB structures by pattern matching the string HHHHNNXXX.

### The helix-terminal coordinate system

The first step in processing the peptides was the establishment of a natural coordinate system for the helix terminus. The helical axis at each terminus was computed using the method due to Kahn [54]. Vectors ${\overrightarrow{V}}_1$ and ${\overrightarrow{V}}_2$ were established that bisect the angles *N1-N2-N3* and *N2-N3-N4* formed by the α carbons of the terminal helical residues. Since these vectors each pass through the helical axis and are perpendicular to it, the helical axis unit vector, which is chosen as the z-axis of the helix-terminal coordinate system, may be computed as:

1 $$ \widehat{Z}={\overrightarrow{V}}_2\times {\overrightarrow{V}}_1/\left|{\overrightarrow{V}}_2\times {\overrightarrow{V}}_1\right| $$

**Fig. 1:**
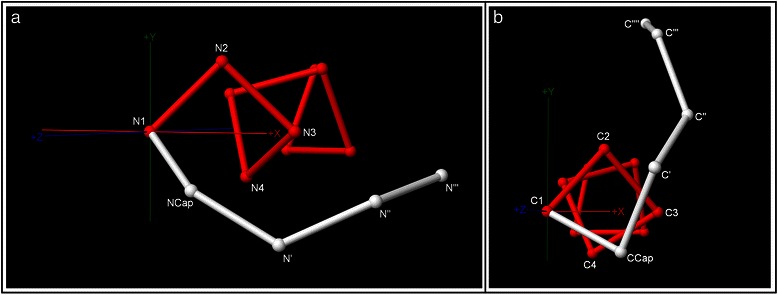
Helix-terminal coordinate systems at the N- and C-termini. For the N-terminus (**a**) and the C-terminus (**b**), an example loop backbone in white is plotted at its α carbons on the axes of the helix-terminal coordinate system, along with a 9 residue reference helix in red. In helix notation at the N- and C-termini respectively, the loop residues are *N'''*-*N''*-*N'*-*NCap*-*N1*-*N2*-*N3*-*N4* and *C4*-*C3*-*C2*-*C1*-*CCap*-*C’*-*C”*-*C”’*-*C””*. Note that the z-axis has a positive sense pointing outwards from the helix at each terminus

A natural origin for the helix-terminal coordinate system at the N-terminus is the location of the α carbon of *N1*, the first fully helical residue. The plane that contains this atom and is perpendicular to $\widehat{Z}$ is termed the “capping plane”. The x-axis of the cap coordinate system is defined to pass through the projection of the *N3* α carbon on the capping plane, and the right-handed coordinate system is completed by defining the y-axis as $\widehat{Y}=\widehat{Z}\times \widehat{X}$. An analogous procedure is used to establish the coordinate system at the C-terminus. Fig. 1 shows example N- and C-terminal loop backbone structures plotted at the α carbons in the helix-terminal coordinate systems at each end of the helix, with the loops shown in white displayed against reference helices in red.

### 3D backbone clustering

**Fig. 2:**
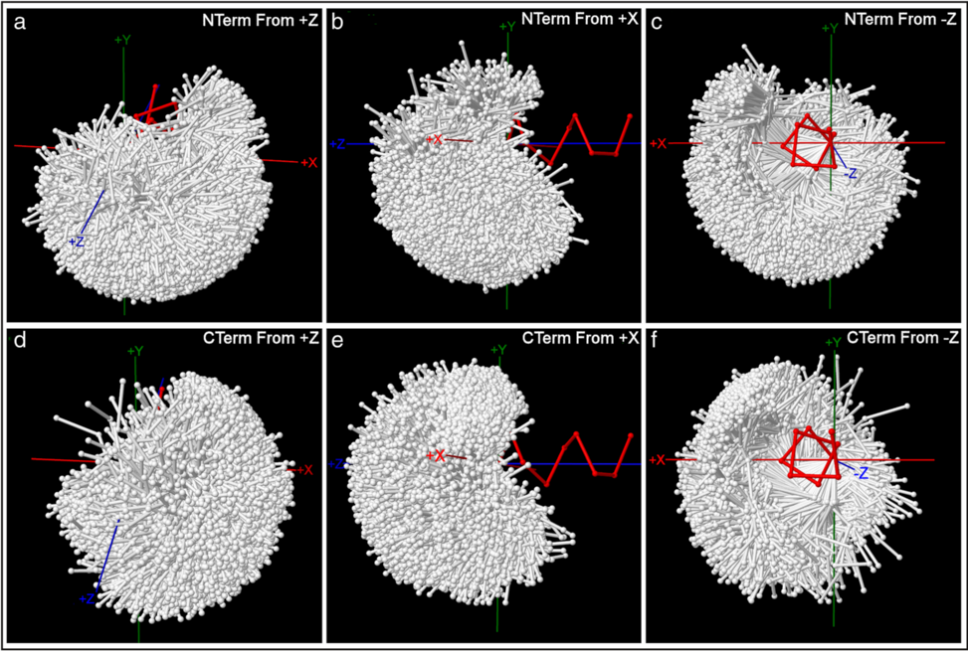
Complete datasets of helix termini. Loop backbone structures for the complete datasets of 24,880 N-terminal peptides (top row) and 15,368 C-terminal peptides (bottom row) are plotted at the α carbons in the helix-terminal coordinate systems for each terminus, and viewed from 3 perspectives in each case. At the N-terminus, perspectives are: (**a**) from (+z), (**b**) from (+x), and (**c**) from (−z). At the C-terminus, perspectives are: (**d**) from (+z), (**e**) from (+x), and (**f**) from (−z). At the N-terminus, loop residues *N'''*-*N''*-*N'*-*NCap* are plotted, while at the C-terminus loop residues *C”’*-*C”*-*C’*-*CCap* are shown. Loop structures show a broad range of geometries, with areas of higher density towards (+x, −y) at the N-terminus (ST-motifs, capping boxes) and (+x, +y) at the C-terminus (Schellman loops). Not all loop structures are visible, as some structures are concealed by others

Figure 2 shows the loop backbone structures of the complete datasets of 24,880 N-terminal and 15,368 C-terminal peptides, displayed in the helix-terminal coordinate systems and viewed from 3 perspectives at each terminus. For clarity of presentation, the simplifying approximation is made that the helical portion of all caps exhibits the same classical α helical conformation, so that the loops can be displayed against a single reference helix. Loop structures are partitioned using a clustering algorithm which finds a best-fit set of synthetic structural exemplars that represents the range of loop backbone conformations at the same time that it groups each actual loop structure with the nearest exemplar, with distance measured by the sum of squares of the distances between the α carbons of the structure and the synthetic α carbons of the exemplar. The set of exemplars is found via a perturbative search of the combined space of possible exemplar conformations which minimizes the total sum of squares:

2 $$ T={\displaystyle \sum_{e=1}^{N_e}{\displaystyle \sum_{s=1}^{N_{se}}{\displaystyle \sum_{\alpha =1}^{N_{\alpha }}\left({\overrightarrow{S}}_{s\alpha }-{\overrightarrow{E}}_{e\alpha}\right)}}}\bullet \left({\overrightarrow{S}}_{s\alpha }-{\overrightarrow{E}}_{e\alpha}\right) $$

Here, *e* indexes the *N*_*e*_ exemplars, *s* indexes the *N*_*se*_ loop structures clustered with each exemplar *e*, and α indexes the *N*_*α*_ α carbons in the loop structures and exemplars. ${\overrightarrow{S}}_{s\alpha }$ represents the position vectors of the α carbons in loop structure *s*, while ${\overrightarrow{E}}_{e\alpha }$ represents the position vectors of the α carbons in the exemplar nearest to *s. T* therefore measures the total sum of squares of the distances between all loop structures and their nearest exemplars, as measured at the α carbons.

At each perturbative step, an exemplar is randomly chosen and sent on a 3D random walk across its conformational space. The walk is implemented by applying small random changes to the coordinates of each synthetic α carbon in the exemplar while constraining the exemplar structure so that the distances between consecutive α carbons remain consistent with natural values near 3.8Å. As soon as a new exemplar conformation is found which produces a smaller value of *T*, the walk is terminated and the new conformation is saved as the current best conformation for that exemplar. Another exemplar is then chosen at random and perturbed with a random walk.

Cluster assignments are updated at every step of each random walk. At every step, for each actual loop structure, the sum of squares *T*_*e*_ is computed which measures the distance between the loop structure and each exemplar *e*:

3 $$ {T}_e={\displaystyle \sum_{\alpha =1}^{N_{\alpha }}\Big({\overrightarrow{S}}_{\alpha }}-{\overrightarrow{E}}_{e\alpha}\Big)\bullet \left({\overrightarrow{S}}_{\alpha }-{\overrightarrow{E}}_{e\alpha}\right) $$

All loop structures are then re-grouped with the exemplars which they fit best, as indicated by the smallest value of *T*_*e*_, before the the total *T* is calculated for each step. Because cluster assignments are continuously updated while the perturbative search is minimizing the global *T*, the exemplar conformations evolve toward the set of geometries which optimally partitions the cap structures into clusters in the sense that it minimizes the sum of squares of the distances between all loop structures and their nearest exemplars.

The question of how well the procedure converges towards a representative set of exemplar conformations is addressed by comparing the distances between sets of exemplars trained on the structural data with distances between sets of exemplars generated randomly. In all cases, the training is begun from different initial sets of exemplars which have been randomly perturbed. After training, the average distance between exemplar sets is much smaller than the average distance between randomly generated, untrained sets. For example, when 16 exemplars are generated per set, the average distance between pairs of exemplar sets formed between the 50 trained sets is 5.1 % of the average distance between pairs of sets formed between 1000 randomly generated sets, while for the 10 trained sets with the lowest values of T the average pairwise distance between trained sets drops to just .2 % of the random value. It is clear that as the exemplar sets are trained to fit the structural data better, they also move closer to a common set of exemplar structures, indicating that the clustering algorithm converges well as it reduces error.

At each terminus, 50 training runs are executed starting with different randomly perturbed initial sets of exemplar structures, and the trained set with the lowest value of T is selected. This set is used as a representative “basis set” of important helix-terminal conformations upon which motif detection results are projected. The number of exemplars specified in the clustering determines the number of clusters and the conformational resolution of the results. Higher degrees of conformational resolution are useful to more fully resolve the effects on the loop backbone of interactions that involve residues more distant from the helix, such as *N”* and *N”’*, because backbone structure diverges with each additional residue. But lower levels of resolution are also useful, because they can provide a clearer, summary picture of favoured loop conformations, most often for motifs involving residues within or close to the helix that favour wider ranges of loop conformations because they constrain the loop backbone before it has broadly diverged from the helix terminus. Clustering results may therefore be presented at two levels of conformational resolution, with each level adding one residue distal to the helix and doubling the number of clusters to represent the growing divergence. Results are presented for 3 loop residues with 16 clusters (3R/16C) and/or 4 loop residues with 32 clusters (4R/32C), covering α carbons {*N”*, *N’*, *NCap*} or {*N”’*, *N”*, *N’*, *NCap*} at the N-terminus and {*CCap*, *C’*, *C”*} or {*CCap*, *C’*, *C”*, *C”’*} at the C-terminus. Clustering was also run using α carbons out to *C””* at the C-terminus, but no results are included that involve residues beyond *C”’*, as analysis of interactions involving the first 4 loop residues amply covers the most common and important motifs. The numbers of clusters/exemplars (16 or 32) for each level of resolution were chosen as a balance between the competing requirements of larger numbers of clusters for better spatial coverage and smaller numbers of clusters to allow each cluster to contain enough sequences for good statistics.

**Fig. 3:**
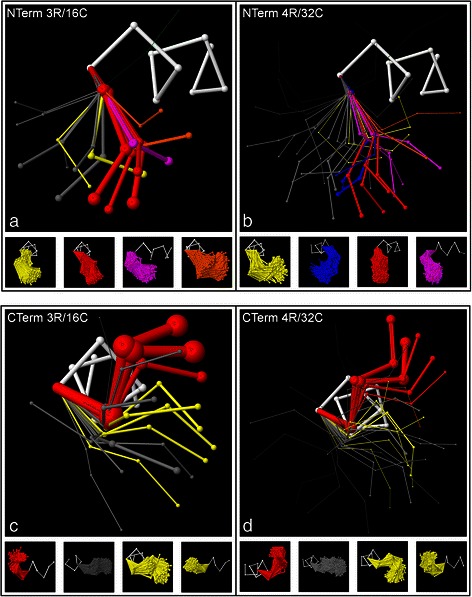
Best-fit synthetic structural exemplars with example clusters. Best-fit synthetic structural exemplars for the sets of all loop structures at the N-terminus (**a**, **b**) and C-terminus (**c**, **d**) of alpha helices, generated by 3D backbone clustering. At each terminus, a lower resolution plot that covers 3 loop residues with 16 exemplars (3R/16C) is shown at left, along with a higher resolution plot that extends coverage to 4 loop residues using 32 exemplars (4R/32C) at right. The width of each exemplar is proportional to the number of loop structures in its corresponding cluster. The synthetic exemplars capture the typical backbone geometries associated with abundant loop structures. At the N-terminus, these geometries are colour-coded in red for the “capping box”, purple for the “big box”, orange for the “expanded box”, yellow for beta-turns towards (+x), and blue for beta-turns towards (−x). At the C-terminus, the colours are red for the Schellman loop, yellow for beta-turns, and orange for combined Schellman loops and beta-turns. Samples of the clusters of actual loop structures that are associated with the exemplars are given below each exemplar plot using the same colour coding

The final conformation of each exemplar does not necessarily match any actual cap structure, but represents a mean geometry for its corresponding cluster. Since the exemplars do not need to correspond exactly to actual structures, they are not required to conform to detailed structural constraints for the backbone; the only constraint imposed is the distance constraint between successive α carbons. It is clear from the results, however, that the exemplars are constrained to generally realistic conformations by their relationship to the structural data, and many of the final exemplars exhibit loop backbone geometries that correspond to commonly seen structural features. This is demonstrated in Fig. 3, which presents plots of the final trained 3 residue/16 cluster (3R/16C) and 4 residue/32 cluster (4R/32C) exemplar sets, along with examples of the clusters of actual loop structures that are grouped with the exemplars. In these plots, the width of each exemplar corresponds to the size of its corresponding cluster, and the plots are colour coded to highlight the loop backbone geometries that correspond to common structures – see the figure caption for details.

### Statistical motif detection

Once the cap structures have been partitioned into clusters by geometry, statistical motif detection is applied to the sets of sequences in each cluster separately to determine which sequence motifs are most overrepresented in each geometry. The extraction of “first-order” sequence motifs that specify particular amino acids at particular sequence positions is straightforward. An appropriate null model for the occurrence of an amino acid of type *i* at a sequence position specifies that the amino acid’s probability of occurrence is equal to its abundance fraction in the proteome as a whole:

4 $$ {P}_i=\frac{N_i}{{\displaystyle \sum_j{N}_j}} $$

where *N*_*i*_ is the number of occurrences of amino acid *i* in the proteome, and *j* ranges over all 20 amino acid types. A *p*-value to measure the statistical significance of the abundance of an amino acid at a particular position is computed using a binomial calculation in which *P*_*i*_ is the probability parameter and the total number of structures in the cluster is the number of binomial trials *N*. The expected count for a first-order motif in a cluster is computed as *E*_*i*_ = *NP*_*i*_, where *N* represents the total number of structures in the cluster. The degree of overrepresentation of the motif, which is used to measure the motif’s importance, is computed as (*O*_*i*_ − *E*_*i*_)/*E*_*i*_, where *O*_*i*_ is the motif’s observed count. This is the fractional overrepresentation of the motif above its expected count. This measure is equivalent to the excess of the motif’s global propensity, or the difference between the global propensity, which is the motif’s frequency of occurrence at its particular position divided by its frequency in the entire proteome, and unity.

Second-order motifs, which specify particular pairs of amino acids at particular pairs of positions, are evaluated by constructing a 2x2 contingency table for each pair, with dimensions that represent sequence positions and categories in each dimension that represent the presence or absence of each amino acid at each position. A *p*-value that measures the significance of the two-factor effect that corresponds to cooperativity between the two amino acids is computed with Fisher’s Exact Test [55]. An expected count for the motif is calculated as the expected count in the cell of the contingency table that corresponds to the presence of both amino acids at both positions, using standard techniques in contingency table analysis. The degree of overrepresentation of the motif is computed as (*O*_*i*_ − *E*_*i*_)/*E*_*i*_, the fractional overrepresentation of the motif, where *E*_*i*_ is the expected count in the cell and *O*_*i*_ is the observed cell count.

Higher-order motifs, which specify more than two amino acids at particular positions, are evaluated using Cascade Detection (CD) [27], a higher-order generalization of the contingency table method for detecting pair motifs which detects the packet of cooperative effects of all orders introduced by each motif. CD combines iterative smoothing of the motif in its (higher-order) contingency table with an exhaustive evaluation of all possible background models for the table to identify the simplest background model (smallest number of fixed degrees of freedom) which fits the data well after the motif itself has been removed from the data by smoothing using the background model, in a self-consistent procedure. The expected count that CD generates for the motif is the expected count in the cell of the motif’s contingency table that corresponds to the presence of all of the amino acids in the motif. CD also generates a *p*-value that measures the significance of the packet of cooperative effects associated with the motif. The motif’s degree of overrepresentation is computed as (*O*_*i*_ − *E*_*i*_)/*E*_*i*_, the fractional overrepresentation, where *E*_*i*_ is the expected count in the motif’s cell and *O*_*i*_ is the observed cell count.

### Mapping

CD is applied separately to the sequence sets in each cluster to compute the distributions across loop geometries of the fractional overrepresentations of all sequence motifs of orders up to 4 that have non-negligible abundance. For each motif that is found to be substantially overrepresented in at least one individual cluster or in the complete sequence set, a CapMap is generated. A CapMap is a 3D, manipulatable conformational heatmap which displays the distribution of the motif’s abundance and overrepresentation across the range of loop backbone conformations. The CapMap is a Jmol structure which displays the set of structural exemplars derived by 3D backbone clustering and shown in Fig. 3, with the thickness of each exemplar made proportional to the abundance of the motif in the corresponding cluster, and the colour of each exemplar made to represent the fractional overrepresentation of the motif in the cluster. The exemplar colours blue → purple → red → yellow indicate increasing motif overrepresentation. Colours on this scale do not correspond to the same degrees of overrepresentation across all maps, but instead depict relative overrepresentation across the exemplars within each map to allow the maximum representational bandwidth for each motif in the presence of motifs with widely varying overrepresentations. Two types of CapMaps are presented: low resolution maps cover three loop residues using 16 clusters (3R/16C), while high resolution maps cover 4 loop residues using 32 clusters (4R/32C).

CapMaps reveal the loop backbone geometries that are most favoured by each sequence motif. Once the favoured geometries are identified, actual structures from the clusters corresponding to these geometries are examined in a structure browser to characterize the side chain interaction, if any, that is responsible for the motif’s overrepresentation.

## Results

CapMaps for thousands of overrepresented motifs were generated for each terminus; only a selection of the most significant and interesting motifs are presented here. Low resolution 3 residue/16 cluster (3R/16C) or high resolution 4 residue/32 cluster (4R/32C) maps are shown for each motif, depending on which gives the clearest structural picture. The CapMap maps the distribution of motif abundance and overrepresentation across loop geometries at each terminus by representing motif abundance as the thicknesses of the structural exemplars generated by clustering, and motif overrepresentation as the colour of these exemplars. Only those structural exemplars that correspond to clusters in which a motif is overrepresented are shown in each map. Beneath each map in a figure, an example structure is given which shows the side chain interaction that underlies the motif. The structure is taken from the cluster corresponding to the exemplar marked with an asterisk on the map, which is usually the cluster in which the motif exhibits the greatest overrepresentation, but may be another overrepresented cluster if the cluster with peak overrepresentation has a very low abundance. Beneath the example structure for each motif, values of the motif abundance (with percent abundance in parentheses), fractional overrepresentation, and motif *p*-value are given for the global dataset on the top line, and for the cluster highlighted with an asterisk on the line below. *P*-values down to 1E-10 are given; values lower than this are marked as zero. *P*-values are not controlled for multiple testing; it is left to individual researchers to adjust their threshold for statistical significance depending on whether they are focusing on one or multiple motifs. The bottom text box beneath each map in a figure contains a brief description of the structure associated with the motif.

Additional file 1 lists up to 20 examples from the PDB for each sequence motif given here, with the examples taken from clusters in which the motif is highly overrepresented. It should be noted that a corresponding structural motif is not present for all examples of a sequence motif – each sequence motif must be checked for the presence of a structural motif using a structure browser.

To aid in concise presentation, the following abbreviations are used below: SC for side chain, MC for main chain, MCA for main chain amide, and MCC for main chain carbonyl. Motifs are named by specifying the position in helix notation in italics followed by the one-letter abbreviation for the amino acid in standard type, for each amino acid in the motif: *NCap*T-*N3*Q specifies the motif with threonine at the *NCap* position and glutamine at the *N3* position, for example. Main chain groups are named with the position in italics followed by the abbreviation for the group type: *N’*MCC specifies the main chain carbonyl at *N’*, for example.

Motifs are described in some detail below, but the results can also be browsed as a “story in pictures” by referring just to the captions and motif descriptions within the figures.

### First-order polar motifs at the N-terminus

**Fig. 4:**
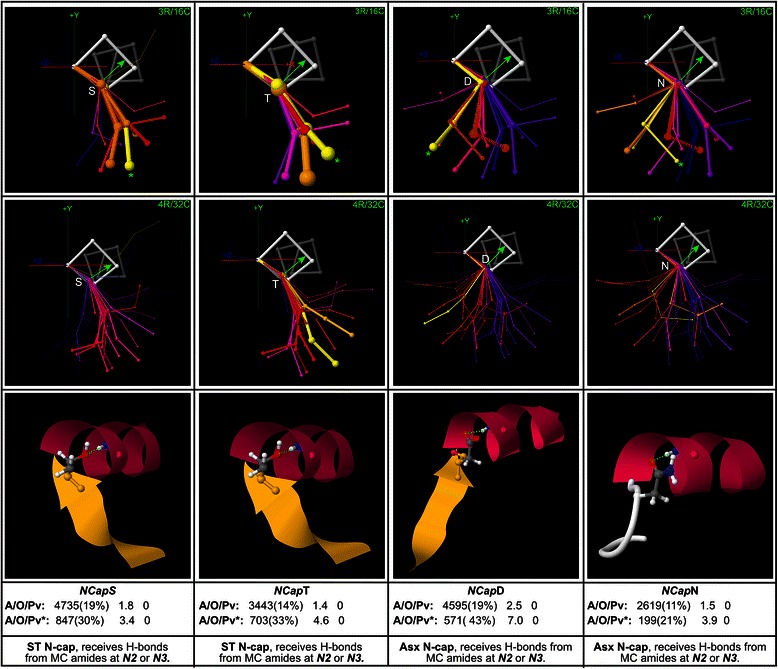
Asx/ST N-cap motifs. Low resolution (3R/16C) and high resolution (4R/32C) maps for the Asx and ST N-cap motifs, with example structures and global and peak-cluster (*) motif data (Abundance/Overrepresentation/Pvalue). Exemplar width is proportional to motif abundance in the corresponding cluster/geometry, while exemplar colour is proportional to motif overrepresentation. These motifs, in which {Asp, Asn} (Asx), or {Ser, Thr} (ST) are found at the *NCap* position where the SC forms capping H-bonds with the MCCs at *N2* or *N3*, are the most important structural features at the N-terminus. The maps reveal that the two motif classes show peak overrepresentation in geometries that are largely complementary, with ST orienting the *NCap → N’* vector generally toward (+x, −y), while Asx orients the vector generally towards (−x, −y). This complementarity suggests that the Asx and ST N-cap motifs are utilized in distinct structural roles. Structural variations are also evident between the members of each motif class (see main text)

The Asx and ST N-cap motifs [8, 9], in which {D, N} (Asx), or {S, T} (ST) are found at the *NCap* position, where the SC receives capping H-bonds from the MCAs at *N2* or *N3*, are the most important structural features at the N-terminus. These motifs are mapped in Fig. 4. The low resolution, 3R/16C maps reveal that the two motif classes show peak overrepresentation in geometries that are largely complementary, with ST orienting the *NCap → N’* vector generally toward (+x,-y), while Asx orients the vector generally towards (−x,-y). This complementarity suggests distinct structural roles for the Asx and ST N-caps. At the higher conformational resolution of the 4R/32C maps, clear structural differences emerge between the separate members of each motif class. *NCap*T shows its greatest overrepresentation in extended geometries towards (+x, −y) (yellow), which may be related to hydrophobic or VDW interactions between Thr’s SC methyl group, which orients in the direction of these loop conformations, and the body of the protein external to the terminus, into which these conformations often project. By contrast, *NCap*S is less pronounced in these geometries, showing its greatest overrepresentation in extended geometries towards (−y) (red, pink) and in a beta-turn geometry towards (+x) (red), which Ser favours because its SC can form H-bonds with the *N”’*MCC, supporting the turn.

Structural differences are also evident within the Asx motif, with *NCap*N favouring right-handed beta-turn conformations (yellow) to a greater extent than *NCap*D. This may be because these beta-turns towards (+x) are secured by *NCap*MCA → *N”’*MCC H-bonds, and Asp’s negative charge at *NCap* may tend to disrupt these bonds by repelling the *N”’*MCC.

Geometry-specific Asx/ST motifs also occur at *N”’* (Additional file 2), and *N’* and *N”* (Additional file 3). Other important first-order polar motifs at the N-terminus are mapped in Additional file 4.

### Proline motifs at the N-terminus

**Fig. 5:**
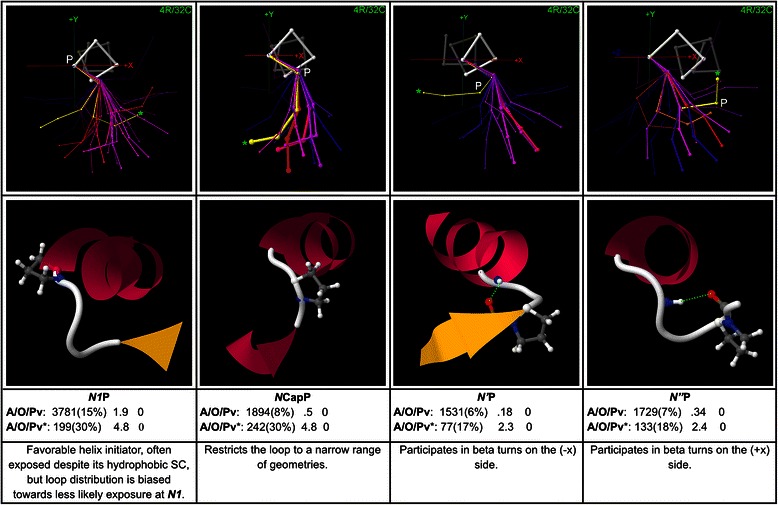
First-order proline motifs from *N1* to *N”*. First-order proline motifs that occur from *N1* to *N”* are shown, with example structures and global and peak-cluster (*) motif data (Abundance/Overrepresentation/Pvalue). Exemplar width is proportional to motif abundance in the corresponding cluster/geometry, while exemplar colour is proportional to overrepresentation. Proline’s rigid pyrrolidine ring favours particular loop geometries that depend on the loop position at which it occurs

Proline, with its unique pyrrolidine ring SC which incorporates the backbone and provides main-chain rigidity, plays significant roles at multiple positions near the N-terminus. First-order proline motifs at positions *N1* → *N”* are mapped in Fig. 5. Judged by overrepresentation and abundance, *N1*P is of similar importance to the Asx and ST N-cap motifs, occurring in 15 % of all caps in the global set, nearly three times as often as expected. After the Asx/ST motifs, Pro is also the most important amino acid at *NCap*, where its cyclic SC restricts the backbone to a narrow range of geometries towards (−y). *N’*P is also a geometry-specific motif, peaking in a geometry in which Pro’s ring supports tight turns towards (−x), some of which constitute beta turns stabilized by *N1*MCA → *N”*MCC H-bonds. Proline is known to strongly support beta-turns when it falls at the (*i* + 1) position in the turn (which corresponds to *N’* here), because its cis isomer sets up the backbone turn well for the beta-turn H-bond [56]. *N”*P supports turns oriented in the opposite direction from those favoured by *N’*P, peaking in the geometries towards (+x) that typically correspond to beta-turns stabilized by *NCap*MCA → *N”’*MCC H-bonds (yellow exemplar). *N”’*P, mapped in Additional file 5, is also geometry-specific, favouring geometries towards (−y) in which it participates in beta-turns stabilized by *N’*MCA → *N””*MCC H-bonds.

### Capping box motifs

The combination of the common *N3*MCA → *NCap*SC Asx/ST capping H-bond and a reciprocal *NCap*MCA → *N3*SC H-bond has been dubbed the “capping box” [10]. When the reciprocal H-bond originates at *N’* instead of *NCap*, the “big box” results [15]. Since the SC at *N3* may instead interact with MC polar groups N-terminal to *N’*, and may interact with main-chain amides (MCAs) instead of main-chain carbonyls (MCCs), it is useful to expand and systematize this vernacular “box” notation to cover such “expanded boxes”. In this expanded system, H-bond pairs at the N-terminus are classified by indicating the position of the polar group that interacts with the *N3* SC, followed by the type of the polar group. The classic capping box is then termed a “*NCap* amide box”, while the classic big box is termed a “*N’* amide box”, and an H-bond pair in which the *N3* SC bonds with the *N”*MCC is termed a “*N”* carbonyl box”.

**Fig. 6:**
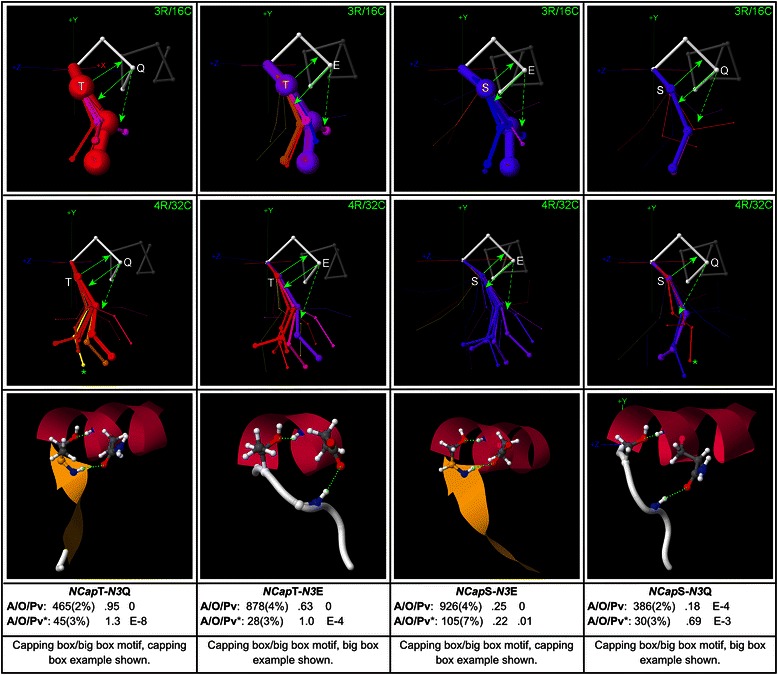
Capping boxes. Capping-box motifs with example structures and global and peak-cluster (*) motif data (Abundance/Overrepresentation/Pvalue). Exemplar widths are proportional to motif abundance in the corresponding cluster/geometry, while exemplar colour is proportional to overrepresentation. Both low resolution (3R/16C) and high resolution (4R/32C) maps are shown, with examples of both classic capping boxes (with solid green arrows) and big boxes (with dashed green arrows for the SC interaction from *N3*). The capping box and big box geometries form two distinct arms of a “pincer”, in which the capping box arm positions *N”* more towards (+z), while the big box arm positions it more towards (−z), setting up the *N’* MCA for interaction with the *N3* SC. These maps show that motifs with *NCap*T show substantially higher overrepresentation than those with *NCap*S

The maps for *NCap*T-*N3*Q, *NCap*T-*N3*E, *NCap*S-*N3*E, and *NCap*S-*N3*Q (Fig. 6) are examples of sequence motifs that exhibit the capping box or big box. Both 3R/16C and 4R/32C maps are shown for each motif. The peak geometries that bring the loop at *N”* back towards (−z) (shown most clearly in the 4R/32C maps for *NCap*T-*N3*Q and *NCap*T-*N3*E) correspond to the optimum geometries for big boxes, since these geometries position the *N’* MCA closer to *N3* for optimal interaction with the SC from that position. Classic capping boxes occur in the other abundant peak geometries.

Although the aggregate global abundance of the most common capping box sequence motifs with *Ncap*S is about the same as the aggregate abundance of the common capping box sequence motifs with *Ncap*T, with each occurring in about 8 % of all termini in the global set, the maps reveal that the abundant capping box sequence motifs with *Ncap*T are substantially more overrepresented than those with *Ncap*S. Many actual capping box and big box structures with *NCap*S are nevertheless observed, but since cooperativity is much lower the stabilizing effect of these structures is probably due less to cooperativity between the reciprocal interactions and more to the effects of each individual underlying H-bond than is the case for motifs with *NCap*T.

Examples are also mapped of motifs with polar residues at *NCap* and *N3* which tend to form structures other than the classic capping box or big box, including expanded *N”* carbonyl boxes in Additional file 6, and the SC/SC motifs *NCap*D-*N3*T and *NCap*D-*N3*S in Additional file 7.

### Other polar side chain/side chain motifs at the N-terminus

**Fig. 7:**
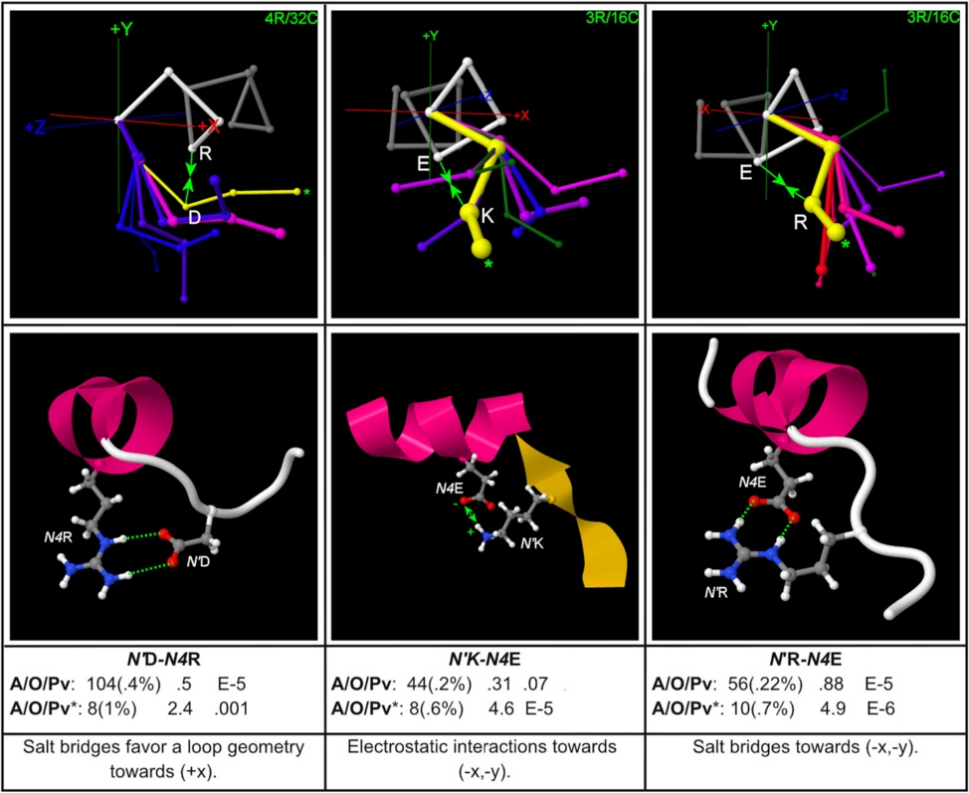
Polar pairs at (*N’*, *N4*). Examples of polar pair motifs at the positions (*N’*, *N4*) are shown, with example structures and global and peak-cluster (*) motif data (Abundance/Overrepresentation/Pvalue). Exemplar widths are proportional to motif abundance in the corresponding cluster/geometry, while exemplar colour is proportional to overrepresentation. These motifs correspond to electrostatic interactions and salt bridges

Polar SC/SC motifs at positions (*N4*, *N’*), including electrostatic interactions and salt bridges, are mapped in Fig. 7. Additional N-terminal polar SC/SC interactions, including salt bridges and electrostatic triplets, are mapped in Additional file 8 and Additional file 9.

### Hydrophobic pair motifs at the N-terminus

**Fig. 8:**
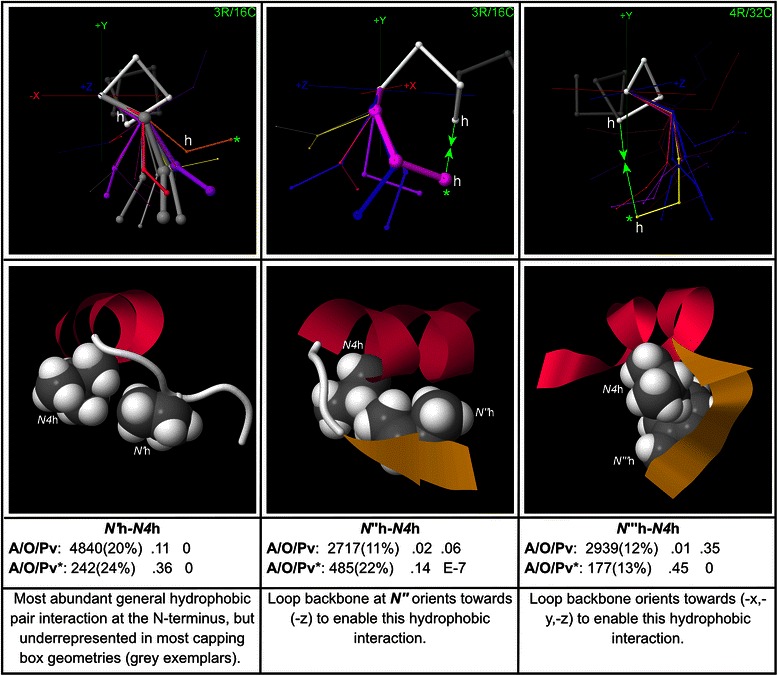
General hydrophobic pair motifs at the N-terminus. Motifs detected after a general hydrophobic residue identifier (‘h’) is substituted for the hydrophobic residues {Phe, Leu, Val, Met, Ile}, shown with example structures and global and peak-cluster (*) motif data (Abundance/Overrepresentation/Pvalue). Exemplar width is proportional to motif abundance in the corresponding cluster/geometry, while exemplar colour is proportional to overrepresentation. For *N’*h-*N4*h, all exemplars are shown, including those that correspond to clusters in which the motif is underrepresented, in order to emphasize that the motif is generally underrepresented in the capping box geometries. The map for *N”*h-*N4*h reveals the backbone turn at *N’* that positions the *N”* SC for hydrophobic interaction with the *N4* SC

When a general hydrophobic residue identifier (labelled ‘h’) is substituted into the sequence data in the place of each of the most hydrophobic amino acids {F, L, V, M, I}, the important general hydrophobic cooperativities are detected (Fig. 8). *N’*h-*N4*h [18], which combines the two positions at which hydrophobic residues are most often seen as first-order motifs, is the most abundant general hydrophobic pair at the N-terminus in the global dataset, appearing in 20 % of all instances, and it has the lowest *p*-value. It is interesting to note that the map for *N’*h-*N4*h shows peak cooperativity in geometries to either side of the peak capping box geometries (which are mapped in Fig. 6 above), while showing underrepresentation in most of the capping box geometries themselves (shown in grey in the *N’*h-*N4*h map). This may be due to a steric clash between bulky hydrophobic SCs at *N’* in *N’*h-*N4*h and the *N3* SC participating in the *NCap*MCA ***→*** *N3*SC H-bond in capping boxes. However, it is important to note that there are nevertheless many instances of capping boxes occurring together with hydrophobic pairs in these geometries due to the overrepresentations of the capping box and the hydrophobic pair as individual motifs, and the fact that some particular hydrophobic pairs do show overrepresentation in these geometries.

Particular instances of hydrophobic pair motifs are mapped in Additional file 10. Hydrophobic pairs at (*N”*, *N4*) favour geometries that bring the loop backbone at *N”* back towards (−z), facilitating the interaction with the SC at *N4*. Higher-order interactions with hydrophobic components are mapped in Additional file 11. Aromatic pair motifs are mapped in Additional file 12, and aromatic-proline motifs in Additional file 13.

### Ligand binding and active site motifs at the N-terminus

**Fig. 9:**
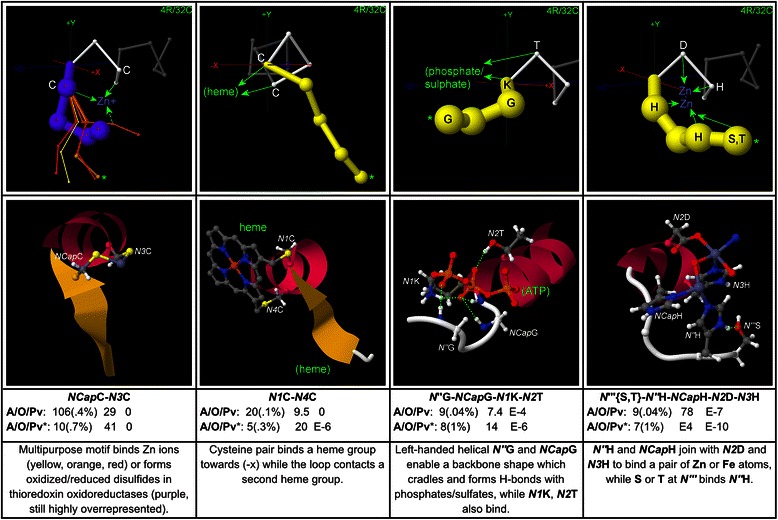
Ligand-binding/active site motifs at the N-terminus. Four motifs that represent ligand binding or active sites are shown, with example structures and global and peak-cluster (*) motif data (Abundance/Overrepresentation/Pvalue). Exemplar width is proportional to motif abundance in the corresponding cluster/geometry, while exemplar colour is proportional to overrepresentation. The CxxC motif (*NCap*C-*N3*C), which is involved in Zn binding and oxidation/reduction, shows distinct geometries for each function. Also shown is *N”*G-*NCap*G-*N1*K-*N2*T, which forms a “cradle” that binds phosphate/sulphate groups

Examples of ligand binding and active site motifs are mapped in Fig. 9. The map for the N-terminal CxxC motif, which is a key structure involved in both metal ion binding and oxidation-reduction, clearly shows the motif’s distinct loop geometries for each function.

### Other motifs at the N-terminus

Glycine is important in peptide structure because it provides chain flexibility, including the ability to easily take on a left-handed helical conformation, as well as a minimal SC which is useful where close packing is required. Three triplet N-terminal motifs with glycine are mapped in Additional file 14, including *NCap*H-*N2*G-*N3*H, which could be dubbed the “HH” box, in which two His residues stack in near-planar fashion while Gly allows packing room for one of them. A selection of motifs that promote beta-turns are mapped in Additional file 15.

### C-terminal motifs: glycine

**Fig. 10:**
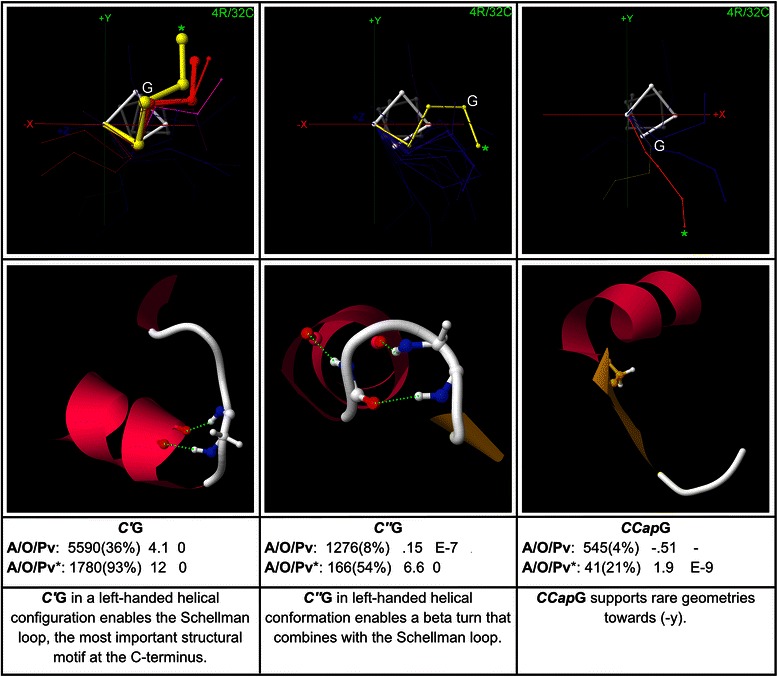
Glycine motifs at the C-terminus. Three C-terminal motifs with glycine are shown, with example structures and global and peak-cluster (*) motif data (Abundance/Overrepresentation/Pvalue). Exemplar width is proportional to motif abundance in the corresponding cluster/geometry, while exemplar colour is proportional to overrepresentation. *C’*G is the most important of all C-terminal motifs, because it enables Schellman loops with its left-handed alpha conformation

C-terminal glycine motifs are mapped in Fig. 10. *C’*G is the most important of all C-terminal motifs, because it commonly assumes a left-handed helical conformation which is favourable for the formation of a *C”*MCA → *C3*MCC H-bond, which together with a *C’*MCA → *C2*MCC H-bond secures the backbone in the very common Schellman loop conformation. Several exemplars represent Schellman loops (yellow, orange, purple in the *C’*G map). *C’*G shows maximum overrepresentation in the Schellman loop geometry that brings the backbone most towards (+y).

In *C”*G, Gly in a left-handed helical conformation enables the loop to turn towards (−y, +z). In this geometry, the *C”’*MCA can donate an H-bond to the *CCap*MCC, frequently forming a combined beta-turn and Schellman loop.

### Proline motifs at the C-terminus

**Fig. 11:**
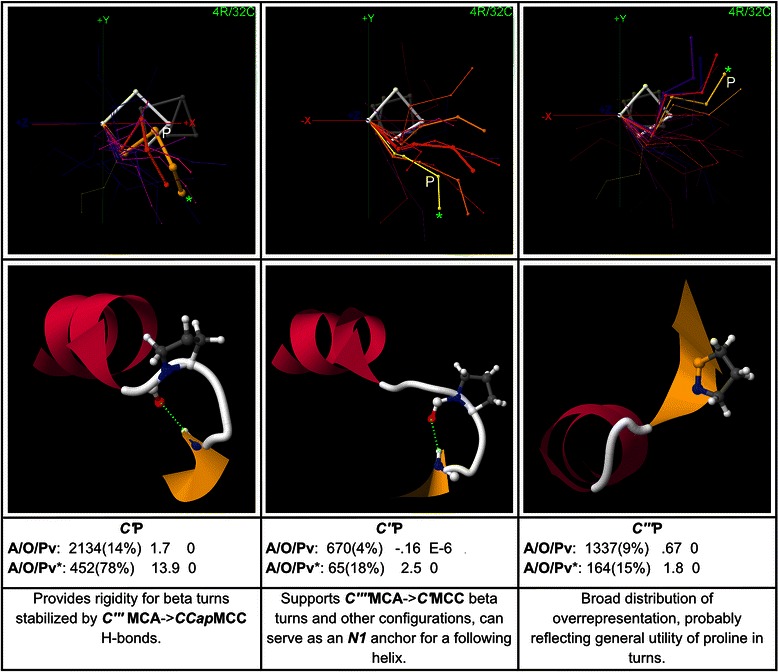
Proline motifs at the C-terminus. Three C-terminal motifs with proline are mapped, with example structures and global and peak-cluster (*) motif data (Abundance/Overrepresentation/Pvalue). Exemplar width is proportional to motif abundance in the corresponding cluster/geometry, while exemplar colour is proportional to overrepresentation. *C’*P, which provides rigidity for beta-turns stabilized by *C”’*MCA- > *CCap*MCC H-bonds, is second only to *C’*G in importance at the C-terminus

C-terminal proline motifs are mapped in Fig. 11. *C’*P, which is highly overrepresented in the global dataset, occurs in 14 % of all termini and is second only to *C’*G in importance at the C-terminus. *C’*P’s map shows that its importance is due mainly to the rigidity that it provides at *C’* in support of beta-turns that are stabilized by *C”’*MCA → *CCap*MCC H-bonds. *C”*P and *C”’*P are also mapped in Fig. 11.

Higher-order motifs with proline, including triplets, are mapped in Additional file 16.

### Polar interactions at the C-terminus

**Fig. 12:**
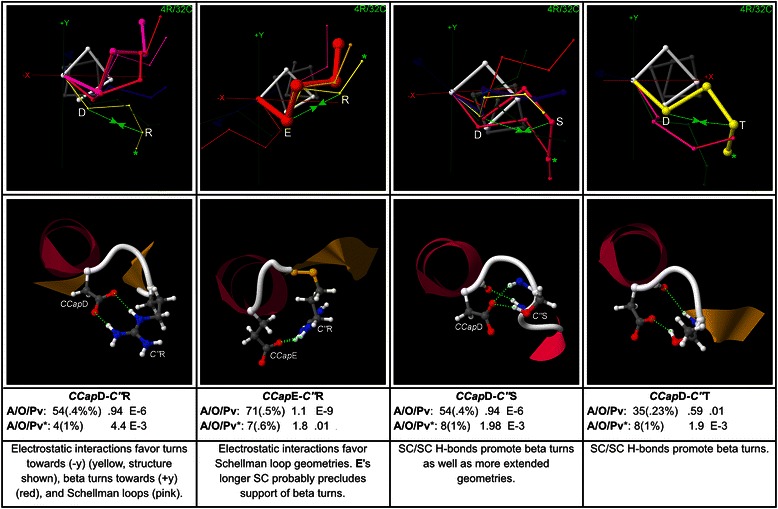
Polar pair motifs at (*CCap*, *C”*). Four polar pair motifs at (*CCap*, *C”*) are mapped, with example structures and global and peak-cluster (*) motif data (Abundance/Overrepresentation/Pvalue). Exemplar width is proportional to motif abundance in the corresponding cluster/geometry, while exemplar colour is proportional to overrepresentation. These pairs support Schellman loops and beta- and other turns with SC/SC H-bonds, electrostatic interactions, and salt bridges

Polar pair interactions at the C-terminus, including electrostatic and H-bonded pairs, are mapped in Fig. 12, with additional polar pairs, including electrostatic interactions and salt bridges that support Schellman loops, mapped in Additional file 17. An electrostatic triplet is mapped in Additional file 18.

Although the polar amino acids {Asp, Asn, Ser, Thr} are much less important at *CCap* than they are at *NCap* where they form the Asx and ST N-cap motifs, they nevertheless can play structural roles, including capping the helix and reinforcing turns, as mapped in Additional file 19. More first-order polar motifs, including hydrophilic motifs and capping interactions, are mapped in Additional file 20.

### Aromatic motifs at the C-terminus

**Fig. 13:**
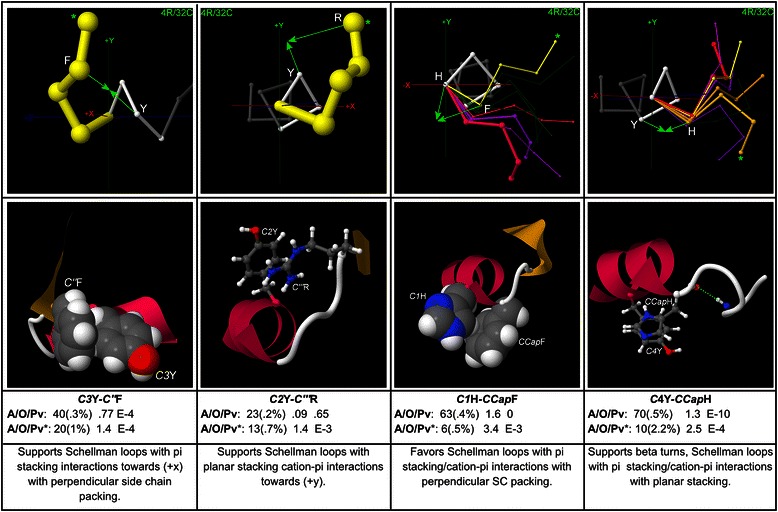
Aromatic pair interactions at the C-terminus. Examples of aromatic pair interactions at the C-terminus are mapped, with example structures and global and peak-cluster (*) motif data (Abundance/Overrepresentation/Pvalue). Exemplar width is proportional to motif abundance in the corresponding cluster/geometry, while exemplar colour is proportional to overrepresentation. These interactions include pi-stacking in parallel (*C4*Y-*CCap*H) and perpendicular (*C3*Y-*C”*F) orientations, as well as cation-pi interactions (*C2*Y-*C”’*R, *C1*H-*CCap*F)

Aromatic pair interactions at the C-terminus can support Schellman loops and beta-turns with both planar and perpendicular stacking, as mapped in Fig. 13.

First-order aromatic motifs at the C-terminus can pack between hydrophobic SCs in the helix and loop, strongly favouring beta-turns, as mapped in Additional file 21.

## Discussion

This analysis maps the favoured loop backbone geometries for previously identified helix-terminal SC interactions, including the Asx/ST N-cap motifs, which were first identified by structural surveys [8, 9] (Fig. 4), the *N1* proline motif identified via structural survey and propensity analysis [18] (Fig. 5), the capping box [10] and big box [15] motifs identified via sequence and structural surveys (Fig. 6), hydrophobic pair motifs identified via propensity analysis and structural surveys [18] (Fig. 8), and the CxxC [43] (Fig. 9) and *C’*G/Schellman motifs [4] (Fig. 10) identified via structural surveys. In addition, many interactions that as far as the author is aware have not been previously highlighted have been detected and mapped, including Asx/ST motifs in the loop outside of *NCap* (Additional files 2 and 3), proline motifs in the loop outside of *N1* (Fig. 5), “expanded” box motifs (Additional file 6), additional polar interactions at the N-terminus (Additional files 8 and 9), N-terminal aromatic pair and aromatic-proline interactions (Additional files 12 and 13), C-terminal glycine motifs outside of *C’* (Fig. 10), C-terminal proline motifs (Fig. 11), C-terminal polar interactions (Fig. 12 and Additional files 17, 18 and 20), C-terminal motifs with {Asp, Asn, Ser, Thr} (Additional file 19), and C-terminal aromatic interactions (Fig. 13 and Additional file 21).

The fact that many of the motifs detected here show peak overrepresentation in geometries that can be easily rationalized as favourable for SC interactions associated with the motif, and are indeed found to correspond to such interactions in the structural data, justifies the approach of mining structurally partitioned sequence data using statistical motif detection in the sequence space. The importance of partitioning is emphasized by the observation that many motifs which are overrepresented in the loop geometries that are favourable for their corresponding SC interactions are not overrepresented in the global dataset, so that if the sequence data were not partitioned, these motifs would not be detected due to the masking effect of the larger dataset. The detection of new motifs is also enabled by the evaluation of higher-order cooperativities using Cascade Detection.

The results presented here should be interpreted in light of several caveats. In the first place, it should of course not be assumed that a SC interaction that is shown to be overrepresented in a particular loop conformation will be compatible with that conformation in all structures. Motifs do not exist in a vacuum; neighbouring amino acids external to the motif may interfere with the interaction. This caveat has particular validity for motifs with very low abundances, since in these cases the motif is less reliably general, because it has not been demonstrated to be robust in the presence of a variety of adjacent residues. The examples that are provided for each motif in Additional file 1 should be useful in determining which neighbouring amino acids are compatible; when these examples contain the corresponding SC interaction they provide examples of neighbouring residues that do not interfere with the interaction.

An important related issue is the nature of the external environment of the terminus, including the degree and configuration of its exposure to solvent. In this study, structures were not partitioned by environment type, since it was judged that this would add too much complexity to this initial broad and comprehensive analysis. The motif examples provided with each map can help here also, since the environments of the examples of a motif can be evaluated to determine whether a motif is suitable for use in a desired context.

## Conclusions

This work applies structural clustering, statistical motif detection, and 3D conformational mapping to comprehensively detect and map the SC interactions near helix termini which have been favourably selected by evolution because they stabilize important structures or play direct functional roles. The library of geometry-specific SC interactions identified here provides a new and more detailed picture of peptide structure near helix termini that constitutes a significant contribution to the structural biochemistry of proteins. This library should prove useful in the rational optimization and design of helix caps and helix-terminal loops (and by extension supersecondary and tertiary structures), because it identifies many of the side chain interactions which are used by nature to stabilize particular helix-terminal loop geometries. These interactions constitute good candidates for inclusion in the designs of synthetic helix terminal loops with similar geometries.

Although only a subset of the detected motifs is presented here, maps for all significantly overrepresented motifs are available from the author, and the author can also provide larger sets of PDB examples for many motifs. A goal of future work is to make a wide selection of CapMaps available in an online database that is structurally addressable, so that designers seeking to stabilize a particular loop structure can retrieve all SC interactions that support that structure, along with motif examples from the PDB that include compatible neighbouring residues. Any researcher or organization with interest in a database project should contact the author.

This analysis sets the stage for future work. The combination of structural partitioning, statistical motif detection, and 3D conformational mapping that is used here can also be applied to map SC interactions associated with other common structural components of proteins such as beta-turns, gamma turns, and beta bulge loops, in order to construct as complete a picture as possible of how SC interactions may shape the peptide backbone.

### Availability of supporting data

All data supporting this work are available from the PDB, http://www.rcsb.org/pdb/home/home.do.

## Additional files

Additional file 1:
**Motif example file.** This file contains references to example structures from the PDB for each sequence motif given in the results. Up to 20 examples are provided for each motif. Complete lists of all examples for each motif are available from the author.
Additional file 2:
**Asx/ST motifs at** ***N”’*** **.** Maps for Asx and ST motifs that occur at *N”’*, with example structures and global and peak-cluster (*) motif data (Abundance/Overrepresentation/Pvalue). Exemplar width is proportional to motif abundance in the corresponding cluster/geometry, while exemplar colour is proportional to overrepresentation. Asx and ST turns each show characteristic geometries at *N”’*, as they do at *NCap*.
Additional file 3:
**Asx and ST motifs at** ***N’*** **and** ***N”.*** Asx and ST motifs that occur at *N’* and *N”* are mapped, with example structures and global and peak-cluster (*) motif data (Abundance/Overrepresentation/Pvalue). Exemplar width is proportional to motif abundance in the corresponding cluster/geometry, while exemplar colour is proportional to overrepresentation. Asx and ST motifs show characteristic geometries at *N’* and *N”*, as they do at *NCap* and *N”’*.
Additional file 4:
**First-order polar motifs at** ***N3*** **.** First-order polar motifs at *N3*, with example structures and global and peak-cluster (*) motif data (Abundance/Overrepresentation/Pvalue). Exemplar width is proportional to motif abundance in the corresponding cluster/geometry, while exemplar colour is proportional to overrepresentation. Polar amino acids at *N3* are well-positioned to interact with the loop, and they play important roles which include serving as components of capping boxes and big boxes.
Additional file 5:
**First-order proline motif at** ***N”’.*** The first-order proline motif at *N”’* is mapped, with example structure and global and peak-cluster (*) motif data (Abundance/Overrepresentation/Pvalue). Exemplar width is proportional to motif abundance in the corresponding cluster/geometry, while exemplar colour is proportional to overrepresentation. This motif favours a loop geometry that brings the backbone towards (−y) and supports a beta-turn towards (-x, -y).
Additional file 6:
***N”*** **carbonyl boxes.** The pair motifs *NCap*T-*N3*N and *NCap*T-*N3*R are mapped, with example structures and global and peak-cluster (*) motif data (Abundance/Overrepresentation/Pvalue). Exemplar width is proportional to motif abundance in the corresponding cluster/geometry, while exemplar colour is proportional to overrepresentation. These motifs can form “*N”* carbonyl boxes”, in which the ST N-cap motif is joined by an H-bond between the *N3*SC and the *N”*MCC.
Additional file 7:
**Capping box alternatives.** Examples of motifs that have polar residues at *NCap* and *N3* but tend to form structures other than the capping box are mapped, with example structures and global and peak-cluster (*) motif data (Abundance/Overrepresentation/Pvalue). Exemplar width is proportional to motif abundance in the corresponding cluster/geometry, while exemplar colour is proportional to overrepresentation. The motifs *NCap*D-*N3*T and *NCap*D-*N3*S commonly form SC/SC H-bonds rather than the reciprocal SC/MC H-bonds of the capping box.
Additional file 8:
**Miscellaneous polar SC/SC motifs at the N-terminus.** A selection of pair (A) and triplet (B) polar motifs at the N-terminus is shown, with example structures and global and peak-cluster (*) motif data (Abundance/Overrepresentation/Pvalue). Exemplar width is proportional to motif abundance in the corresponding cluster/geometry, while exemplar colour is proportional to overrepresentation. These motifs are associated with salt bridges, H-bonds and electrostatic interactions.
Additional file 9:
**Polar pairs at (** ***N”*** **,** ***N4*** **).** The polar pair motifs *N”*T-*N4*T and *N”*T-*N4*N are mapped, with example structures and global and peak-cluster (*) motif data (Abundance/Overrepresentation/Pvalue). Exemplar width is proportional to motif abundance in the corresponding cluster/geometry, while exemplar colour is proportional to overrepresentation. These motifs support particular geometries with SC/SC H-bonds.
Additional file 10:
**Particular hydrophobic pair motifs at the N-terminus.** Particular instances of hydrophobic pair motifs, at (*N’*, *N4*) (A) and (*N”*, *N4*) (B) are shown, with example structures and global and peak-cluster (*) motif data (Abundance/Overrepresentation/Pvalue). Exemplar width is proportional to motif abundance in the corresponding cluster/geometry, while exemplar colour is proportional to overrepresentation. These motifs include particular examples of the general hydrophobic pairs from Fig. 8. Motifs occurring at the position pair (*N”*, *N4*) show prominent peaks in the geometries in which the loop turns towards (−z) at *N’*, bringing the SCs at *N”* and *N4* close for hydrophobic interaction.
Additional file 11:
**Higher-order hydrophobic motifs at the N-terminus.** Triplet (A) and quadruplet (B) motifs are mapped, with example structures and global and peak-cluster (*) motif data (Abundance/Overrepresentation/Pvalue). Exemplar width is proportional to motif abundance in the corresponding cluster/geometry, while exemplar colour is proportional to overrepresentation. Three triplet motifs that combine Asx/ST N-caps with particular pairs of hydrophobic amino acids at (*N’*, *N4*) are mapped, along with two quadruplet motifs that combine capping boxes with general hydrophobic pairs.
Additional file 12:
**Aromatic pair motifs at the N-terminus.** Three aromatic pair motifs at the N-terminus are mapped, with example structures and global and peak-cluster (*) motif data (Abundance/Overrepresentation/Pvalue). Exemplar width is proportional to motif abundance in the corresponding cluster/geometry, while exemplar colour is proportional to overrepresentation. All three motifs can form perpendicular pi stacking interactions on the (−x) side.
Additional file 13:
**Aromatic-proline pairs at the N-terminus.** Two pair motifs that represent aromatic-proline interactions at the N-terminus are mapped, with example structures and global and peak-cluster (*) motif data (Abundance/Overrepresentation/Pvalue). Exemplar width is proportional to motif abundance in the corresponding cluster/geometry, while exemplar colour is proportional to overrepresentation.
Additional file 14:
**Triplet motifs with glycine at the N-terminus.** Three triplet motifs that incorporate glycine at the N-terminus are mapped, with example structures and global and peak-cluster (*) motif data (Abundance/Overrepresentation/Pvalue). Exemplar width is proportional to motif abundance in the corresponding cluster/geometry, while exemplar colour is proportional to overrepresentation. In these motifs, glycine contributes conformational flexibility or space for packing.
Additional file 15:
**Beta-turn motifs at the N-terminus.** Six pair motifs that favour beta-turn geometries are mapped, with example structures and global and peak-cluster (*) motif data (Abundance/Overrepresentation/Pvalue). Exemplar width is proportional to motif abundance in the corresponding cluster/geometry, while exemplar colour is proportional to overrepresentation.
Additional file 16:
**Proline motifs at the C-terminus.** A pair and two triplet motifs that include proline are mapped, with example structures and global and peak-cluster (*) motif data (Abundance/Overrepresentation/Pvalue). Exemplar width is proportional to motif abundance in the corresponding cluster/geometry, while exemplar colour is proportional to overrepresentation. Asp and Pro can form DP motifs near the C-terminus, and Ser or Thr at *C”’* can join to form triplet motifs.
Additional file 17:
**Polar pairs at (** ***C3*** **,** ***C”*** **).** Three polar pairs at (*C3*, *C”*) are mapped, with example structures and global and peak-cluster (*) motif data (Abundance/Overrepresentation/Pvalue). Exemplar width is proportional to motif abundance in the corresponding cluster/geometry, while exemplar colour is proportional to overrepresentation. These motifs exhibit electrostatic interactions and salt bridges that favour Schellman loops.
Additional file 18:
**Electrostatic triplet at the C-terminus.** The electrostatic triplet *C4*E-*CCap*R-*C”*E is mapped, with an example structure and global and peak-cluster (*) motif data (Abundance/Overrepresentation/Pvalue). Exemplar width is proportional to motif abundance in the corresponding cluster/geometry, while exemplar colour is proportional to overrepresentation. This motif favours a Schellman loop geometry.
Additional file 19:
**First-order polar motifs with {Asp, Asn, Ser, Thr} at** ***CCap*** **.** First-order polar motifs at *CCap* are mapped, with example structures and global and peak-cluster (*) motif data (Abundance/Overrepresentation/Pvalue). Exemplar width is proportional to motif abundance in the corresponding cluster/geometry, while exemplar colour is proportional to overrepresentation. Although the polar amino acids {Asp, Asn, Ser, Thr} are much less important at *CCap* than they are at *NCap*, they nevertheless play structural roles by capping the helix or interacting with the loop.
Additional file 20:
**First-order polar motifs at** ***C1*** **and** ***C’*** **.** First-order polar motifs at *C1* and *C’* are mapped, with example structures and global and peak-cluster (*) motif data (Abundance/Overrepresentation/Pvalue). Exemplar width is proportional to motif abundance in the corresponding cluster/geometry, while exemplar colour is proportional to overrepresentation. Polar motifs at these positions may play a hydrophilic role (*C1*E), or cap the helix {*C1*N, *C’*R, *C’*S}.
Additional file 21:
**First-order aromatic motifs at** ***CCap*** **.** The first-order aromatic motifs *CCap*F and *CCap*Y are mapped, with example structures and global and peak-cluster (*) motif data (Abundance/Overrepresentation/Pvalue). Exemplar width is proportional to motif abundance in the corresponding cluster/geometry, while exemplar colour is proportional to overrepresentation. The cyclic SCs of these motifs can pack between hydrophobic components in the helix and loop, supporting beta-turns secured by *C”’*MCA → *CCap*MCC H-bonds.

## Abbreviations

SC: Side chain
MC: Main chain
MCA: Main chain amide
MCC: Main chain carbonyl
