# Supplementary material for: Mapping side chain interactions at protein helix termini
Source: BMC Bioinformatics. 2015 Jul 25;16:231. doi: 10.1186/s12859-015-0671-4 (PMC4515027; doi:10.1186/s12859-015-0671-4)
Supplement: Additional file 1: — Motif example file. This file contains references to example structures from the PDB for each sequence motif given in the results. Up to 20 examples are provided for each motif. Complete lists of all examples for each motif are available from the author. [file 12859_2015_671_MOESM1_ESM.doc]

Motif Example File

This file contains examples from the PDB for each sequence motif given in the results section. Examples are taken from clusters in which the motif is highly overrepresented, and up to 20 examples are given for each motif. It should be noted that not all instances of a sequence motif are accompanied by structural motifs; examples of sequence motifs should be examined in a structure browser to determine whether the corresponding structural motif is present. Each example is specified in the format:

<PDBID>$<ChainID>$<StartPosition>$<EndPosition> <Sequence>

For example, an instance of the capping box sequence motif *NCap*T-*N3*E is specified:

3AEY$B$264$271 EAVTDEEI

Here the motif is in PDBID 3AEY, chain B, between positions 264 and 271, and the complete sequence at the N-terminus from N’’’ to N4 is EAVTDEEI.

Motif examples are organized by figure number and labelled with both a shorthand text specification (for example *NCap*T-*N3*E for the motif above) and a listing of the motif within the terminal sequence using wildcards (xxxTxxEx for the motif above).

Motif examples:

Fig. 4

NCapS - xxxSxxxx:

3HF5$D$12$19 ESMSHEQF

3MD7$A$218$225 SHFSLGEA

3GO5$A$254$261 FGISKGQF

4EQB$A$87$94 AIPSEYMI

3RPW$A$108$115 SKLSESAH

3OLQ$A$50$57 TLLSPDER

2IF6$B$93$100 GGLSVEQQ

4F55$A$140$147 NGYSQNDI

3I3F$C$91$98 TSLSDSEE

3UJC$A$131$138 LALSLENK

3GF3$A$60$67 GQLSAMQR

3MBC$B$84$91 ISASVPQL

3VJZ$B$39$46 SCLSDDQH

1TKE$A$137$144 KKVSWHEA

1TJV$D$408$415 NKLSLEDL

4AK5$B$48$55 NKLSAAMK

3SUV$A$324$331 NATSAADY

2J9O$D$165$172 NTTSPRDL

2R8U$B$13$20 DNLSRHDM

2HSJ$A$192$199 LHLSIAGY

NCapT – xxxTxxxx

4MUR$A$171$178 QKWTLEEY

3F0H$A$111$118 KKLTKEKL

2X9X$A$279$286 LKLTDAGL

3TT9$A$349$356 MEMTLERA

3VX0$A$38$45 CGGTWQGI

3SMZ$A$68$75 GDVTNQEV

2V3G$A$124$131 RVNTNETY

3LUL$B$219$226 ISLTKKRI

1SU8$A$70$77 CGATAEVI

3CEX$B$137$144 PPVTRQVR

3OI8$B$167$174 GLVTFEDI

3D3Y$A$178$185 AEETAASL

3H63$C$184$191 GKVTISFM

4IIB$B$458$465 YLVTPEQA

3PC3$A$493$500 ALATKLDV

2RFA$A$116$123 EGQTALHI

1UZ3$B$6$13 VWPTLLDL

3HL8$A$67$74 TGITPQEA

3HX3$A$169$176 QEITFDEI

4BVQ$B$164$171 PLLTPAKI

NCapD – xxxDxxxx

1T3Y$A$3$10 TKIDKEAC

4HLJ$A$662$669 QGVDHKQV

2ZAC$A$261$268 RSVDFVGK

1U2K$A$562$569 DQTDIEMF

1VJ0$A$154$161 EKDDLDVL

4C2E$B$68$75 EKVDREKL

2P02$A$97$104 AAVDYQKV

4DZI$D$316$323 FPEDPVEQ

2NAC$B$125$132 DHVDLQSA

3DSK$B$205$212 GKADFGGS

2PQ7$A$75$82 TGVDHAES

3D02$A$265$272 ITYDPATA

4AW7$A$122$129 GGIDVQAA

3H74$A$153$160 VTPDLEVI

3BNJ$A$72$79 NITDMLKE

1Y0P$A$454$461 EGIDGKAL

3LLU$A$126$133 PTFDYEMI

4NI3$B$147$154 PKLDLVKL

4KYQ$A$136$143 WGIDLDSI

NCapN – xxxNxxxx

1JMW$A$142$149 DNGNMDAY

3C9F$B$82$89 HDGNGLSD

5CSM$A$191$198 NITNSAVE

3QHB$B$145$152 EQGNMEVA

4FW1$B$146$153 IPGNSQGQ

3CKC$A$272$279 NDENENAM

4A4A$A$745$752 NNGNGEKF

2OF3$A$698$705 FETNPAAL

1Y8A$A$23$30 VFNNARFF

2GQW$A$367$374 CVNNARDF

2VQ2$A$66$73 DPKNELAW

3ME7$B$146$153 VNYNYLEF

3FXH$A$91$98 LKSNALLL

4F3M$B$110$117 FDRNEGKH

3TP4$B$223$230 FGGNRDVY

3FTB$A$228$235 ITNNKEIA

2OB0$C$114$121 QISNESAI

2GJU$A$10$17 IAGNFPAL

1VMG$A$44$51 LSNNLDSI

Fig. 5

N1P – xxxxPxxx

1XTE$A$74$81 DNFDPDFI

1CSH$A$341$348 LPSDPMFK

2F2H$F$388$395 DFTNPDAC

1R3S$A$40$47 GRYLPEFR

4FYU$C$36$43 AHWCPPCR

2WAN$A$593$600 ATEHPMAQ

1QUS$A$245$252 NLWDPVDA

3OHS$X$1158$1165 LTHVPRAV

1H41$B$258$265 DPLDPRVQ

4DWO$A$119$126 NSLDPYVQ

3LX4$B$127$134 TSCCPGWI

4KWD$D$113$120 DRSIPVEY

1U60$D$27$34 ADYIPFLA

3L4Y$A$414$421 DYTNPNCA

1QSA$A$65$72 NPTLPPAR

4IUJ$A$299$306 GEGIPLYD

3RPP$C$13$20 DVLSPYSW

3SZ7$A$144$151 APANPIYL

3KUV$B$27$34 YPESPEFA

2QF7$A$93$100 LSESPEFV

NCapP – xxxPxxxx

1JHG$A$90$97 KAAPVELR

3B0G$A$69$76 AKIPIEEI

3K13$C$603$610 SDIPADTL

3A2Q$A$243$250 ASRPYAQG

2Q35$A$207$214 AKSPRLSL

3V5C$A$176$183 RHMPLWEG

3ELF$A$133$140 SAVPIDEN

2EA7$A$375$382 SEIPTEVL

3A09$A$370$377 AAQPVNNQ

2C61$B$213$220 ADDPAVER

4H8E$A$82$89 WSRPESEV

1W9H$A$385$392 RNLPVTVN

3N75$E$127$134 TILPPLTK

4E3X$A$117$124 DLKPMADR

3MT0$A$218$225 EEGPADVL

2JHF$B$246$253 YKKPIQEV

3NN1$E$26$33 WDLPGESR

2JAE$B$291$298 CTIPPHLV

2XPW$A$164$171 ENLPPLLR

3NEU$A$7$14 ADKPIYSQ

N’P – xxPxxxxx

2DE3$B$202$209 LFPDVARG

3RQZ$A$38$45 YGPRPREC

2YCD$A$138$145 NEPWHEAR

2YZV$A$143$150 NHPDLLSL

3W42$B$159$166 NVPDIRSH

2GJU$A$46$53 LFPYPREV

1EU8$A$366$373 IVPYYPQL

1HDH$A$331$338 FGPDLLGF

1B93$A$95$102 AVPHDPDV

3CSG$A$269$276 ASPNKELA

3RAY$A$60$67 GIPDRAAL

3FO3$B$96$103 RSPMFDKL

3ATS$A$178$185 SIPNAQNT

3S2J$A$72$79 DLPGAVTA

3SH4$A$140$147 GAPDVATL

3VK5$B$255$262 EQPDWRSA

1F0I$A$246$253 CMPTMHKD

2X7M$A$11$18 RIPMLSSI

1VYR$A$328$335 ANPDLVAR

4KWD$D$19$26 CHPLVEEV

N’’P – xPxxxxxx

2VQ2$A$240$247 FPYSEELQ

2CUL$A$51$58 PPGSLLER

1PXZ$B$54$61 TPGTLRYG

1ODM$A$111$118 TPDHPRIQ

3JTM$A$78$85 GPDCELEK

3QH4$A$290$297 LPEWTTSQ

3UV4$B$1605$1612 GPESQYTK

3EUA$A$109$116 KPESPLAQ

3PB6$X$106$113 TPGSPGNL

2R6V$A$46$53 APKRTTHK

1G8F$A$136$143 DPEHPAIS

4J3M$A$959$966 SPDDKEFQ

1M3S$B$114$121 NPESSIGK

3SG0$A$327$334 KPGTPEFR

2IZZ$A$233$240 SPGGATIH

2BKX$B$76$83 DPNSYHFY

1M0W$A$81$88 QPDSYLHK

3UPL$B$420$427 QPGSRIAE

3M07$A$473$480 DPNAPETF

3N3M$A$109$116 IPYGSVGI

Fig. 6

NCapT-N3Q – xxxTxxQx

2DBN$A$72$79 GHVTAEQR

2YH5$A$300$307 HTLTQSQN

1M15$A$138$145 PCLTAEQY

4GD5$B$122$129 KDLTLVQI

4GZK$A$159$166 ERMTARQV

3RG9$B$107$114 RSATKEQL

1RXQ$A$128$135 RTLTDQQF

3A9S$A$254$261 FKRTEEQK

2BEK$A$218$225 FGKTIAQH

3A21$B$289$296 DGFTAAQN

3CP7$B$101$108 GGTTVQQQ

3PMO$A$5$12 LSYTLGQL

2IAB$B$5$12 PARTAKQR

3NJD$B$202$209 DCITGAQA

4ADM$D$192$199 VPVTLGQE

3O0Y$A$517$524 KRWTREQA

2VXN$A$176$183 KVATPEQA

3N3R$B$473$480 SGLTVSQL

2JE8$A$55$62 VPGTVHQD

2BNM$A$49$56 GELTLTQL

NCapT-N3E – xxxTxxEx

3B9O$B$408$415 EEGTYREK

4H5I$B$50$57 TKDTEKEQ

3FP3$A$593$600 LARTMDEK

2WGK$B$261$268 CADTDKEA

2A33$B$177$184 SAPTAKEL

1OPK$A$210$217 RFNTLAEL

4EWE$A$257$264 VMNTNEEI

3VVY$D$47$54 YFATKDEL

4AMM$A$722$729 VAGTRPEL

2SHP$B$70$77 KFATLAEL

1MUN$A$104$111 FPETFEEV

2G50$H$21$28 MADTFLEH

2BW4$A$195$202 KYETPGEA

1VKI$B$30$37 PVFTVAES

1O0W$B$216$223 KGRTKKEA

3IRS$C$98$105 EAATRKEA

2X1D$A$6$13 CQGTPFEI

3BPT$A$273$280 SANTVEEI

1EU8$A$142$149 PPETWQEL

2IZR$A$253$260 KADTLKER

NCapS-N3E – xxxSxxEx

2HQ9$B$4$11 RTLSALEC

3GH1$D$14$21 DLLSQLEV

4FNV$A$99$106 VTISEAEQ

3LFJ$B$121$128 VSLSEDEV

3HKW$C$81$88 NLLSVEEA

4FGQ$B$122$129 YWKSPVEF

4KOP$A$113$120 FALSPTEV

2XIG$A$41$48 THLSPEEI

3GWZ$A$312$319 AERSESEF

3ARL$A$3$10 VGLSDSEE

3F0I$B$36$43 TSPSVEEL

2Y24$A$175$182 CEWSGDEF

4JHC$A$128$135 RHLSEAEI

2DY1$A$271$278 ALPSPTER

1JND$A$314$321 GLLSYAEI

4IQY$B$39$46 SILSWKEE

2QA1$A$401$408 RETSSTEL

2QP2$A$286$293 DQPSAEEI

2DC3$B$17$24 EELSEAER

1YHT$A$255$262 MRVSLPEL

NCapS-N3Q – xxxSxxQx

3M0J$A$260$267 GFRSKEQA

3AHC$A$475$482 EQLSEHQC

3N29$B$101$108 VFNSLAQF

3K3T$A$239$246 AYDSYEQA

2Y71$A$51$58 QSDSEAQL

4C6I$A$1567$1574 RLDSVVQW

3PZS$A$82$89 YIGSPEQG

1XQA$B$66$73 PQESEEQV

3VC1$A$126$133 VTLSAAQA

3F4M$A$49$56 YTHSRPQA

3HF5$D$68$75 WFASEEQY

3QBM$A$46$53 HFESKEQL

1U7I$B$83$90 DCESNAQI

2Y53$B$337$344 SLVSREQY

3JWA$A$38$45 VFDSAEQG

2P8J$A$51$58 IEISDLQL

4A57$D$470$477 GFESVDQV

1Y57$A$219$226 QFNSLQQL

1JQE$A$88$95 VEPSAEQI

3TO7$A$204$211 YFGSKKQY

Fig. 7

N’D-N4R – xxDxxxxR

3M9Q$B$73$80 LKDTEENR

3NVS$A$44$51 LLDSDDIR

1V0L$A$138$145 RRDSNLQR

3PG6$A$664$671 LPDNKEGR

2WMF$A$99$106 PRDTKELR

3NW4$A$17$24 PKDTPELR

2XJ4$A$113$120 GGDSAITR

3MG1$B$89$96 RADTPLCR

N’K-N4E – xxKxxxxE

3E9K$A$410$417 PNKDVFQE

1ILK$A$115$122 ENKSKAVE

4K08$A$125$132 NGKLLFQE

4FUS$A$274$281 QGKDPEQE

3S44$A$153$160 ENKDISAE

2WDC$A$350$357 LPKAEDVE

2YVT$A$42$49 ILKNEALE

3MK1$A$229$236 DGKNLVQE

N’R-N4E – xxRxxxxE

1RHS$A$39$46 GTREARKE

4J27$A$156$163 YKRDILGE

2X0Q$A$354$361 GFRNYYYE

3E2D$A$186$193 DDRNLLTE

2X0Q$A$404$411 QGRALLPE

3EPR$A$20$27 KSRIPAGE

2HNG$A$20$27 DARNFEWE

1N97$B$124$131 EERDLDHE

3RRX$A$15$22 LKRDPAVE

1H12$A$16$23 TYRNLAQE

Fig. 8

N’h-N4h – xxhxxxxh

3CUZ$A$472$479 KPVTKALF

3U9G$A$111$118 DVLSEQNF

4MO2$A$93$100 LPFNMNTF

2RAS$A$29$36 AGLTLSEL

2Z6O$A$130$137 PKFGLAHL

2VG2$D$220$227 ERITESTI

3FO8$D$348$355 AEVTAGDL

1YLK$A$61$68 CVVTDDVI

4IRG$A$341$348 PNMNYDKL

4DMG$B$71$78 GPLNRAFF

3U3L$C$134$141 HNITREII

2YB1$A$130$137 EMISRTHF

1OPK$A$352$359 QEVSAVVL

4KZP$D$231$238 DPLSTDHV

2WOL$A$113$120 TPITSDDV

3OCM$A$440$447 GLVTPIDV

2IKB$D$107$114 GVIGAVSL

2BJF$A$245$252 DSIDLIEF

3L60$A$214$221 PEITPFAL

3HL1$B$107$114 GPLDLTRV

N’’h-N4h – xhxxxxxh

2H0U$A$127$134 KLNSERSL

2QIB$B$53$60 YFPGKLSL

2IRU$B$95$102 IIDSATGL

3KSX$A$64$71 EFPAGPQL

4GP7$B$154$161 ILNSPEEV

4A29$A$159$166 LINDENDL

4FOJ$A$654$661 FVADAQSM

4N4U$B$152$159 KLESLADI

1ZSW$A$84$91 LVPSEDSL

1JTA$A$37$44 IVTNISEF

2YW3$F$110$117 GVLTPTEV

1G1T$A$28$35 AIQNKEEI

2C0A$C$114$121 GISTVSEL

2PYW$A$405$412 KFKSIGEV

1KLL$A$78$85 EFPDTASV

4LGJ$A$66$73 RLIDQHTV

3KZX$A$193$200 SFKNFYDI

3B9O$B$352$359 SVGTPKKV

3H8V$B$150$157 NITTVENF

1VL2$D$151$158 KFKGRTDL

N’’’h-N4h – hxxxxxxh

3A02$A$122$129 IGLTEARI

2XSW$A$482$489 LSGGRTVV

4AVA$A$311$318 LSLGREMV

3GEU$A$30$37 VNIKKASL

4E2V$A$233$240 VGEGQDEM

4C3S$A$321$328 VGKDARTL

2V25$B$171$178 FSVDKSIL

2X5N$A$118$125 IVEDEKNL

1WY3$A$51$58 FGMTRSAF

1G5H$A$395$402 VHSSLEQL

1N3L$A$234$241 LLDRKEDV

3OCM$A$349$356 IDDDAATI

3OMT$B$28$35 LDKNKTTV

3CEC$A$40$47 LGVSNQTI

4A1I$A$19$26 FRISTAAL

3II2$A$135$142 IAEDDIQM

3D1P$A$72$79 FALDPLEF

3N0U$C$68$75 VHLPLEEL

3OP9$A$58$65 FHLSIDEL

1NTE$A$247$254 IGLKDSQI

Fig. 9

NCapC-N3C – xxxCxxCx

2OWA$B$56$63 VFICLNCS

2XOC$A$598$605 TVLCYCCG

3LJU$X$38$45 VFICLSCS

3ZYQ$A$209$216 VRVCEPCY

4IC3$B$468$475 LVTCKQCA

1E7L$A$55$62 GLLCNLCD

2IQJ$B$45$52 VFICIRCA

3T7L$A$794$801 ARVCVVCY

2P57$A$43$50 VFLCIDCS

2ORW$B$170$177 IAVCRDCY

N1C-N4C – xxxxCxxC

3FO3$B$375$382 NGVACADC

2A3M$A$75$82 TVATCMSC

1OFW$B$93$100 TPVSCVSC

1J0P$A$75$82 KFKSCVGC

3BNJ$A$291$298 KGVSCADC

N’’G-NCapG-N1K-N2T – xGxGKTxx

2GXQ$A$47$54 TGTGKTLA

1JJV$A$11$18 IGSGKTTI

3VKW$A$835$842 PGCGKTKE

4KO8$B$247$254 PGTGKTLI

2WOJ$A$27$34 GGVGKTTS

3DO6$A$55$62 AGEGKTTT

4N0N$A$160$167 PGSGKTFH

1LV7$A$197$204 PGTGKTLL

N’’’{S,T}-N’’H-NCapH-N2D-N3H – {S,T}HxHxDHx

2CFU$A$168$175 SHAHADHF

3DHA$A$107$114 SHLHFDHA

1WRA$B$109$116 THTHSDHI

3M8T$A$100$107 THAHLDHT

3MD7$A$85$92 THPHADHI

1ZKP$A$58$65 SHYHHDHV

2P18$A$75$82 THKHWDHS

Fig. 10

C’G – xxxxxGxxx

2B3F$A$151$159 TLKQKGLEA

3LSN$A$55$63 ACEALGGKP

3CCG$A$65$73 ICTNEGYEL

2FYF$A$329$337 ILRANGIVD

3T9W$A$280$288 NHSDMGMAG

2FNU$B$304$312 SLHKRGILA

3V75$A$85$93 EARAAGALV

2PUZ$B$110$118 EIAKAGGGI

3BKB$A$771$779 EFVEKGGRL

1K7C$A$120$128 LFTAKGAKV

4H7P$B$21$29 PLIARGALL

3VGL$A$220$228 EAARQGDPV

1LUA$C$22$30 VGYDGGADH

3U0V$A$107$115 EEVKSGIKK

2NZL$A$246$254 EAVKHGLNG

1QBA$A$255$263 RFALLGVPV

3CWN$B$118$126 LYNDAGISN

4BEU$A$107$115 AVRESGFKG

3VKJ$D$206$214 LLYSYGIKN

2Z2Z$A$250$258 QAYNAGIVI

C’’G – xxxxxxGxx

2VFK$A$160$168 VEEILEGKH

3H8G$E$35$43 VDLASEGAI

3MBC$B$351$359 AMIRTSGHM

1EAQ$B$54$62 VLADHPGEL

4GIM$C$277$285 VAELTGGDS

3CKM$A$503$511 LAKSTGGEY

4N4U$B$50$58 IEKNTGGEV

1KWG$A$300$308 YRGVGRGRF

1IZC$A$88$96 AQHHSEGRS

3M73$A$215$223 YLSINHGEV

1YCD$B$198$206 YLKAQNGNK

1DCS$A$230$238 ATLVTGGQV

4KXV$A$548$556 SARATKGRI

1Y57$A$453$461 TELTTKGRV

2EX4$B$27$35 VDGMLGGYG

4GB7$A$312$320 QLLLNNGEY

3L3B$B$68$76 SARIARGSV

1M15$A$86$94 IDDYHGGFK

1NZJ$A$107$115 RIQSIGGIY

3U9G$A$13$21 ILCAHGGRM

CCapG – xxxxGxxxx

3EO7$A$78$86 FRSYGLTAR

2Q1S$A$253$261 CAADGTPGG

3EDF$A$513$521 VIHNGRLMH

3VQT$D$232$240 LEEAGTPFD

3OWA$D$123$131 IVLFGNEEQ

3E5U$C$161$169 CSSQGKRVG

3GVO$A$783$791 FFEFGSLDQ

1DCS$A$54$62 FFEHGSEAE

3ZO9$A$489$497 AFAVGTFRE

3ZUZ$A$227$235 THKYGNEED

2II1$A$264$272 LSGAGDLYV

2VQ3$B$153$161 TLQAGPRDG

2P51$A$136$144 HQEVGIEPA

3BMV$A$403$411 AIAYGTTQQ

4C6A$A$109$117 RGDFGVDVG

2HSB$A$89$97 ESDYGIYKE

3NRR$B$238$246 VLSHGVLKP

1Y0B$A$8$16 IEEEGVVLS

3N0U$C$29$37 LGEEGTEED

3GOH$A$253$261 LHDFGDRQD

Fig. 11

C’P – xxxxxPxxx

4BOL$B$153$161 HSDIAPGRK

4KG7$A$354$362 VRSKFPDLT

2I7D$B$26$34 FRRRFPEEP

2NT0$D$327$335 THRLFPNTM

3E3M$D$242$250 ILQEYPDTD

2WMF$A$394$402 ISSIFPNAK

1KB0$A$269$277 HKVRSPKGG

3B4U$B$157$165 LKAAFPGIV

2QTZ$A$562$570 LQEQHPDGN

1US3$A$300$308 MSYMQPTEG

4JOQ$B$58$66 LQEVYPQLD

2PE4$A$155$163 VQAQHPDWP

2WGP$B$141$149 VKARRPVIR

2YHG$A$691$699 LAKNFPNLI

3HJR$A$349$357 LMSAYPDLS

1GWU$A$87$95 VESACPRTV

2VHK$A$173$181 FKRLCPDAF

3BD1$A$55$63 CRELRPDVF

3QGU$A$62$70 HQEKNPDAK

2VQ2$A$96$104 ALSIKPDSA

C’’P – xxxxxxPxx

4DMG$B$208$216 FEAMVRPGE

3RLO$A$667$675 RSASVTPKI

2JH1$A$123$131 VQRFCSPYQ

3CZP$A$91$99 FWRRLPPKG

1YF9$C$97$105 INQTWTPMY

3OP7$A$146$154 LRQLIRPTT

3A09$A$114$122 QRAITAPDG

4JND$A$224$232 VRKSRDPSD

2Q7E$A$198$206 LEVLLNPKD

3GDH$A$668$676 GWFSVTPEK

2WUU$A$98$106 QWALCPPSY

2CXX$C$156$164 IDKVFIPIS

3LJK$A$449$457 AAHKVIPGN

3OCJ$A$109$117 LQRHLRPGC

3O02$B$61$69 TLQSTPPIS

3OCJ$A$70$78 RLVTHQPGS

3FED$A$221$229 PADYFAPEV

1H16$A$148$156 VFDVYTPDI

2HEK$B$342$350 LIASLKPIK

3UCJ$B$111$119 AGLVWHPKT

C’’’P – xxxxxxxPx

1QMY$C$59$67 LFRYVEEPF

4GMF$A$48$56 LAHAFGIPL

4EQS$A$198$206 ELDKREIPY

3KUU$A$34$42 VLTTLNVPF

3H4T$A$42$50 RCAEVGVPM

3B6E$A$349$357 KKKKASEPG

1US3$A$452$460 DFQARSIPI

4FE3$A$152$160 KLQQHGIPV

2WTE$B$52$60 QISRLNYPP

2APJ$D$184$192 LRHDLNLPS

3BOE$A$417$425 TVEMLGGPK

1LB3$A$120$128 LGSARADPH

3ZIY$A$191$199 EKAIDERPS

3PFE$A$143$151 ALEQQGLPY

3M3P$A$23$31 FLAGEHIPF

3GA7$A$274$282 TLQAHQQPC

3UR8$B$188$196 FLARHNLPL

1X9I$B$101$109 YAKRRRIPA

2VCH$A$126$134 VAVEFHVPP

2GSO$B$401$409 MSRLLGIPA

Fig. 12

CCapD-C’’R – xxxxDxRxx

2OU5$B$71$79 HIRRDARVA

3A6R$A$57$65 NVARDERVL

2ARZ$B$60$68 NLQADPRCS

1WC2$A$160$168 EHNHDHRTP

CCapE-C’’R – xxxxExRxx

1WTJ$B$197$205 IAAREGRLL

1GKM$A$44$52 QIIAEDRTA

2YWI$B$168$176 DALLEGRPV

4EWF$A$169$177 QALLEGRAA

3AEY$B$299$307 KLLREGRLE

1E58$A$14$22 QWNKENRFT

4N7Q$A$531$539 KARFEFRWM

CCapD-C’’S – xxxxDxSxx

1QZR$B$74$82 NKVRDPSMK

2DY1$A$405$413 LLEEDPSLK

4GCO$A$237$245 ALQVDPSNE

4MLP$D$437$445 GRRTDPSGD

3HYN$A$39$47 WKGEDNSFP

1PX4$D$475$483 IKSVDPSRP

1V0L$A$158$166 ARAADPSAK

1VL5$D$162$170 EKERDYSHH

CCapD-C’’T – xxxxDxTxx

3CMG$A$448$456 AKQEDPTRP

1EOK$A$44$52 WSLQDTTKL

4N49$A$516$524 AFVQDTTLS

4J7A$D$264$272 VKPYDPTGE

4BBY$A$643$651 KDYVDPTNI

2GQW$A$303$311 RHLVDPTAP

1XNF$B$122$130 VLELDPTYN

1QW9$B$201$209 MKWVDPTIE

Fig. 13

C3Y-C’’F – xYxxxxFxx

2OB0$C$123$131 FYRKFGFEI

4F9D$B$483$491 AYQQAGFSG

2QEC$A$170$178 LYNRLGFVP

1P0H$A$293$301 TYQSLGFTT

3GY9$A$121$129 FYQGLGFQL

2QHS$A$62$70 WYRENGFEL

3FNC$A$130$138 FYKAKGFVQ

3OWC$B$143$151 LYRRAGFRE

2VEZ$A$173$181 FYIKCGFKR

4BMH$A$233$241 FYDRLGFHE

2DXQ$B$132$140 FYESCGFVQ

1IOM$A$318$326 VYSDLGFSL

2EHZ$A$166$174 FYSLLGFRG

2BUE$A$163$171 CYEKAGFER

3PP9$A$148$156 FYEKCGFVI

2JDC$A$117$125 YYKKLGFSE

3FYN$A$130$138 VYSRAGFEE

4MI4$C$133$141 LYEECGFVE

3EC4$B$206$214 LYESLGFRA

3ZJ0$A$181$189 FYEHRGFRE

C2Y-C’’’R – xxYxxxxRx

3SZY$A$116$124 AFYDAGARV

2WGK$B$115$123 ASYMHGIRN

3Q1X$A$141$149 VLYECGERY

3DU1$X$8$16 RHYAAGKRN

3V5C$A$158$166 QGYAKGQRH

3CTP$B$166$174 HLYEKGCRK

2WJE$A$28$36 ESYRQGVRT

1WMD$A$117$125 QAYSAGARI

3S2J$A$137$145 ALYALGVRY

4AAN$A$193$201 DKYLKGNRK

1ITU$A$139$147 ALYQLGMRY

2J8K$A$8$16 QLYAAGERD

2B0V$A$132$140 EDYHAGKRY

C1H-CCapF – xxxHFxxxx

2PYQ$D$102$110 LTKHFGKES

2A2C$A$118$126 IQEHFGLSN

3HH1$A$49$57 LLKHFGIEG

2Z84$A$107$115 CLEHFGGPQ

2IEA$B$837$845 LRHHFEVDA

2ZQ5$A$331$339 IYRHFGLTL

C4Y-CCapH – YxxxHxxxx

2QSA$A$103$111 YYLDHPDQR

4A8U$A$150$158 YLLAHSDAY

3MJO$B$8$16 YIANHTDPV

1Z2N$X$72$80 YEKDHPKVL

2HI0$B$99$107 YYADHCQIK

3ED4$D$262$270 YQKQHPDLF

3S44$A$123$131 YRFKHLDRV

3GA7$A$144$152 YFSQHADEY

3KQ5$A$296$304 YIDLHVNVP

2Q7D$A$84$92 YIDAHPETI

Additional file 2

N’’’S – Sxxxxxxx

3SLR$A$94$101 SLVNSDLQ

4I6R$B$31$38 SNLDRTYI

4DTH$A$296$303 SRYSESLS

4L9A$B$167$174 SHFSSQQF

3LSN$A$131$138 SDASAEIR

2W43$B$95$102 SNGSINEV

1VYK$A$94$101 SAKPKDEK

1ODZ$B$52$59 SQATMETR

4EO3$B$61$68 SRDSVEAL

3NRW$A$69$76 SDVSPATL

3LGD$A$457$464 STLLESEK

2ZHJ$A$216$223 SQKDIEQS

3ELF$A$255$262 SGSLKSEI

3QUF$B$316$323 SRASDKEK

3IC3$A$65$72 SEYTEDEA

3ON1$A$42$49 SDAGIHTK

3BPT$A$318$325 SSKTLQEV

1XSV$B$21$28 SLLTNKQR

2WLR$A$292$299 SIRSWPEF

3E58$B$109$116 SSSVKADI

N’’’T – Txxxxxxx

3AL2$A$1361$1368 TGINVQQR

2I3F$B$117$124 TDKSLEQC

1XIY$B$87$94 TNNDIYVL

4HDE$A$129$136 TGYSLEDI

3TGN$B$124$131 TQFTPNEQ

3IP0$A$62$69 TSLAPEEL

4HB9$A$259$266 TDFSAEAL

4ACI$B$41$48 TGKSRGAI

3SIG$A$72$79 TEETTLAA

3CH0$A$198$205 TKGTLKKQ

2CC0$B$70$77 TQLGQAQM

3VIS$B$148$155 TDASSAVR

1OFD$B$284$291 TGRSPLEA

1WR8$B$43$50 TGNTVQFA

1KGS$A$81$88 TALSDVEY

2DUL$A$364$371 TSAPYEVF

3QBC$B$63$70 TTLTVLQL

4EB0$A$144$151 TSSPSAVR

2I4L$C$383$390 TDQRAGAK

2ICX$A$19$26 TEMSESEK

N’’’D – Dxxxxxxx

1ZI8$A$74$81 DPQDERQR

2G1U$A$58$65 DAAEFETL

1XTE$A$74$81 DNFDPDFI

3TTC$A$229$236 DARNSNAV

2F2H$F$388$395 DFTNPDAC

2FBA$A$310$317 DVDNEYVL

3QAY$D$127$134 DKRLYILN

3AJR$B$47$54 DVSNRDEI

2QS9$B$102$109 DLGDENER

3SZ7$A$178$185 DPKYSKAW

1H41$B$258$265 DPLDPRVQ

4KWD$D$113$120 DRSIPVEY

3HV8$A$624$631 DLNQVENQ

2RA4$B$53$60 DPKEKWVQ

3L4Y$A$414$421 DYTNPNCA

3RPP$C$13$20 DVLSPYSW

4MPG$B$8$15 DLVSQPSR

1ZXT$D$54$61 DPSKNWVR

2QQ5$A$64$71 DSSQESEV

3MBC$B$485$492 DPERAHDR

N’’’N – Nxxxxxxx

3BV6$A$77$84 NTQHQMMR

2IS9$A$103$110 NLEDLATL

1W8S$J$154$161 NETAPEIV

2P35$B$132$139 NLQEPTHI

2A3N$A$126$133 NADSPLAQ

2P7I$B$142$149 NANAVSRQ

1I0S$B$47$54 NKENDTHN

1V0L$A$176$183 NWTWAKTQ

1M3S$B$114$121 NPESSIGK

3UFE$B$32$39 NKESVNYL

2C15$B$103$110 NPEGIAQR

1NRG$A$112$119 NFESRKGK

1W7C$A$189$196 NLNTTIMR

2YFO$A$418$425 NEDSDLYR

2F5X$C$270$277 NEANPEAL

3FXA$D$127$134 NPDSVIAK

3TG7$A$496$503 NPNTYDYM

4F2L$A$315$322 NHQDPLAV

3I48$B$198$205 NKGTPEFK

2W40$A$133$140 NTYFSAFK

Additional file 3

N’N – xxNxxxxx

3WC4$A$250$257 DSNGCLSW

3C5K$A$48$55 YINGHMLQ

2PUZ$B$92$99 GGNRAMEF

2YHG$A$569$576 TSNTAYVD

4GY7$A$666$673 SDNDNFRI

2I49$A$398$405 KVNREDLW

2FHF$A$537$544 KDNPQVQA

1JGT$A$277$284 TSNEFREA

2WGK$B$204$211 SYNSPSMR

4I66$A$190$197 VQNDLGGA

2DBN$A$111$118 DRNRFDEV

4E57$B$83$90 LTNGVPER

4N01$A$273$280 IRNDDDFQ

2VZS$A$583$590 FGNLKLFG

1I2K$A$141$148 HLNRLEQV

3OMT$B$6$13 IFNRLKSV

1GOI$A$409$416 NRNGDLLA

3ZIY$A$329$336 APNMSAVT

4EYS$A$91$98 FENDQLQK

3S2J$A$19$26 GHNDLPWA

N’’S – xSxxxxxx

3NMW$B$670$677 KSHSLTIV

4CA7$A$155$162 KSRDHEEL

2YG9$B$174$181 SSGDLALR

2QM8$B$31$38 ESRRADHR

2W1V$B$51$58 VSSIKSDN

4DV8$A$745$752 HSTDHAER

1E58$A$56$63 TSVLKRAI

3CWN$B$62$69 QSNDRAQQ

3AJ3$A$137$144 LSFSAEAA

2C1I$A$393$400 KSKNEASI

3SZA$B$366$373 FSSNDKVI

4IIB$B$712$719 NSTDLKAS

4BLP$A$43$50 SSTNAAAV

3K13$C$431$438 DSSKWEVI

3DB2$C$35$42 YSRTEDKR

3FVQ$B$195$202 VSHDREEA

1O98$A$407$414 HSGKLEPT

2Y7E$B$56$63 PSQRLDRF

1Z72$B$131$138 ASSDYAHL

2YCI$X$76$83 DSTNPDAI

N’’T – xTxxxxxx

3UPL$B$95$102 VTDDNDLI

2WW8$A$324$331 LTNDKNDI

3S2J$A$242$249 VTFVPKFV

2DH4$B$122$129 LTTKEPLY

3UFB$A$329$336 QTAETAML

3I0Z$A$287$294 GTTTDYTR

4DMV$A$37$44 TTNNNENK

1EYE$A$86$93 DTMRADVA

3CIM$C$9$16 ETRGFPAV

3BHG$A$88$95 QTNHDVKA

1S7Z$A$73$80 DTKDVIRI

2YA0$A$294$301 GTTHHMTK

3VC1$A$126$133 VTLSAAQA

1BF2$A$346$353 NTYNTVAQ

4DJA$A$345$352 KTHMNCMA

2WAN$A$593$600 ATEHPMAQ

3OHS$X$1158$1165 LTHVPRAV

4MZC$A$26$33 KTECPYCI

4HHR$A$513$520 LTEDEEAI

2C4W$A$51$58 QTNFEGEI

Additional file 4

N3Q – xxxxxxQx

1RIS$A$12$19 PNLDQSQL

3MBG$C$119$126 DLPTPEQQ

4HEC$B$63$70 LWRSRQQI

1YU0$A$8$15 RGGTTAQH

3ITF$B$49$56 ISLTEHQR

1SL9$A$54$61 LEATPEQT

3TG2$A$194$201 AVKSTQQA

1QMG$D$304$311 FATTLEQE

2IDL$B$74$81 KDLPSHQR

3VJZ$B$39$46 SCLSDDQH

2IP1$A$360$367 MTDTPKQI

1KGD$A$784$791 YFVSHDQM

3O1N$B$90$97 QALTTGQY

3MTW$A$363$370 YGATPLQA

2OLR$A$372$379 SRLTADQT

3CLM$A$67$74 QNLSPKQR

4EU9$B$442$449 RGLSPVQR

2WBM$A$86$93 IQLTAEQR

2W3P$B$218$225 EVVKPNQF

3RF0$B$88$95 PGFNQEQQ

N3E – xxxxxxEx

3TR9$A$207$214 YGKNVSEN

3DEO$A$226$233 RGLTALEL

2GZ4$D$177$184 IPLPSTEA

3LPZ$A$17$24 AEGQPEEQ

2A2C$A$275$282 LGISLEEM

1IZC$A$74$81 GMFNRLEL

3U9G$A$127$134 SGLNQEEL

1TXG$A$113$120 SVLTVPEA

3KB9$A$265$272 HSLTLEEA

2JIS$B$45$52 EWKEPEEL

4EQB$A$232$239 GVTFSGEA

3BVU$A$375$382 QFGTLQEY

4AMM$A$503$510 TALSRPEL

2QTZ$A$672$679 VGVEKLEA

3H74$A$136$143 ILPNTTEA

2PPX$A$40$47 LKLTQEEF

3OOX$B$293$300 ESITADEF

3M66$A$305$312 LGFKHNEI

2ELC$A$11$18 EVLEEEEA

3OF4$C$100$107 RQLTNEEA

N3H – xxxxxxHx

4HTY$A$88$95 KRFSKKHF

1PX4$D$462$469 SGHGANHD

3DNU$A$367$374 DKIYPRHF

3QTA$B$98$105 DGFTQLHE

1KQ3$A$246$253 GGLAAAHA

3GRH$A$23$30 QNFDAQHY

1UWC$B$241$248 QGVNDAHT

2WDC$A$425$432 QAITWDHL

3OC9$A$13$20 IPVTKEHY

1X6V$A$422$429 NPVHNGHA

3DO8$B$10$17 EPLHEGHK

3C8Z$A$49$56 DATHLGHA

3O0F$A$141$148 TTIGRPHI

3H05$B$12$19 NPPSLGHK

1NUU$B$16$23 NPITNMHL

3ELB$A$223$230 DLFHIGHV

4MAX$A$77$84 AGLTDVHF

2ZP1$A$39$46 GKIHLGHY

2G62$A$149$156 IDYGTGHE

3MLA$B$12$19 DPPHYGHL

N3T – xxxxxxTx

2RCC$A$239$246 ELNTEETK

4B3X$A$155$162 DGIMPQTE

1U7P$A$143$150 DGMSLQTL

3ZPY$B$356$363 KISGYVTE

3HFT$A$240$247 TSAGSRTF

2WM8$A$144$151 NGMNLQTL

2EK9$A$318$325 GKKNRVTD

3PC3$A$426$433 GVVGQETL

3MDU$A$74$81 PNDSFWTW

2F7B$A$199$206 QKPNFATF

4BOL$B$219$226 GELEKDTR

2G76$B$214$221 GLLNDNTF

2ESS$A$45$52 RGFGIATL

2DEB$B$503$510 RPASIFTK

3QIT$D$71$78 TSYSSLTF

2RB7$B$284$291 AADSPYTE

4IKV$A$252$259 GWFTVETF

2DYU$B$79$86 DVPGKETE

2JH3$D$72$79 LSEGYVTE

3DI4$B$148$155 TRCDPYTG

Additional file 5

N’’’P – Pxxxxxxx

2YPO$A$309$316 PNMTPELA

1B59$A$116$123 PSVPICDL

2FQX$A$300$307 PNLSSAVM

3UMO$A$142$149 PGVKLEKL

4G9Q$A$174$181 PDLAPRDR

2G3W$B$33$40 PSETDERL

2V2G$A$187$194 PGVSAEEA

3OP6$B$24$31 PAYTAQEI

4A0D$A$948$955 PDIKLYPF

2FYF$A$355$362 PAVEPDDV

1TBF$A$658$665 PGVSNQFL

2GGC$A$34$41 PGVSTGEL

4E1O$A$174$181 PDADESSL

1Y57$A$485$492 PECPESLH

4MO4$D$254$261 PALPAADI

3MOZ$A$272$279 PSVSLKDI

2VZC$B$342$349 PKPRPEDI

3UQ8$A$28$35 PSVPDSEY

3A5Y$D$9$16 PSASIPNL

4LHS$A$99$106 PNLTRVAQ

Additional file 6

NCapT-N3N – xxxTxxNx

3JZ0$A$138$145 NRLTEENA

4LRD$A$220$227 FGRTVMNE

3NDC$B$147$154 AGETLENF

3I8B$A$391$398 ANTTRENL

4AAN$A$167$174 DPVTFDNV

2IST$A$111$118 PDGTVLNA

3L6T$B$5$12 TTITRQNV

2ELC$A$168$175 GVRTVFNL

NCapT-N3R – xxxTxxRx

4FWW$A$529$536 HFLTCGRC

1BIF$A$130$137 TNTTRERR

3K13$C$487$494 QADTAARK

3D22$A$28$35 LITTKERW

1IOM$A$232$239 EIGTPERA

1PJ5$A$92$99 VATTETRL

3ZL8$A$173$180 FFGTRERI

Additional file 7

NCapD-N3T – xxxDxxTx

3LCC$A$183$190 YKVDVSTF

2PN2$A$98$105 QELDDKTQ

2VQX$A$304$311 QDADFATF

3UV2$A$2844$2851 EPMDLATM

3G1P$B$199$206 NHCDLNTV

1UH4$A$234$241 AFGDNSTL

3DA8$A$168$175 DGDDEETL

2C1L$A$139$146 LLLDNNTT

3LHO$A$135$142 WDVDKATY

1WPN$B$10$17 QNPDTDTI

3CC1$B$321$328 RDNDEWTL

1OFD$B$692$699 DRIDLPTA

4F3V$A$48$55 GDTDRVTL

4MDY$A$127$134 AGLDADTY

3ELF$A$64$71 GVKDMVTG

3V7N$A$401$408 TMIDTHTA

2E1V$B$171$178 CLGDASTR

1ZZE$A$276$283 GTFDWNTV

4N30$A$201$208 GMADETTL

4A4A$A$790$797 KDSDDWTK

NCapD-N3S – xxxDxxSx

1IOM$A$199$206 TETDLYSA

3NZE$B$303$310 LILDEASA

2DEJ$A$122$129 LKLDSLSK

1PJ5$A$475$482 PARDAWSG

1CY5$A$61$68 LKKDNDSY

2FSR$A$40$47 GPYDLPST

4AT0$A$337$344 LIIDEASY

2FW5$A$56$63 ASLDEASR

3NMW$B$553$560 WRADVNSK

2HLY$A$26$33 QNADPASA

3OWA$D$88$95 IGLDKVSS

2CH5$A$225$232 QQGDPLSR

3OG2$A$568$575 YMVDRNSA

2QSI$B$105$112 KIQDWSSY

2W5W$B$198$205 ALTDFSSI

3F81$B$143$150 QKMDVKSA

4HTL$A$205$212 DAHDAVSE

2Q7D$A$68$75 DQNDSQSL

1X91$A$58$65 GGVDPRSK

NCapH-N3H – xxxHxxHx

2CFU$A$168$175 SHAHADHF

3DHA$A$107$114 SHLHFDHA

1WRA$B$109$116 THTHSDHI

3M8T$A$100$107 THAHLDHT

3MD7$A$85$92 THPHADHI

1ZKP$A$58$65 SHYHHDHV

2P18$A$75$82 THKHWDHS

Additional file 8

N’’’R-N3E – RxxxxxEx

2D2E$A$111$118 REVGVAEF

4ASM$B$331$338 RSPNAAEM

2VQQ$A$60$67 RKATLEEL

1ZCJ$A$611$618 RTISKEEI

2YJG$B$109$116 RGTTLQEM

3R3R$A$151$158 RPLSDAER

2OUW$B$5$12 RLLDDAEI

4GD5$B$90$97 RDLKGEEK

3PES$B$61$68 RKLTEAER

3T94$F$169$176 RFSTRAES

3PT1$A$73$80 RPFTEEEI

3DB2$C$344$351 RPVEIAEI

3OL3$B$38$45 RSLTEDEV

1HDH$A$93$100 RVVALPEL

4F9D$B$215$222 RYETAAEY

1GVG$A$89$96 RSLLTMEA

4N13$A$64$71 RDLTKEEI

3KH1$B$36$43 RRENDAEH

1BAM$A$155$162 RVTNFEEL

3Q60$A$513$520 RRLLPLEA

N’’’D-N’R – DxRxxxxx

2PST$X$57$64 DMRPLSLR

3D1P$A$45$52 DVREPSEY

1GMX$A$25$32 DIRDPQSF

1P5D$X$356$363 DQRDSEHV

2WLR$A$24$31 DTRPSAFY

2EG3$B$136$143 DVRSPEEF

3G5J$B$19$26 DVRTEGEY

1YT8$A$287$294 DVRTPEEY

4GVF$B$182$189 DPRPETDI

2FSX$A$26$33 DVRCEAEW

3BV4$A$89$96 DGRPFPQV

1YWF$A$54$61 DLRSSREV

3I2V$A$21$28 DVRPQVEV

3E6Q$A$70$77 DGRDAATR

4F67$A$131$138 DTRNDYEY

1QB0$A$425$432 DCRYPYEY

2HHG$A$43$50 DIRDPREI

1MIX$A$205$212 DSRDPVQL

N’’R-NCapE – xRxExxxx

3SZ7$A$126$133 ARKEYSKA

2CFU$A$461$468 ARGEYRWV

1TKE$A$150$157 NRGESYKV

3JZ0$A$164$171 KRGEYARS

2NW8$B$77$84 QRDEVWQC

2I0O$A$83$90 GRKEFEKA

2QEU$A$77$84 IRDEPIGI

N’D- N1S – xxDxSxxx

4N21$A$327$334 IDDFSKNV

3TIA$A$101$108 SKDNSIRL

1G87$B$498$505 FMDLSEIV

1YON$A$146$153 DGDYSYLA

2QZU$A$381$388 TFDLSNEV

1N7H$B$322$329 QGDASKAK

3UJC$A$203$210 SKDLSDYW

4KG7$A$211$218 TIDQSVLG

N’’E-NCapR-N3D – xExRxxDx

3H9C$A$466$473 QEGRDADL

3R0V$A$140$147 AEGRRGDA

3EE4$A$84$91 AEGRLADE

3FKR$A$244$251 REGRHDDA

4N7Q$A$534$541 FEFRWMDQ

N’’’E-N’K-N3E – ExKxxxEx

2JIS$B$45$52 EWKEPEEL

2I71$B$322$329 ERKLLGEY

3PNX$F$56$63 EDKSLYEQ

3FDJ$A$132$139 EGKKFEEI

2NWH$A$265$272 EGKTIREA

Additional file 9

N’’T-N4T – xTxxxxxT

1U69$D$81$88 ATDDQAET

3KSM$B$230$237 FTPNESTT

3MD9$A$30$37 VTIGGDVT

3QSL$B$212$219 DTRTLKDT

4G3O$A$482$489 LTRSVEIT

4MLZ$A$81$88 VTLKSSTT

4ES1$A$10$17 QTSSMGGT

1N2Z$B$27$34 ITLSPANT

N’’T-N4N – xTxxxxxN

3W5N$A$603$610 ETNVPMLN

2G1U$A$79$86 FTNDDSTN

2X32$B$51$58 NTGIDVRN

2XRY$A$354$361 KTHDPLWN

3TD3$A$272$279 NTGPRKLN

Additional file 10

N’M-N4F – xxMxxxxF

2Y7P$A$184$191 SPMSLKQF

2XRH$A$78$85 DKMTVADF

4GMU$A$115$122 RWMSEEDF

1Y57$A$300$307 GTMSPEAF

4IHM$A$223$230 SDMTQDDF

1XKG$A$61$68 FLMSAEAF

3ONH$A$454$461 NKMKLSDF

2O6X$A$64$71 TDMTFEEF

3M8J$B$68$75 YQMNNGYF

3TG7$A$758$765 CNMTKDWF

3L9U$A$131$138 AGMSQADF

4AY7$B$211$218 DLMSPDSF

2RK5$A$16$23 GTMTLNDF

3ZHO$B$40$47 ETMPPQLF

2R9F$A$334$341 FWMSFRDF

2QF7$A$838$845 HEMPGGQF

3PUA$A$170$177 CKMKLKEF

3UQ8$A$175$182 VFMPHAGF

N’V-N4L – xxVxxxxL

1VPK$A$3$10 VTVTTLEL

3QBM$A$149$156 PEVDADRL

2QHF$A$92$99 DPVDPAEL

3NUF$A$79$86 AEVSQKSL

1YOC$B$57$64 GTVHAIAL

2XCJ$B$27$34 TGVPYGTL

2GLZ$B$96$103 FTVSPAVL

3EYT$A$133$140 GDVSELLL

3E78$A$48$55 FGVSEAWL

3HRR$B$1623$1630 RSVPRGAL

3G98$B$773$780 EEVEPEEL

3K3T$A$171$178 LGVPAHFL

2GKG$A$105$112 KPVDADQL

2BVF$B$131$138 PKVGFCGL

3VKW$A$1099$1106 ERVSSYLL

3FD5$A$31$38 CKVPQDVL

2C29$F$227$234 QFVHLDDL

3FL2$A$741$748 HNVCKDCL

1DMS$A$58$65 PMVRREFL

2XOD$A$46$53 GNVPERVL

N’I-N4L – xxIxxxxL

1ODM$A$47$54 HGINVQRL

1GTE$D$148$155 GSINIGGL

1TJV$D$211$218 LDIDREML

1KJQ$B$84$91 EAIATDML

1OFD$B$48$55 TAIPRELL

3QXH$A$92$99 APIDTDNL

8ACN$A$14$21 EYIRYDLL

2QJZ$B$89$96 KIIPVDKL

3C1D$B$113$120 ADIDWAAL

1GVF$A$187$194 PKIDFQRL

3OND$A$353$360 NEIDMLGL

3HHI$A$144$151 VHISAGDL

3B1B$A$238$245 TTIKLGEL

N’’A-N4A – xAxxxxxA

3FSY$E$78$85 VAADPYDA

3W37$A$765$772 EAMTTQAA

1B93$A$116$123 VATNVATA

1VQ3$D$50$57 EAENKEKA

4EAD$A$289$296 LAKDDAEA

4MYL$A$82$89 TASTLEEA

1RA0$A$380$387 PAENGFDA

4O0K$A$79$86 GAVNTASA

2QF7$A$408$415 WAPNPLEA

1M22$B$264$271 MARSVADA

2I7G$B$239$246 IADTTDKA

2WW5$A$331$338 YAENETDA

3IRS$C$98$105 EAATRKEA

3DME$B$93$100 VATSDAEA

4KJM$B$109$116 GAQRVSNA

3LUF$B$151$158 EASHAREA

1UEK$A$240$247 LAEGPDHA

1A3A$D$17$24 KAATKEEA

2W3W$A$82$89 VAGSLEAA

3TTC$A$330$337 PAISNEQA

N’’V-N4L – xVxxxxxL

1ZSW$A$84$91 LVPSEDSL

4HWG$A$7$14 IVGTRPEL

1T5O$D$43$50 AVRGAPAL

4HWV$B$60$67 TVANRNEL

2NXW$A$492$499 RVRTRAEL

2YXX$A$65$72 DVVTKGEL

1C3J$A$299$306 YVNNRAEL

2IN3$A$126$133 DVAQLAIL

3FLA$B$65$72 PVDSIGGL

1OJ7$A$91$98 AVGGGSVL

3ZSC$A$31$38 FVRTAEEL

2Y88$A$202$209 GVSSLDDL

1K30$A$10$17 DVRSEEEL

1HM9$A$225$232 GVNDRVAL

4KOA$A$51$58 AVTSVDDL

4MZC$A$84$91 VVGGCDDL

3KEV$A$73$80 QVDSLEKL

2ABW$B$34$41 QVRNVHDL

2C0A$C$73$80 TVLNPQQL

3RGA$A$44$51 PVVGRAAL

N’’F-N4L – xFxxxxxL

2QIB$B$53$60 YFPGKLSL

3KSX$A$64$71 EFPAGPQL

1VL2$D$151$158 KFKGRTDL

2OB3$B$72$79 FFGSRKAL

3BKB$A$519$526 GFPSIPLL

1MUN$A$58$65 RFPTVTDL

4LHD$B$53$60 GFDTLGDL

3UEK$A$542$549 KFTRPQNL

3G7R$B$54$61 HFSGKDDL

2Q24$B$54$61 NFPTREAL

2W91$A$180$187 KFNSWEEL

2VUW$A$783$790 NFSSATDL

2HXI$B$47$54 HFRNKTEL

2SHP$B$186$193 RFDSLTDL

2G3B$B$43$50 HFKDRIGL

3BKX$B$133$140 YFASANAL

4FL3$A$233$240 KFDTLWQL

3SUV$A$444$451 TFVTQDHL

1NB9$A$115$122 NFDSLESL

4AQN$B$149$156 NFDNISQL

N’’L-N4I – xLxxxxxI

3HFW$A$26$33 FLQDGEKI

2IJ2$B$51$58 YLSSQRLI

3EIN$A$62$69 ALWESRAI

4LIM$A$248$255 ALYDKQLI

2HNL$B$84$91 VLGESHAI

3HN7$A$458$465 VLSSVDDI

2YCD$A$82$89 ILFESGAI

4JBB$A$68$75 ALSESSAI

1A3A$D$121$128 ALDDESVI

3E10$A$46$53 VLEDRENI

2V6K$B$61$68 VLIQSPAI

1XSV$B$92$99 HLSNPEQI

4MPG$B$63$70 ILTESSAI

3I7M$A$20$27 YLSNPKTI

2V0C$A$458$465 DLKDVEDI

1ZR3$D$349$356 VLFDSESI

2JII$B$393$400 CLPNTEDI

2QPX$A$199$206 RLTSKPLI

3BHD$A$81$88 ELTAEPTI

4GMQ$A$412$419 GLTVADEI

1ZQ9$B$38$45 ILKNPLII

4MG4$H$191$198 GLRSPALI

1M22$B$416$423 GLADPAYI

4NYH$C$189$196 CLERQNYI

Additional file 11

N’I-NCapD-N4V – xxIDxxxV

3HGU$B$164$171 IDIDPRWV

4EDP$A$325$332 KVIDFKFV

4F9D$B$317$324 MHIDLDYV

3ESS$A$607$614 LAIDEEAV

1O54$A$39$46 GIIDLNEV

2W86$A$105$112 EDIDECEV

2GAI$B$127$134 REIDMKKV

4BVQ$B$264$271 DAIDAASV

N’V-NCapT-N4I – xxVTxxxI

2GUF$A$27$34 TVVTRQDI

3U1D$A$92$99 YAVTGEGI

3OI8$B$167$174 GLVTFEDI

3JTM$A$101$108 AYVTAERI

1O4Y$A$225$232 RTVTRAEI

2QHF$A$26$33 VHVTSADI

3E7H$B$40$47 RRVTARHI

2YMM$D$83$90 AKVTPDDI

3OL3$B$60$67 SPVTDDDI

3GHD$B$49$56 GVVTERDI

4LVF$A$73$80 KVVTKEKI

2G76$B$56$63 TKVTADVI

3H63$C$359$366 DGVTLDDI

N’V-NCapS-N4I – xxVSxxxI

3GBY$B$110$117 GVVSRKRI

2G50$H$218$225 PAVSEKDI

3CH0$A$221$228 TLVSKKDI

3AHC$A$15$22 RPVSEEAI

2QIB$B$32$39 DEVSIDEI

1LW4$A$321$328 KDVSRNDI

4FB7$A$23$30 ASVSLSEI

3IM1$A$1856$1863 YGVSFFTI

N’h-NCapS-N3E-N4h – xxhSxxEh

2A2C$A$275$282 LGISLEEM

4AMM$A$503$510 TALSRPEL

4GVF$B$307$314 GSFSRREL

1VKK$A$50$57 QNISPEEL

2A2C$A$304$311 LGISLEEL

1EX7$A$51$58 NFVSVDEF

3FMU$A$155$162 AGFSPVEV

1YQT$A$452$459 NELSGGEL

4G1I$B$265$272 NTMSKKEL

3N3R$B$624$631 LTLSAPEM

1I24$A$296$303 EQFSVNEL

1YT3$A$261$268 LGLSGSEI

4L2H$A$119$126 AAFSREEI

3IUP$A$343$350 KEISLAEV

4KWD$D$188$195 LKLSPSEL

2C5A$B$273$280 EMVSMNEM

1YQT$A$209$216 QHLSGGEL

2HBW$A$86$93 KSFSESEI

3NTX$B$315$322 QSLSPNEI

4DQ6$B$332$339 LGLSDEEL

N’h-NCapT-N3D-N4h – xxhTxxDh

1CEO$A$27$34 TFITEKDI

4F03$A$200$207 GGITYSDI

4JED$A$155$162 DRFTLGDI

4C45$A$84$91 GELTKKDV

1PBJ$A$45$52 GIVTTWDV

4F0C$A$164$171 DKLTIADI

2WOL$A$113$120 TPITSDDV

2O7I$A$81$88 VPITADDF

3N9K$A$73$80 TWITEQDF

4FFU$L$22$29 RTITETDF

4E3E$B$27$34 RTITEGDV

3PJ0$A$123$130 QLLTIDDI

2X8H$A$272$279 YGITSDDL

4MPG$B$160$167 QQVTLADL

3EIN$A$151$158 DSLTVADI

Additional file 12

N’F-N4Y – xxFxxxxY

3RF0$B$118$125 NLFKKKYY

1OZ2$A$420$427 DNFCWEKY

2A14$A$13$20 KHFLPRDY

3ETN$A$175$182 TEFTIEEY

1KWF$A$214$221 SYFAPAWY

1Q0R$A$191$198 VPFDDAEY

1BIF$A$70$77 REFNVGQY

3FED$A$583$590 IPFNIQDY

3H8U$B$12$19 RIFSVDEY

3ZUZ$A$214$221 LKFDPEYY

3VQT$D$237$244 TPFDEERY

3OLQ$A$211$218 PDFDPNLY

N’F-N4F – xxFxxxxF

4DMV$A$163$170 PQFDNMKF

3O0Y$A$576$583 DKFDLAEF

1KT6$A$13$20 ENFDKARF

3ISQ$A$357$364 QGFGAGNF

4MZ7$A$327$334 NNFDYKRF

3RRC$A$483$490 DEFNEVAF

4HFQ$B$129$136 AVFDTNRF

3O22$A$32$39 PNFQQDKF

3PVJ$D$155$162 KSFPLERF

1WKO$B$144$151 DHFNTRKF

2HY7$A$231$238 MLFDPEFF

3AMN$B$194$201 VKFDVRHF

3SIB$A$201$208 KPFNPMEF

3G02$B$70$77 SEFDWRPF

2C42$A$306$313 RPFVSEAF

2NLR$A$172$179 WSFDVMDF

2JG0$A$86$93 SGFDLRHF

2IQY$A$147$154 GKFKVESF

N’F-N4W – xxFxxxxW

3KB9$A$196$203 LTFAHWIW

4LVF$A$94$101 DVFNEKGW

4E1O$A$299$306 FTFNPSKW

3LB2$B$113$120 QSFDSQSW

4HTL$A$82$89 DNFNLKEW

3JZ0$A$53$60 SNFDSSNW

Additional file 13

N’’P-N3F – xPxxxxFx

2O8N$A$115$122 RPNKPLFT

1R5L$A$45$52 LPLTDSFL

4MZ7$A$211$218 GPFSHMFD

1GSA$A$89$96 PPFDTEFI

4BT7$A$230$237 LPHTDDFA

4DMG$B$71$78 GPLNRAFF

N’’P-N4F – xPxxxxxF

4GVE$A$413$420 KPHDEKGF

3IM3$A$40$47 RPERPMAF

1X38$A$317$324 VPNKYQQF

3G36$D$76$83 RPPNPIEF

1VEM$A$233$240 PPSDGEQF

3MWP$C$416$423 EPTDLKQF

2FTR$B$16$23 QPEDKQAF

Additional file 14

N’’D-N’G-NCapT – xDGTxxxx

3KB9$A$302$309 ADGTVRGK

1WCX$A$116$123 GDGTSKSL

1WD3$A$426$433 NDGTKQFH

3KMV$A$82$89 NDGTSLFA

2X1D$A$279$286 FDGTKQAF

1UWK$B$508$515 CDGTDEAA

1R7A$B$15$22 GDGTIKSM

N’’T-N’G-N4D – xTGxxxxD

1OWL$A$328$335 QTGYPIVD

2PEB$A$86$93 ETGDAVSD

2J4D$B$370$377 KTGYPLID

2P8I$A$89$96 NTGDALRD

4MLP$D$352$359 KTGFPWID

2Y6X$A$29$36 LTGNFRED

4IR8$B$238$245 YTGGLVPD

NCapH-N2G-N3H – xxxHxGHx

1X6V$A$422$429 NPVHNGHA

3DO8$B$10$17 EPLHEGHK

3C8Z$A$49$56 DATHLGHA

3ELB$A$223$230 DLFHIGHV

2ZP1$A$39$46 GKIHLGHY

3MLA$B$12$19 DPPHYGHL

2V0C$A$46$53 GDLHMGHL

2YXN$A$45$52 DSLHLGHL

3GLV$B$11$18 DILHLGHI

3H9C$A$18$25 GSIHLGHM

Additional file 15

N’’’D-N’S – DxSxxxxx

3AJR$B$47$54 DVSNRDEI

4KWD$D$113$120 DRSIPVEY

1ZXT$D$54$61 DPSKNWVR

2QQ5$A$64$71 DSSQESEV

3O0F$A$92$99 DPSNEHIS

4GCO$A$241$248 DPSNEEAR

2DB7$B$37$44 DASDPLRV

3TN2$A$53$60 DPSESWVQ

3KLK$A$987$994 DNSNPVVQ

2YFO$A$449$456 DFSRKEVR

2IF6$B$154$161 DLSNPLVQ

2XSA$A$343$350 DPSDPAWR

3IPC$A$51$58 DVSDPKQG

4H04$A$289$296 DISNPEAV

2YA0$A$455$462 DPSKAENY

4EVQ$A$80$87 DESAPPKA

4LTN$A$54$61 DLSNAKLK

3SG0$A$67$74 DESDPTKA

2AHF$B$297$304 DESDPERQ

3DSK$B$158$165 DPSEPKHE

N’’’D-N’T – DxTxxxxx

3MPR$D$182$189 DQTHQSYD

1W9I$A$6$13 DRTSDYHK

3VX0$A$177$184 DTTKDVVK

3HR6$A$98$105 DLTTQAGW

2OC5$A$24$31 DFTSDRYK

3HL1$B$37$44 DPTTVPYR

3BJN$A$167$174 DPTLDKWQ

4ILY$B$207$214 DSTSFGSI

3UXF$A$90$97 DLTTYDGW

3H7C$X$244$251 DETDPQYE

N’’’D-N’R – DxRxxxxx

3FXG$A$104$111 DPRNTNLL

4HCH$A$74$81 DHRDRERL

3PZJ$B$158$165 DSRNAASA

2QE6$A$135$142 DVRDPEYI

3IEI$H$171$178 DLRDLSEL

N’’’D-N’’P – DPxxxxxx

1ZI8$A$74$81 DPQDERQR

3SZ7$A$178$185 DPKYSKAW

1H41$B$258$265 DPLDPRVQ

2RA4$B$53$60 DPKEKWVQ

1ZXT$D$54$61 DPSKNWVR

3MBC$B$485$492 DPERAHDR

3O0F$A$92$99 DPSNEHIS

2YB1$A$82$89 DPAEPALA

4GCO$A$241$248 DPSNEEAR

4HVT$A$613$620 DPEIPNDL

2C29$F$38$45 DPTNVKKV

3FP3$A$560$567 DPRSEQAK

4MPC$A$102$109 DPEDHRTL

3TN2$A$53$60 DPSESWVQ

4ML1$D$100$107 DPRCAVCH

4FWW$A$98$105 DPGCQTCA

3GWZ$A$102$109 DPASPVAT

2XSA$A$343$350 DPSDPAWR

3FP3$A$157$164 DPNEPVFY

4GCO$A$173$180 DPENAILY

NCapD-N1P – xxxDPxxxx

2UVJ$A$362$369 KDEDPSVA

3CI3$A$22$29 YKNDPRVA

1M22$B$416$423 GLADPAYI

2XRY$A$409$416 DGRDPNGY

4MQL$A$180$187 PSSDPAWK

3OZP$A$450$457 TGVDPQIK

4F2L$A$315$322 NHQDPLAV

4EX6$A$66$73 PVEDPRVA

3BL9$B$322$329 RADDPLLK

4J7D$A$73$80 CSNDPLGE

3NYH$A$145$152 PKNDPKLK

3DMG$A$29$36 GYRDPVHD

2HBG$A$49$56 GASDPGVA

4DTH$A$253$260 GLTDPKVK

2VWG$A$209$216 DRPDPTID

1XDN$A$291$298 RRGDPAVE

2HEU$C$208$215 KLSDPIMK

3VAY$A$154$161 GKPDPAPF

2B3F$A$195$202 KFTDPKAV

2NO4$A$151$158 YKPDPRIY

N’’P-N’D – xPDxxxxx

3LHQ$B$81$88 FPDDPLSV

3EKG$B$246$253 PPDDYWGY

2YG5$A$62$69 SPDQTALI

1UWK$B$384$391 IPDDAHLH

2QSA$A$107$114 HPDQRFYN

4G32$A$475$482 LPDYPYRD

2NRK$A$142$149 HPDSIDKY

4J0U$A$211$218 NPDNSYIK

2PZM$B$81$88 DPDDWAED

2XAU$A$574$581 HPDGDHIT

4KNU$F$167$174 NPDDKTAM

4A35$A$284$291 SPDDILGH

Additional file 16

CCapD-C’P – xxxxDPxxx

2B7U$A$211$219 KSNADPCYK

2BVF$B$439$447 KREYDPENR

1N8V$B$85$93 TAKYDPTGN

2J4D$B$454$462 AQNYDPEGE

3ZVL$A$463$471 REMTDPSHA

3QXH$A$85$93 QQEEDPNAP

3FWA$A$501$509 KTLIDPNNV

3W8Z$B$444$452 KAKYDPTNL

2Y08$A$480$488 KARWDPRNV

CCapD-C’P-C’’T – xxxxDPTxx

3CMG$A$448$456 AKQEDPTRP

4J7A$D$264$272 VKPYDPTGE

4BBY$A$643$651 KDYVDPTNI

2GQW$A$303$311 RHLVDPTAP

1XNF$B$122$130 VLELDPTYN

1QW9$B$201$209 MKWVDPTIE

CCapD-C’P-C’’S – xxxxDPSxx

1QZR$B$74$82 NKVRDPSMK

2DY1$A$405$413 LLEEDPSLK

4GCO$A$237$245 ALQVDPSNE

4MLP$D$437$445 GRRTDPSGD

1PX4$D$475$483 IKSVDPSRP

1V0L$A$158$166 ARAADPSAK

Additional file 17

C3R-C’’E – xRxxxxExx

3BFM$A$171$179 NRWDEGELE

4BGB$A$335$343 LRYLEGEPV

2YFD$A$60$68 ARQAAGEAL

3M8U$A$415$423 KRTKAGELT

4EQL$B$73$81 DRVVNGESS

2VW8$A$250$258 WRQSMGESL

1ES9$A$82$90 WRLENGELE

1MO9$B$372$380 ARNVMGEKI

2IWK$A$368$376 IRAYAGEKI

1Z05$A$278$286 ARIQAGEPS

1J09$A$136$144 ERARRGEPH

1E58$A$170$178 PRMKSGERV

2R78$A$76$84 ARIVGGEPL

C3E-C’’R – xExxxxRxx

3ZBO$B$219$227 KELDRGRLG

2AQ5$A$377$385 EEWLGGRDA

3U42$A$154$162 REIKAGRIE

1YKI$A$101$109 QEDADGRFA

3LOG$A$196$204 DEIAAGRYH

3ARL$A$141$149 YECLDGRCS

3C8M$A$181$189 RELANKREF

3G91$A$138$146 RERDSGRNV

C3D-C’’R – xDxxxxRxx

1TV4$A$355$363 TDTASGREI

3GVO$A$712$720 EDFRNNRFP

2DKV$A$268$276 ADAAAGRAP

2WNW$B$107$115 ADLQQGRLS

3KBR$A$90$98 RDFADDRFD

Additional file 18

C4E-CCapR-C’’E – ExxxRxExx

4ERC$B$83$91 EANARGEAV

3Q62$B$14$22 EASGRGELF

3ZXY$A$234$242 EQVRRGETP

2V03$A$51$59 EAEKRGEIK

2IST$A$160$168 ESLQRREIT

2I0O$A$80$88 EAYGRKEFE

1VK1$A$210$218 ELVKRGEVF

1P5D$X$42$50 ESLARGEPC

2IEA$B$855$863 ELAKRGEID

2V03$A$128$136 EMANRGEGK

Additional file 19

CCapD – xxxxDxxxx

3SIB$A$57$65 FMGVDRDRS

4IHQ$C$132$140 PLIRDPYIE

3RPW$A$157$165 ADFWDVKKF

1FDS$A$265$273 MRLDDPSGS

3K21$A$128$136 FRVFDVDND

3CBW$B$152$160 KKILDSSTV

2GQW$A$390$398 AALADPATD

2H5Y$A$173$181 DARADAKVR

2XF3$B$343$351 DSGRDTSGT

3AKA$A$108$116 LGVADTDGD

4MLW$A$106$114 FSLYDVDGN

1EXR$A$89$97 FKVFDRDGN

2EGD$B$60$68 MKSLDVNQD

3VIS$B$266$274 KRFVDEDTR

3TTC$A$164$172 KEYRDPLDR

1SG4$C$42$50 KLENDKSFR

3QXZ$C$41$49 RLDDDPAVR

3MSU$A$400$408 EMVNDPAQK

3B8B$A$16$24 SIYEDPKSD

2ZWU$A$73$81 EAYEDYRHF

CCapS – xxxxSxxxx

1JIG$D$76$84 YLATSSVNE

3TCV$B$70$78 KASKSDDPL

2WNW$B$73$81 SLYFSAQEH

2RBD$B$120$128 IIGQSIRED

4MZ7$A$429$437 EILYSTDPK

3EDY$A$429$437 FLSSSPHLP

2NT0$D$106$114 KSYFSEEGI

4E6U$A$252$260 SLEQSERGI

2QA1$A$338$346 LLFLSGPEV

3CT1$A$117$125 EYIKSTKTP

3P0B$A$271$279 LQVWSADYG

1XHN$D$26$34 VTHVSDWGA

3TC5$A$107$115 ASQFSDCSS

3AB8$B$136$144 VLRASPVPV

4B6G$B$59$67 FITKSGFQR

4LHD$B$180$188 SYGVSKSKA

3HXW$A$112$120 LLLTSEKWF

1O9I$F$45$53 QGWASTGAE

3FED$A$576$584 ELVDSKIIP

2CCM$B$171$179 EYFVSNDRG

CCapT – xxxxTxxxx

3MBC$B$351$359 AMIRTSGHM

4GIM$C$277$285 VAELTGGDS

3CKM$A$503$511 LAKSTGGEY

4N4U$B$50$58 IEKNTGGEV

1DCS$A$230$238 ATLVTGGQV

4KXV$A$548$556 SARATKGRI

1Y57$A$453$461 TELTTKGRV

1L3P$A$192$200 IKETTGGAY

3E2D$A$170$178 LEKLTQGDV

3CLM$A$93$101 EHESTGGKT

2V9L$A$128$136 RIKATNGKD

3B8L$F$20$28 HAVDTVSDI

4I93$B$49$57 IRNATSGFA

1R7A$B$322$330 IHANTHGES

1U14$A$140$148 IGVFTAGKL

2BRY$B$338$346 ADFATHGKL

4J0U$A$300$308 IKKATHNRP

3PZF$A$167$175 VSEHTNGRL

4JA8$A$88$96 NRDQTDDQV

3I4Z$A$273$281 EDLWTLGGR

CCapN – xxxxNxxxx

2HQ7$B$60$68 RLKKNNKIC

3SMJ$B$1586$1594 MDAKNGQTM

3QGU$A$62$70 HQEKNPDAK

2OB3$B$317$325 MDRVNPDGM

2C31$B$387$395 FMLANPDIS

3BVU$A$117$125 HLHDNPEMK

3PC6$B$595$603 ALMENPSLA

1OZH$D$432$440 AWLVNPERK

3F7C$A$105$113 LNRLNPNKH

3E78$A$133$141 FLKNNPGII

4I62$A$204$212 FVENNPDLA

3TM8$A$102$110 EVFENPENV

3PAJ$B$201$209 AKQLNPGKP

4GWG$A$378$386 AFDRNPELQ

4A4A$A$418$426 FKEKNQEAQ

4JXH$A$171$179 YCLCNPGVF

2OCZ$A$164$172 FKTLNPEQE

2YIC$A$962$970 YSVGNPDAM

2YLN$A$209$217 YLKKNPNAG

1B5E$B$115$123 ELGQNPDSR

Additional file 20

C1E – xxxExxxxx

4JWO$A$269$277 ATIESGEYT

3LRT$B$16$24 IAEELKTEP

3H4T$A$42$50 RCAEVGVPM

3IM1$A$2007$2015 KCKEINVET

1Q8I$A$114$122 YLMERFITS

3NEH$B$214$222 AMIEHDAMI

2P0K$A$33$41 YLKETCSVP

1RA0$A$300$308 EMLESGINV

4H7W$A$192$200 VMEEFNLTT

3LED$B$279$287 HAREIGIDP

1KQ3$A$217$225 RSVEEKSVT

1KLX$A$20$28 KACELNEMF

3E58$B$119$127 ALEENRLQG

3HQ1$B$127$135 EIIEQGAIP

2XAU$A$160$168 VAEEMDVKL

2WUQ$B$14$22 ALHEGGLTG

2VCH$A$126$134 VAVEFHVPP

2BIB$A$135$143 TATETGVSV

3OMT$B$13$21 VLAEKGKTN

2Y71$A$39$47 EAAELGLKA

C1N – xxxNxxxxx

1IXH$A$308$316 WKTNIKDSS

1K30$A$159$167 IAENTIFVA

2P8I$A$75$83 LTLNHGALD

2WBF$X$623$631 YVANCYKGE

1G87$B$416$424 YVNNEIACD

3PMM$A$34$42 NLVNIKDES

3IJW$B$93$101 IRDNVPAFE

4LIX$A$142$150 IAENQLSDG

4AIO$A$704$712 DWFNKLDFT

3T7H$B$92$100 EFKNLDKQL

2X5F$B$26$34 LGQNMFYPK

1JTA$A$352$360 ITDNAGSGK

3S5M$A$626$634 KYKNAEESP

4NBI$B$58$66 KCLNLRLWN

2CAY$B$62$70 PTQNSLGLE

3GMF$A$137$145 PLANSTEAQ

1OFD$B$121$129 AKNNQPHIE

1QCX$A$351$359 TMKNAGQGK

3TRQ$A$138$146 AFENIEDEI

2REG$B$253$261 LLQNLSFSL

C’R – xxxxxRxxx

4EBJ$A$108$116 RRRLDRPYK

2J91$A$189$197 VRDDLRFRG

3Q9O$A$7$15 IFDECRSPC

4EO3$B$147$155 HIEWRRARR

2YCD$A$132$140 VWLFERNEP

1QJ4$A$169$177 AKMLTRKGS

2C2U$A$82$90 YHWDIRGRF

1QW9$B$379$387 ASVYGRGVA

3AML$A$449$457 QTLTNRRYT

4LY1$C$80$88 FLRSIRPDN

3CZP$A$198$206 LRRTSRDYA

2VQR$A$258$266 YVDSIRRGS

4HVK$A$131$139 IDQKLRDDT

3H8U$B$16$24 VDEYVRPSN

4KRG$A$189$197 LLRAIRYRD

4NBI$B$58$66 KCLNLRLWN

1XGS$B$118$126 AISVARAGV

1RP0$B$225$233 IVRLTREVV

2WW8$A$370$378 LTQQARQNS

3F7C$A$166$174 ALYYYRTPK

C’S – xxxxxSxxx

2QE9$B$89$97 VLQTHSDQI

3IG3$A$1674$1682 FETVFSTAH

3O2T$A$190$198 LIVTLSPRM

3MWP$C$308$316 YKICLSGDG

1TU9$A$27$35 HFLASSPQI

2C42$A$895$903 AKALESDAS

4ATG$A$176$184 LSMIKSDMK

4B4U$A$109$117 CFDAISLAK

4EV1$A$148$156 DLLRQSGQQ

1QZR$B$148$156 LCNIFSTEF

2A9S$B$91$99 GALYRSRAN

3B6E$A$296$304 VAARASPEP

4AIO$A$848$856 PVQVNSSDT

2O7I$A$473$481 LDKAVSTLD

1HN0$A$703$711 LKDLDSPKP

2C4W$A$63$71 QESVGSEYE

1M0W$A$318$326 TQLSGSKKI

4G26$A$103$111 KLDMCSKKG

1NAR$A$112$120 IIQKYSDDS

4FZL$A$51$59 GDLYCSPNC

Additional file 21

CCapF – xxxxFxxxx

3PFE$A$420$428 LGEQFPKAQ

1JJF$A$204$212 NERLFPDGG

4ADN$A$103$111 LQKVFKKVK

3PPM$A$377$385 TGGLFSDGG

3E78$A$266$274 LKNHFQNKF

3IG3$A$1305$1313 VRVLFPGIE

2OHH$A$95$103 LHRRFPEAP

3PF6$A$28$36 ARLLFPGGT

3CGG$A$83$91 AKQDFPEAR

3B0P$B$191$199 LKGDFPQLT

3D3Y$A$156$164 QSVYFNQSE

1LCF$A$149$157 VARFFSASC

3OA5$B$241$249 FFQRFPMFS

3FRQ$A$177$185 LCLMFPEHD

3I3F$C$105$113 YREVFCVPA

1RJD$A$115$123 LLQMFPHLA

2DE3$B$199$207 GADLFPDVA

4B5O$A$7$15 VDALFPERI

2QIK$A$72$80 LDQFFQGYH

4MYL$A$124$132 MLRAFPGDV

CCapY – xxxxYxxxx

3KKZ$B$216$224 FLTKYAGNK

3PL8$A$226$234 LTEEYKGQR

2RBC$A$208$216 LHARYPQTF

1B59$A$120$128 ICDLYPNGV

1Q6O$B$50$58 LKALYPHKI

1O0W$B$69$77 LYKKYPEAE

4K7B$A$46$54 NSGAYPTNK

1G66$A$29$37 VLSAYPGST

3D22$A$71$79 LSENYPSLM

3QHB$B$78$86 LTKLYSELE

3B7C$A$54$62 YKKNYPDKE

3PF9$A$115$123 LAGLYPDLI

3H9M$A$73$81 IKKGYTNEW

2HSJ$A$115$123 VARDYPLTE

3HQ1$B$202$210 QAAKYPGTQ

4F03$A$95$103 LDETYPDTP

3JS6$A$308$316 LSHYYSDVF

4EBJ$A$12$20 LREEYADAV

2WB0$X$376$384 MQALYPNAQ

4G1I$B$236$244 LDTYYPQLA
