# Supplementary figures and images for: Mapping side chain interactions at protein helix termini
Source: BMC Bioinformatics. 2015 Jul 25;16:231. doi: 10.1186/s12859-015-0671-4 (PMC4515027; doi:10.1186/s12859-015-0671-4)

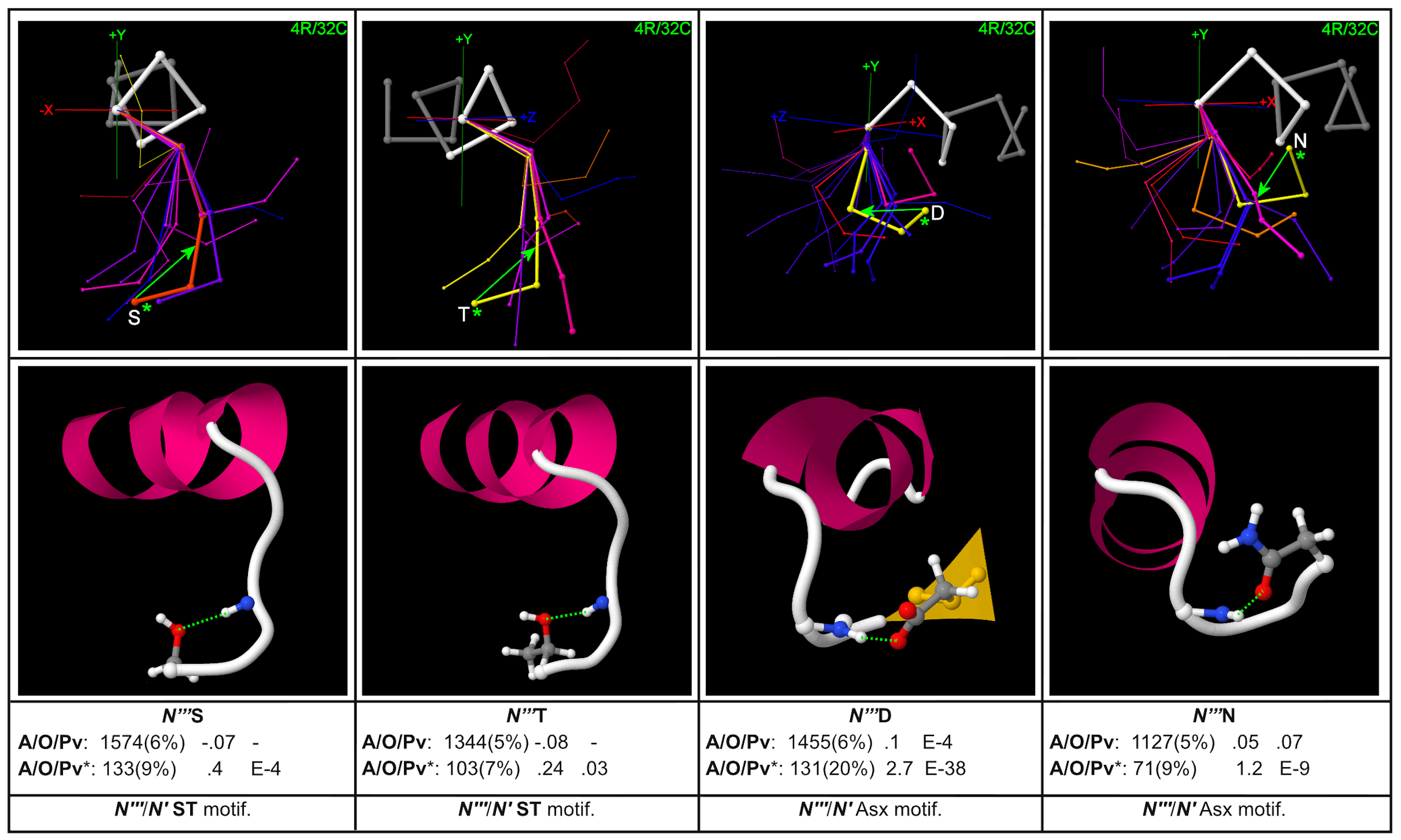

Supplement: Additional file 2: — Asx/ST motifs at N”’ . Maps for Asx and ST motifs that occur at N”’, with example structures and global and peak-cluster (*) motif data (Abundance/Overrepresentation/Pvalue). Exemplar width is proportional to motif abundance in the corresponding cluster/geometry, while exemplar colour is proportional to overrepresentation. Asx and ST turns each show characteristic geometries at N”’, as they do at NCap. [file 12859_2015_671_MOESM2_ESM.tif]

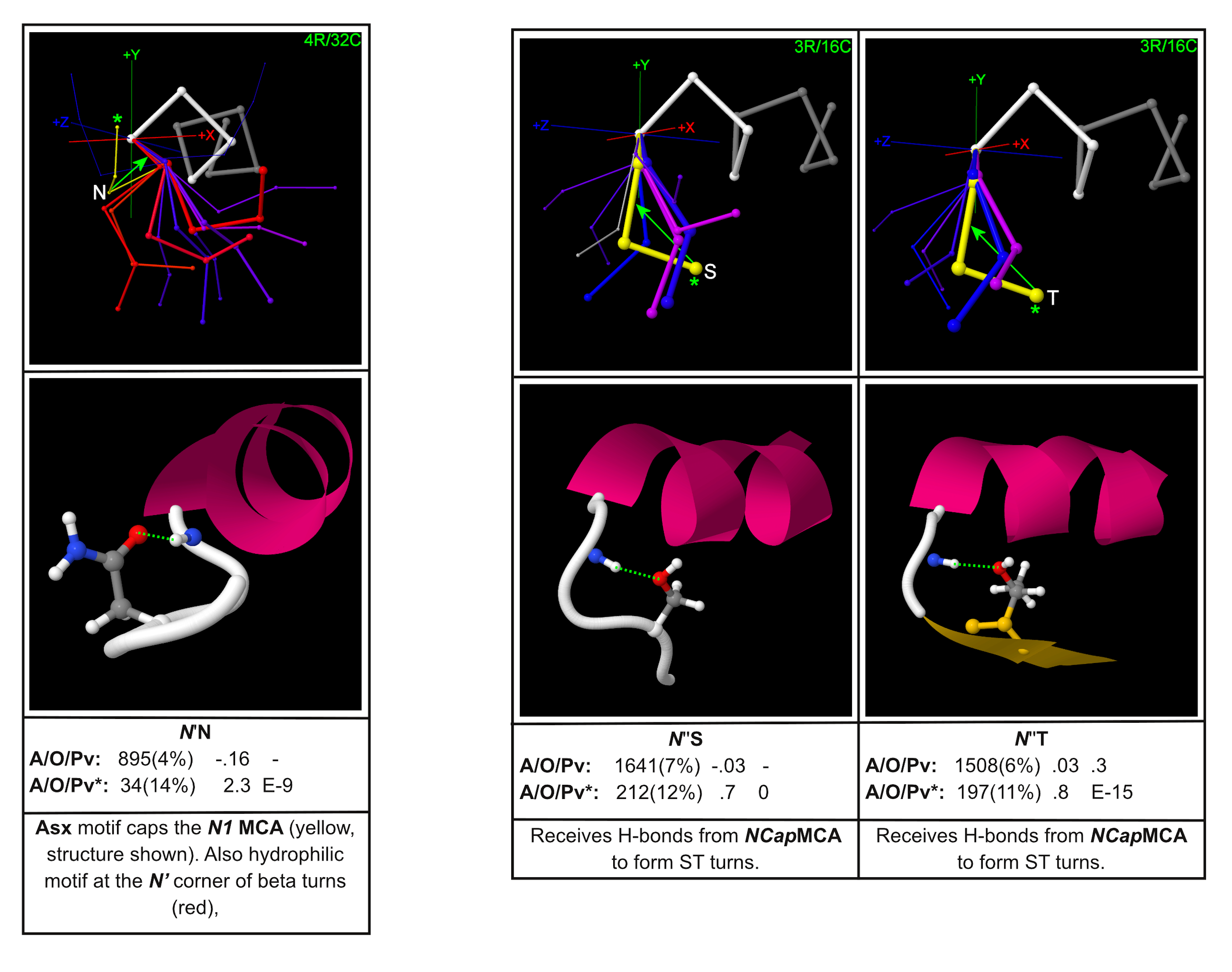

Supplement: Additional file 3: — Asx and ST motifs at N’ and N”. Asx and ST motifs that occur at N’ and N” are mapped, with example structures and global and peak-cluster (*) motif data (Abundance/Overrepresentation/Pvalue). Exemplar width is proportional to motif abundance in the corresponding cluster/geometry, while exemplar colour is proportional to overrepresentation. Asx and ST motifs show characteristic geometries at N’ and N”, as they do at NCap and N”’. [file 12859_2015_671_MOESM3_ESM.tiff]

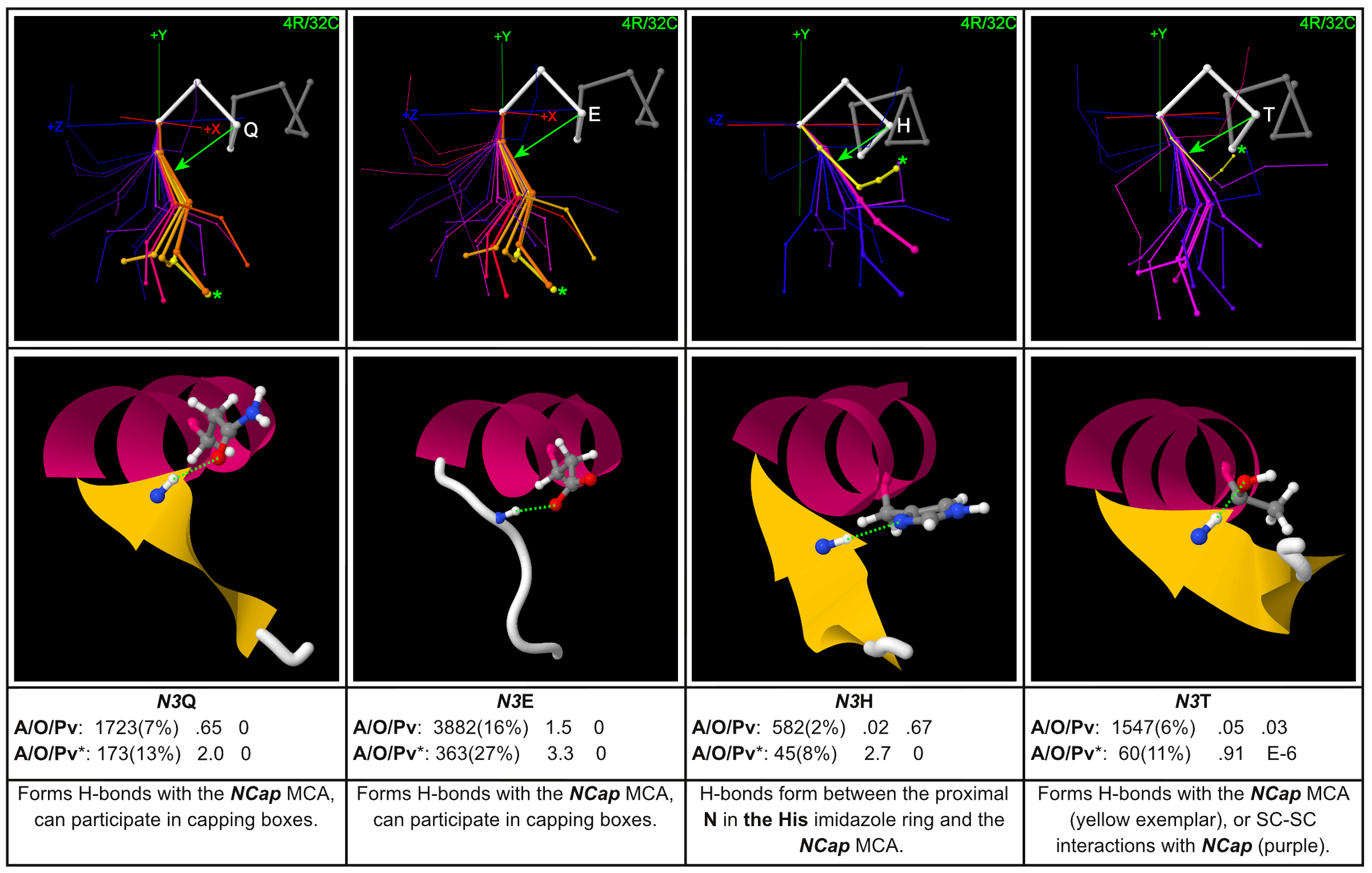

Supplement: Additional file 4: — First-order polar motifs at N3 . First-order polar motifs at N3, with example structures and global and peak-cluster (*) motif data (Abundance/Overrepresentation/Pvalue). Exemplar width is proportional to motif abundance in the corresponding cluster/geometry, while exemplar colour is proportional to overrepresentation. Polar amino acids at N3 are well-positioned to interact with the loop, and they play important roles which include serving as components of capping boxes and big boxes. [file 12859_2015_671_MOESM4_ESM.tiff]

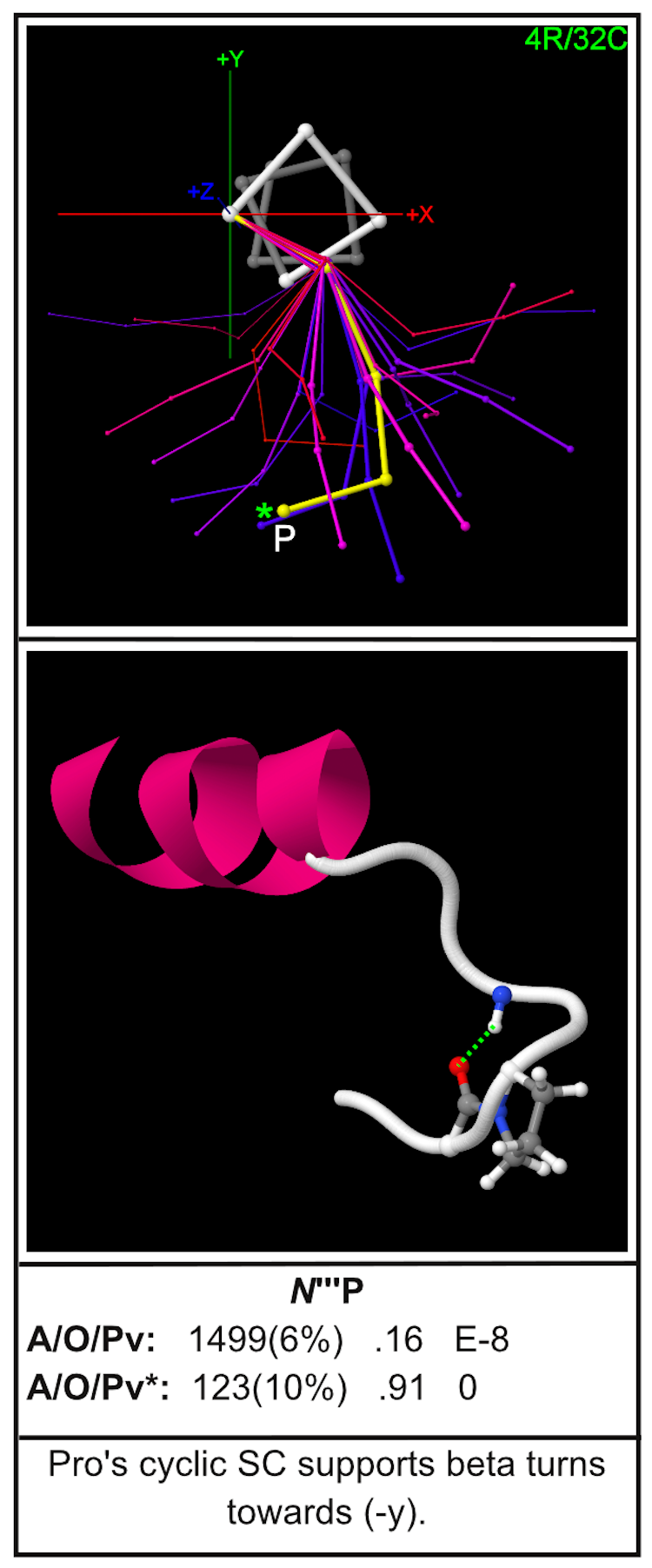

Supplement: Additional file 5: — First-order proline motif at N”’. The first-order proline motif at N”’ is mapped, with example structure and global and peak-cluster (*) motif data (Abundance/Overrepresentation/Pvalue). Exemplar width is proportional to motif abundance in the corresponding cluster/geometry, while exemplar colour is proportional to overrepresentation. This motif favours a loop geometry that brings the backbone towards (−y) and supports a beta-turn towards (-x, -y). [file 12859_2015_671_MOESM5_ESM.tiff]

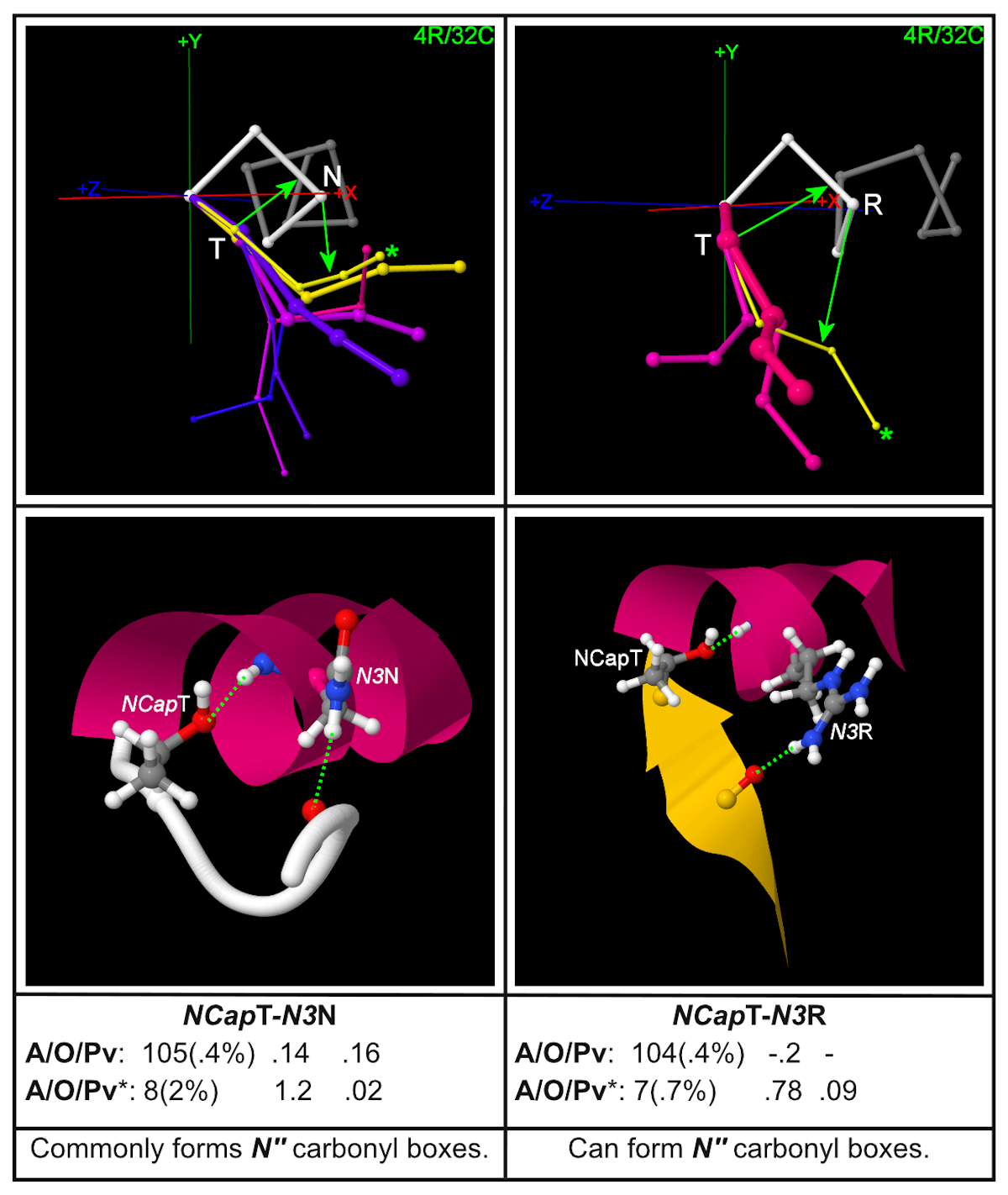

Supplement: Additional file 6: — N” carbonyl boxes. The pair motifs NCapT-N3N and NCapT-N3R are mapped, with example structures and global and peak-cluster (*) motif data (Abundance/Overrepresentation/Pvalue). Exemplar width is proportional to motif abundance in the corresponding cluster/geometry, while exemplar colour is proportional to overrepresentation. These motifs can form “N” carbonyl boxes”, in which the ST N-cap motif is joined by an H-bond between the N3SC and the N”MCC. [file 12859_2015_671_MOESM6_ESM.tif]

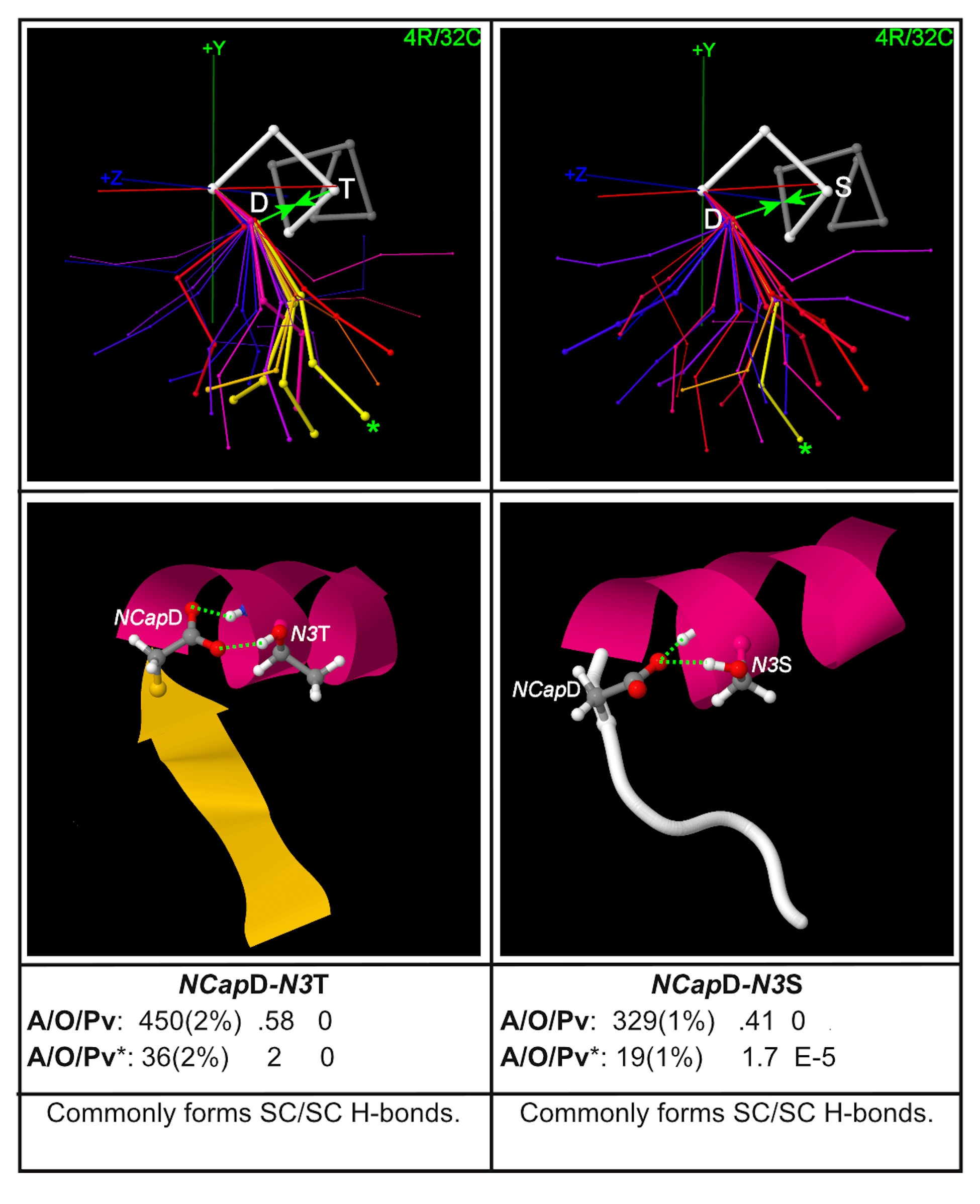

Supplement: Additional file 7: — Capping box alternatives. Examples of motifs that have polar residues at NCap and N3 but tend to form structures other than the capping box are mapped, with example structures and global and peak-cluster (*) motif data (Abundance/Overrepresentation/Pvalue). Exemplar width is proportional to motif abundance in the corresponding cluster/geometry, while exemplar colour is proportional to overrepresentation. The motifs NCapD-N3T and NCapD-N3S commonly form SC/SC H-bonds rather than the reciprocal SC/MC H-bonds of the capping box. [file 12859_2015_671_MOESM7_ESM.tiff]

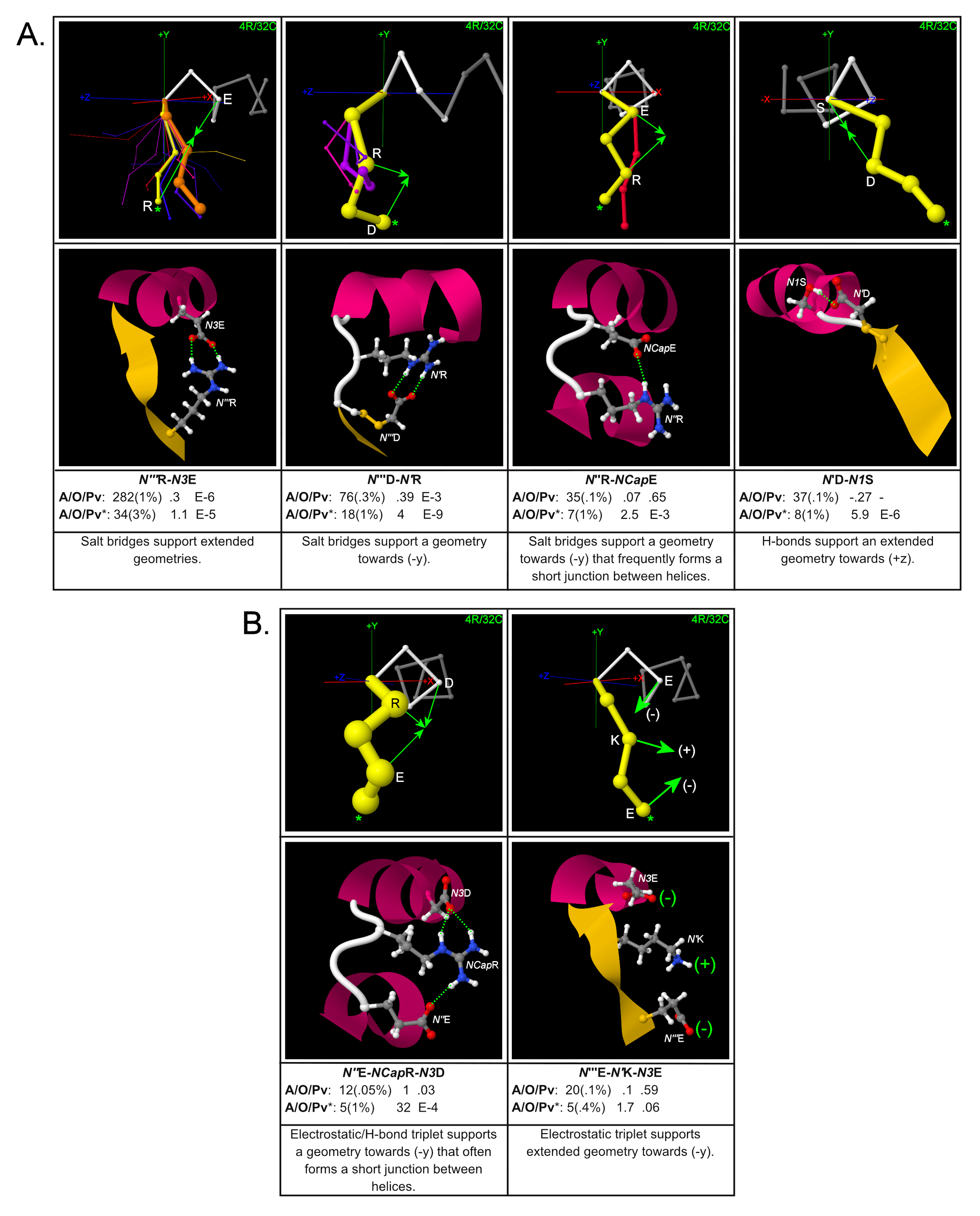

Supplement: Additional file 8: — Miscellaneous polar SC/SC motifs at the N-terminus. A selection of pair (A) and triplet (B) polar motifs at the N-terminus is shown, with example structures and global and peak-cluster (*) motif data (Abundance/Overrepresentation/Pvalue). Exemplar width is proportional to motif abundance in the corresponding cluster/geometry, while exemplar colour is proportional to overrepresentation. These motifs are associated with salt bridges, H-bonds and electrostatic interactions. [file 12859_2015_671_MOESM8_ESM.tif]

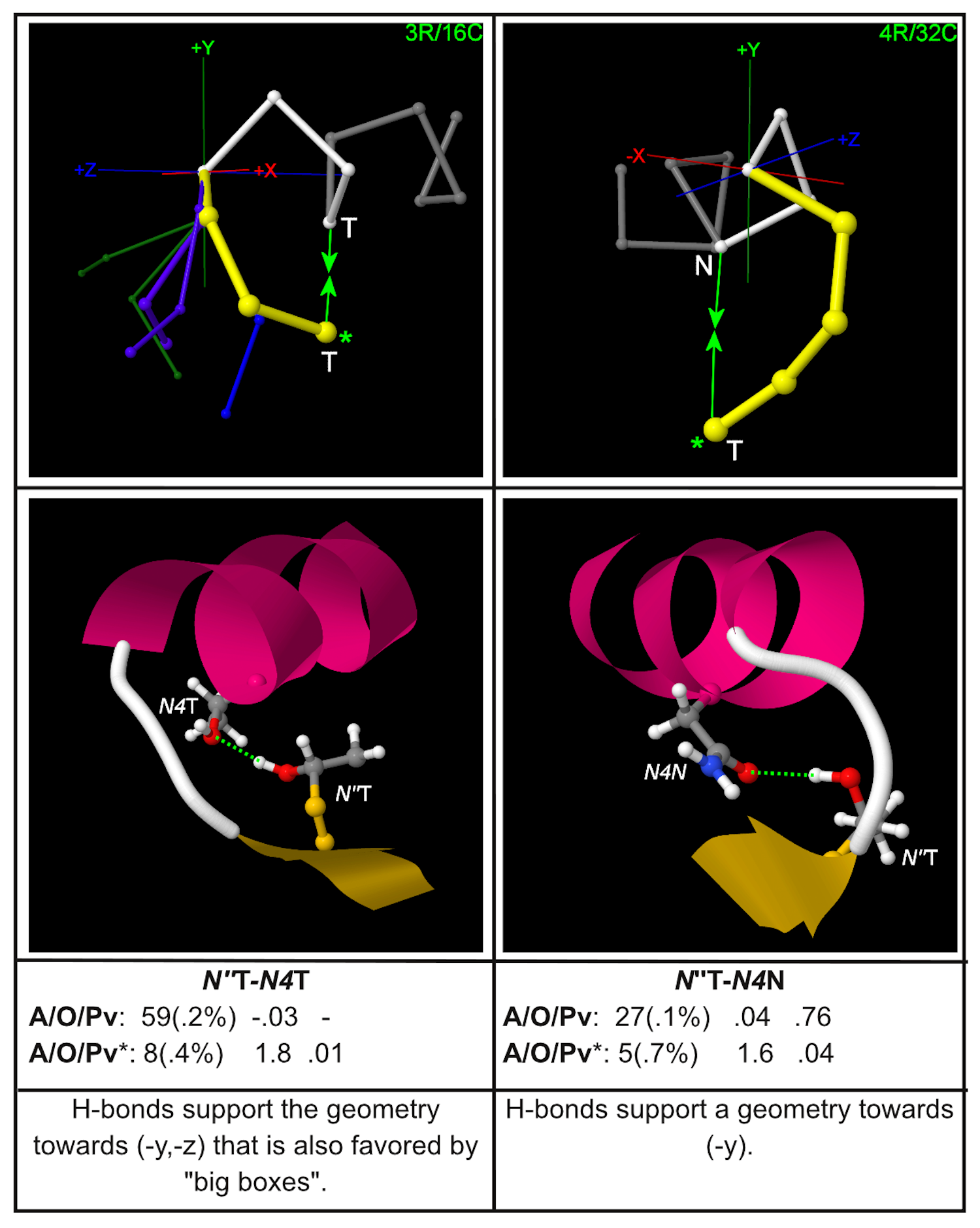

Supplement: Additional file 9: — Polar pairs at ( N” , N4 ). The polar pair motifs N”T-N4T and N”T-N4N are mapped, with example structures and global and peak-cluster (*) motif data (Abundance/Overrepresentation/Pvalue). Exemplar width is proportional to motif abundance in the corresponding cluster/geometry, while exemplar colour is proportional to overrepresentation. These motifs support particular geometries with SC/SC H-bonds. [file 12859_2015_671_MOESM9_ESM.tif]

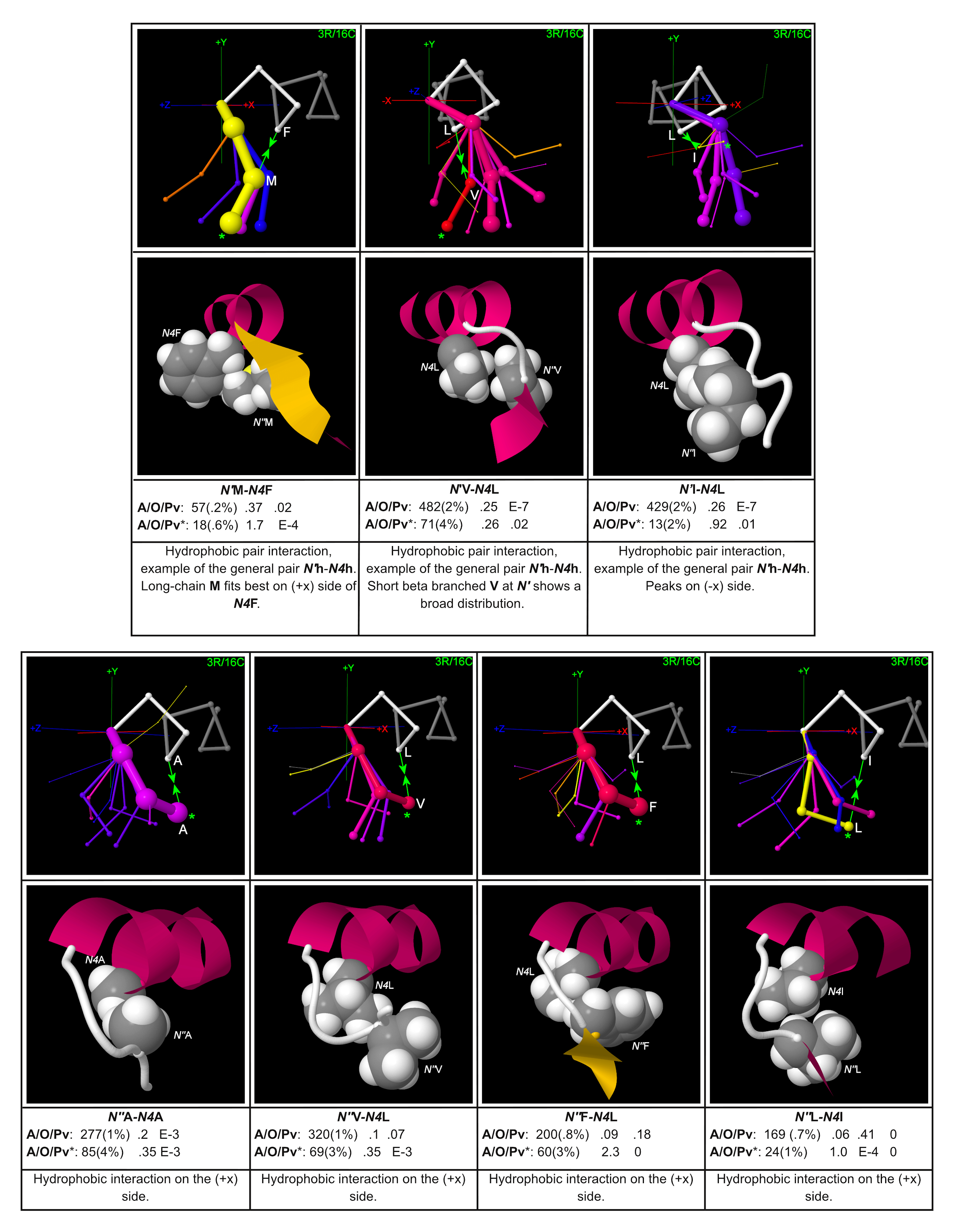

Supplement: Additional file 10: — Particular hydrophobic pair motifs at the N-terminus. Particular instances of hydrophobic pair motifs, at (N’, N4) (A) and (N”, N4) (B) are shown, with example structures and global and peak-cluster (*) motif data (Abundance/Overrepresentation/Pvalue). Exemplar width is proportional to motif abundance in the corresponding cluster/geometry, while exemplar colour is proportional to overrepresentation. These motifs include particular examples of the general hydrophobic pairs from Fig. 8. Motifs occurring at the position pair (N”, N4) show prominent peaks in the geometries in which the loop turns towards (−z) at N’, bringing the SCs at N” and N4 close for hydrophobic interaction. [file 12859_2015_671_MOESM10_ESM.tiff]

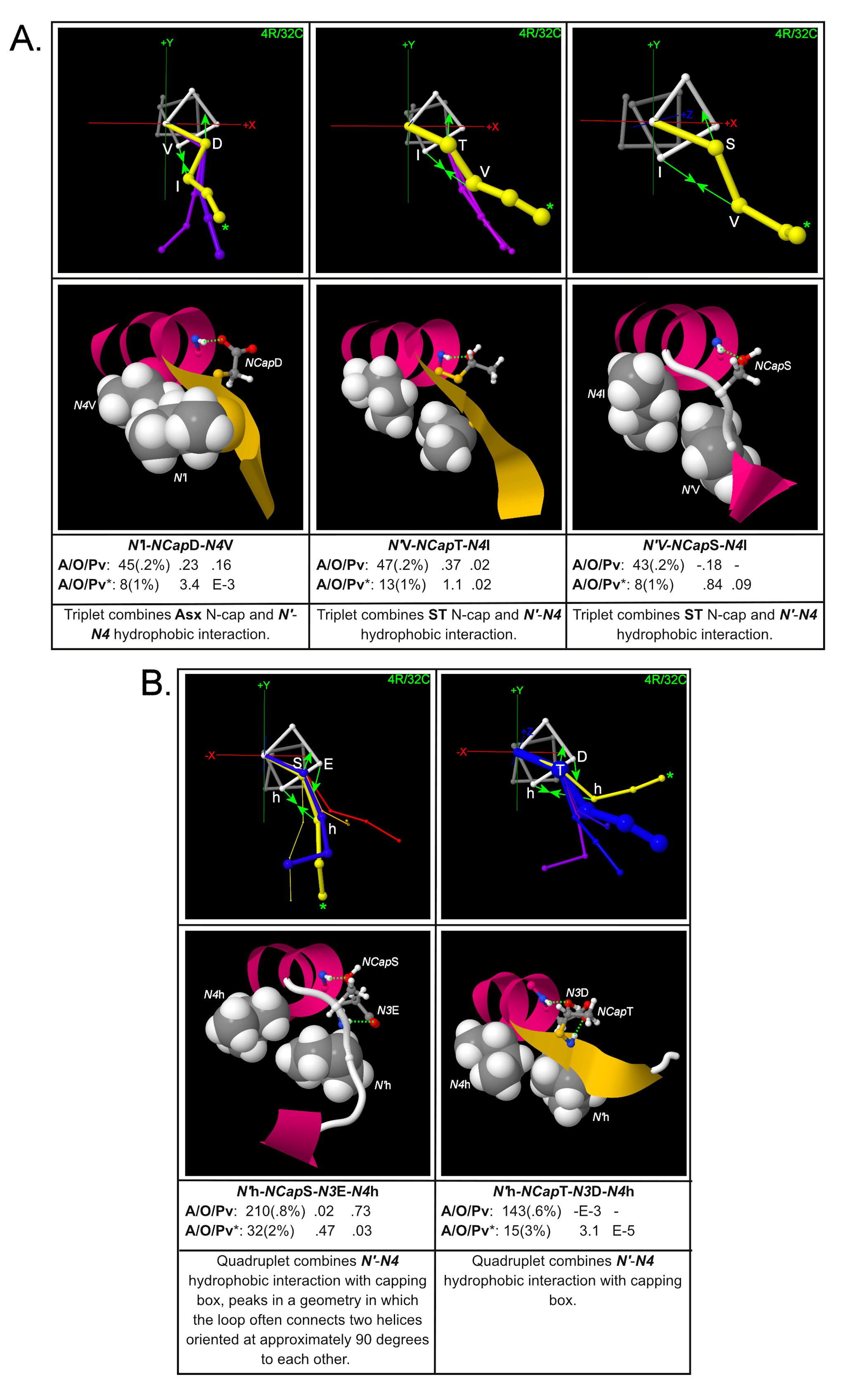

Supplement: Additional file 11: — Higher-order hydrophobic motifs at the N-terminus. Triplet (A) and quadruplet (B) motifs are mapped, with example structures and global and peak-cluster (*) motif data (Abundance/Overrepresentation/Pvalue). Exemplar width is proportional to motif abundance in the corresponding cluster/geometry, while exemplar colour is proportional to overrepresentation. Three triplet motifs that combine Asx/ST N-caps with particular pairs of hydrophobic amino acids at (N’, N4) are mapped, along with two quadruplet motifs that combine capping boxes with general hydrophobic pairs. [file 12859_2015_671_MOESM11_ESM.tif]

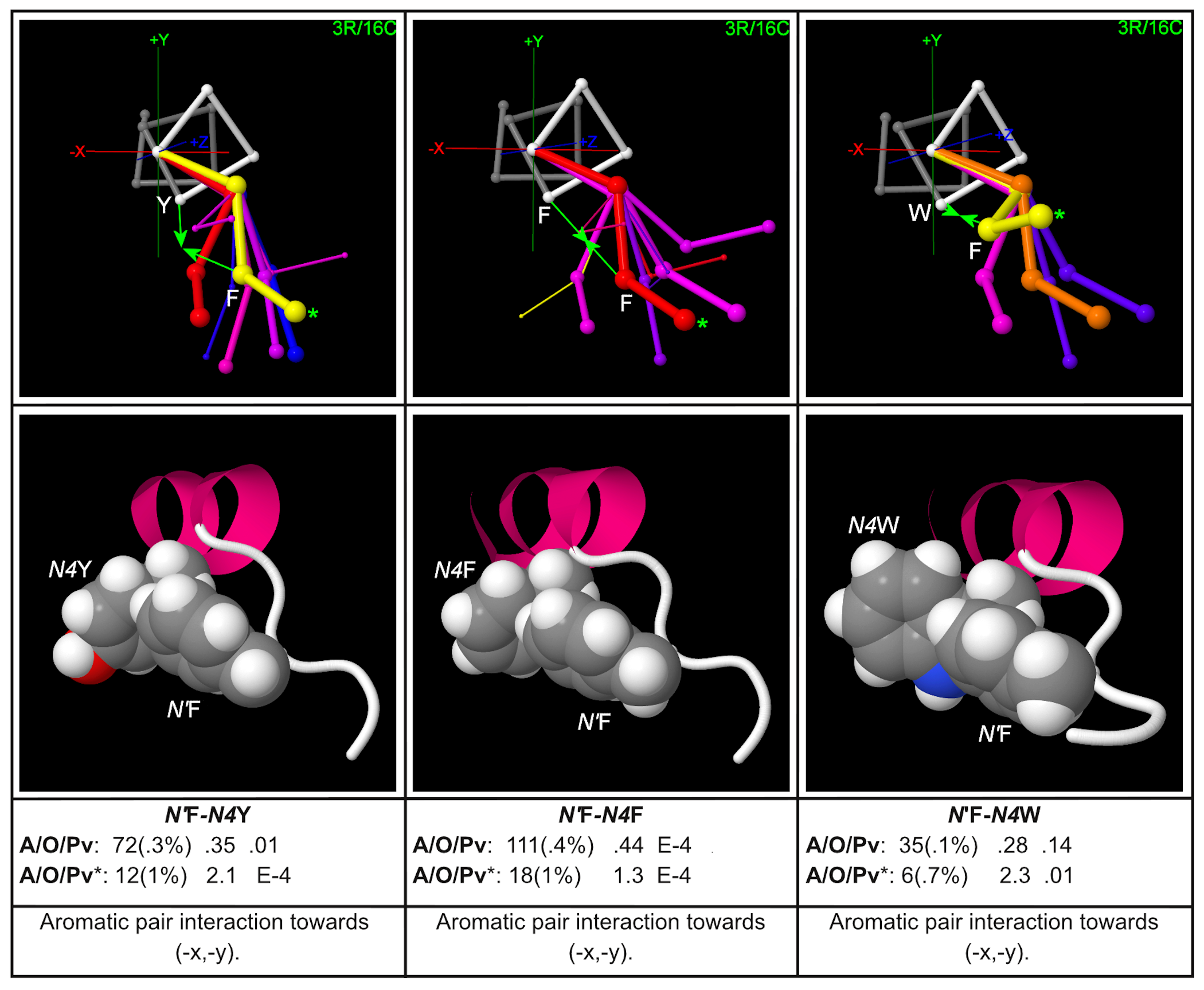

Supplement: Additional file 12: — Aromatic pair motifs at the N-terminus. Three aromatic pair motifs at the N-terminus are mapped, with example structures and global and peak-cluster (*) motif data (Abundance/Overrepresentation/Pvalue). Exemplar width is proportional to motif abundance in the corresponding cluster/geometry, while exemplar colour is proportional to overrepresentation. All three motifs can form perpendicular pi stacking interactions on the (−x) side. [file 12859_2015_671_MOESM12_ESM.tif]

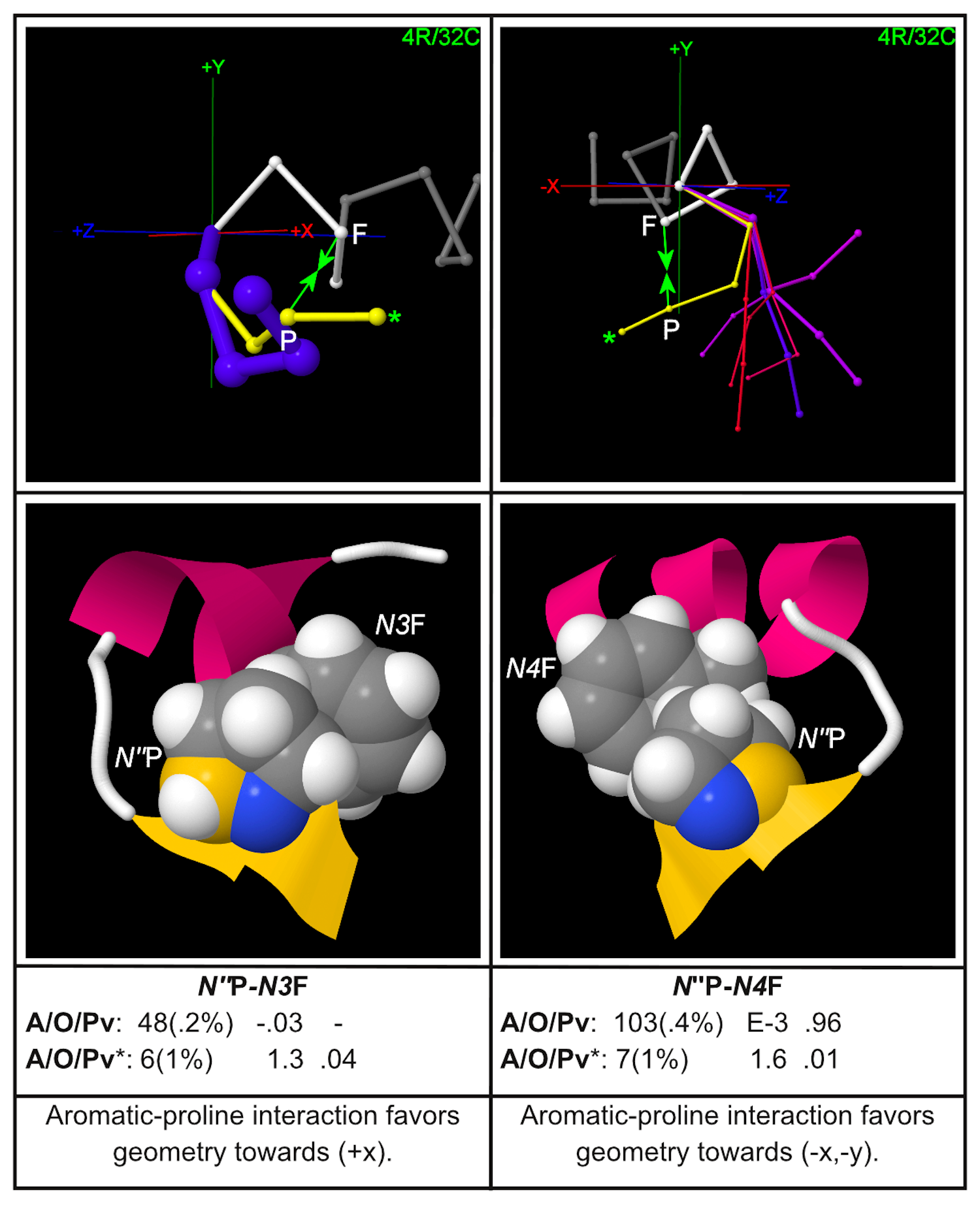

Supplement: Additional file 13: — Aromatic-proline pairs at the N-terminus. Two pair motifs that represent aromatic-proline interactions at the N-terminus are mapped, with example structures and global and peak-cluster (*) motif data (Abundance/Overrepresentation/Pvalue). Exemplar width is proportional to motif abundance in the corresponding cluster/geometry, while exemplar colour is proportional to overrepresentation. [file 12859_2015_671_MOESM13_ESM.tif]

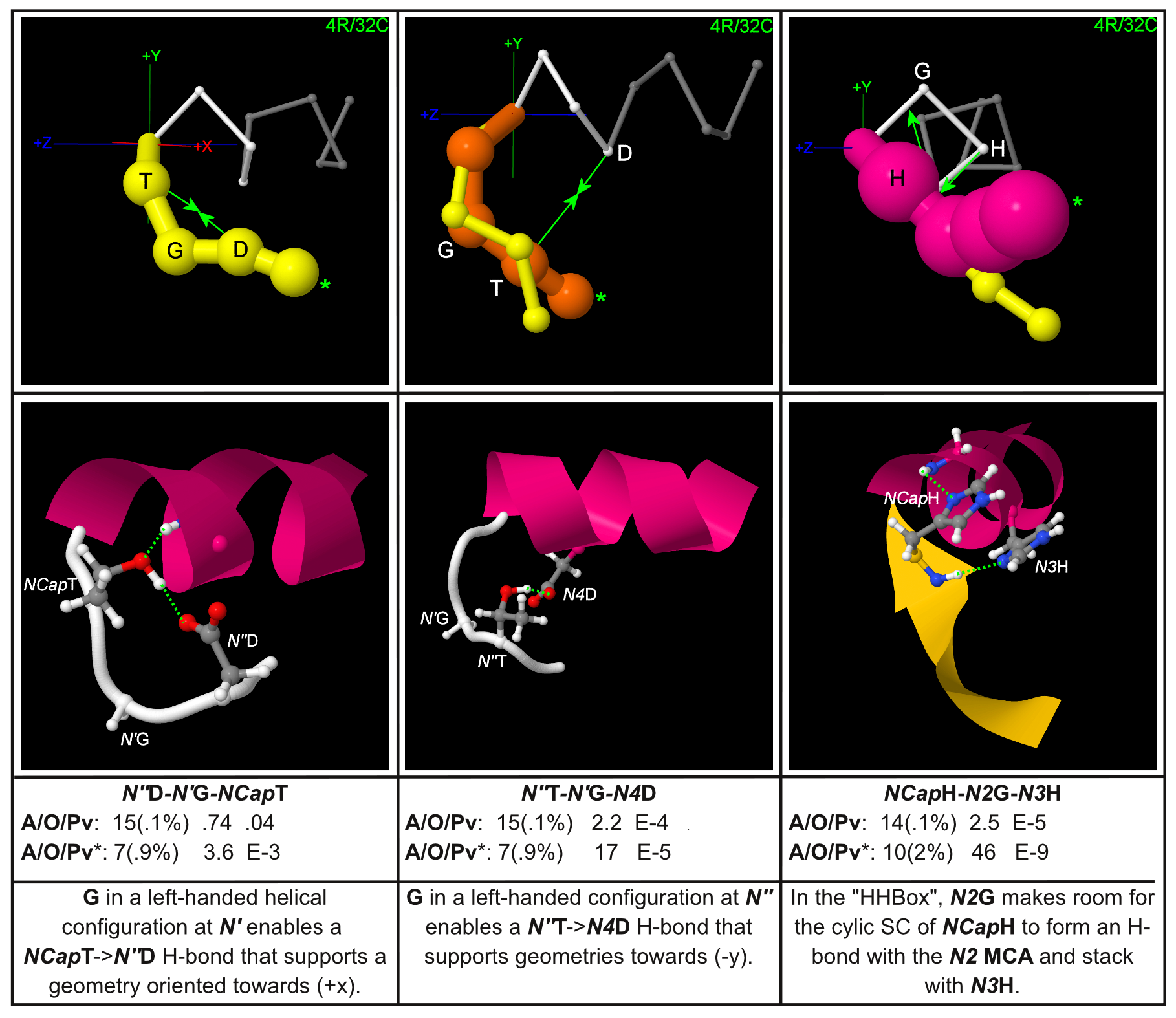

Supplement: Additional file 14: — Triplet motifs with glycine at the N-terminus. Three triplet motifs that incorporate glycine at the N-terminus are mapped, with example structures and global and peak-cluster (*) motif data (Abundance/Overrepresentation/Pvalue). Exemplar width is proportional to motif abundance in the corresponding cluster/geometry, while exemplar colour is proportional to overrepresentation. In these motifs, glycine contributes conformational flexibility or space for packing. [file 12859_2015_671_MOESM14_ESM.tif]

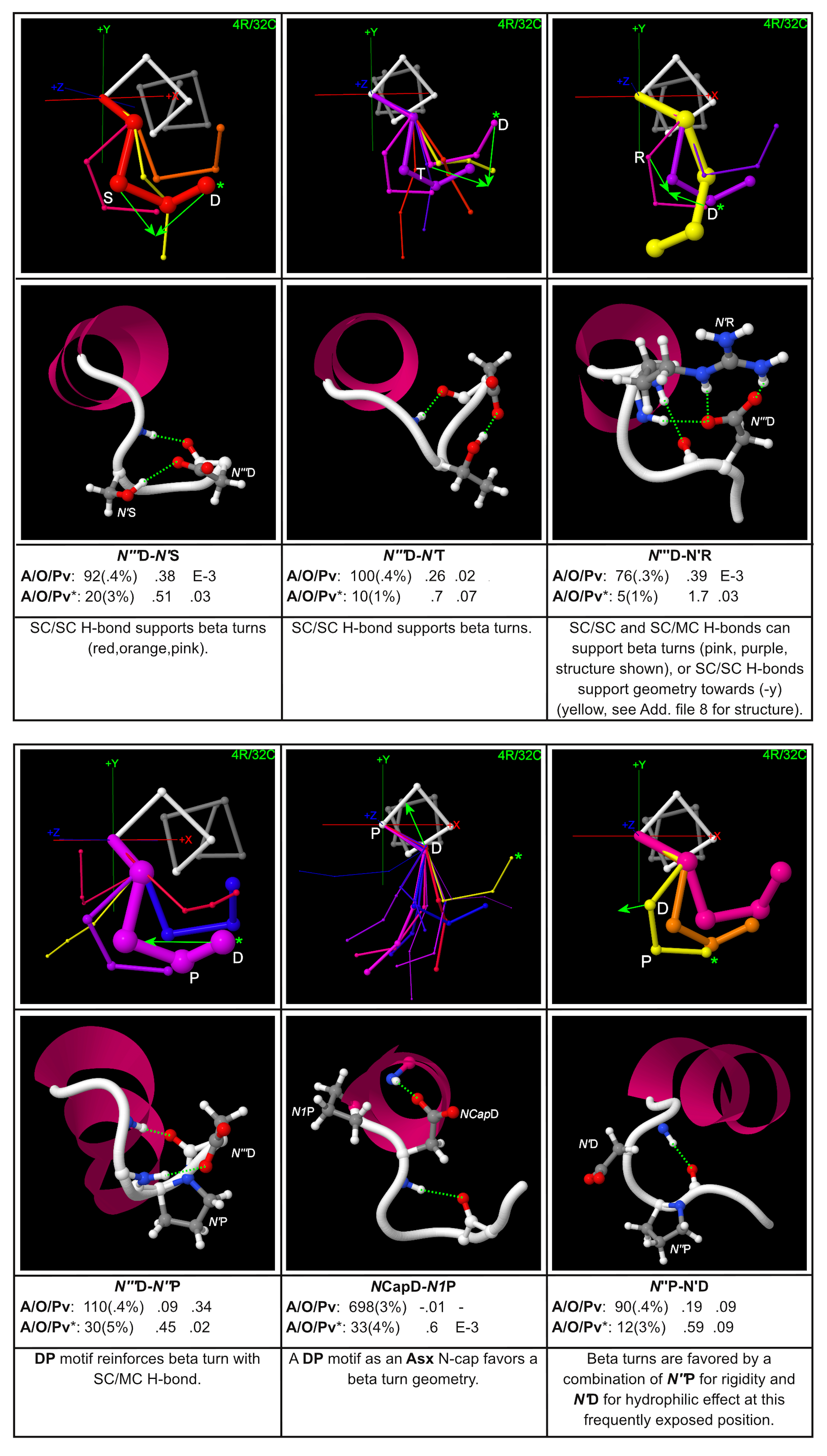

Supplement: Additional file 15: — Beta-turn motifs at the N-terminus. Six pair motifs that favour beta-turn geometries are mapped, with example structures and global and peak-cluster (*) motif data (Abundance/Overrepresentation/Pvalue). Exemplar width is proportional to motif abundance in the corresponding cluster/geometry, while exemplar colour is proportional to overrepresentation. [file 12859_2015_671_MOESM15_ESM.tiff]

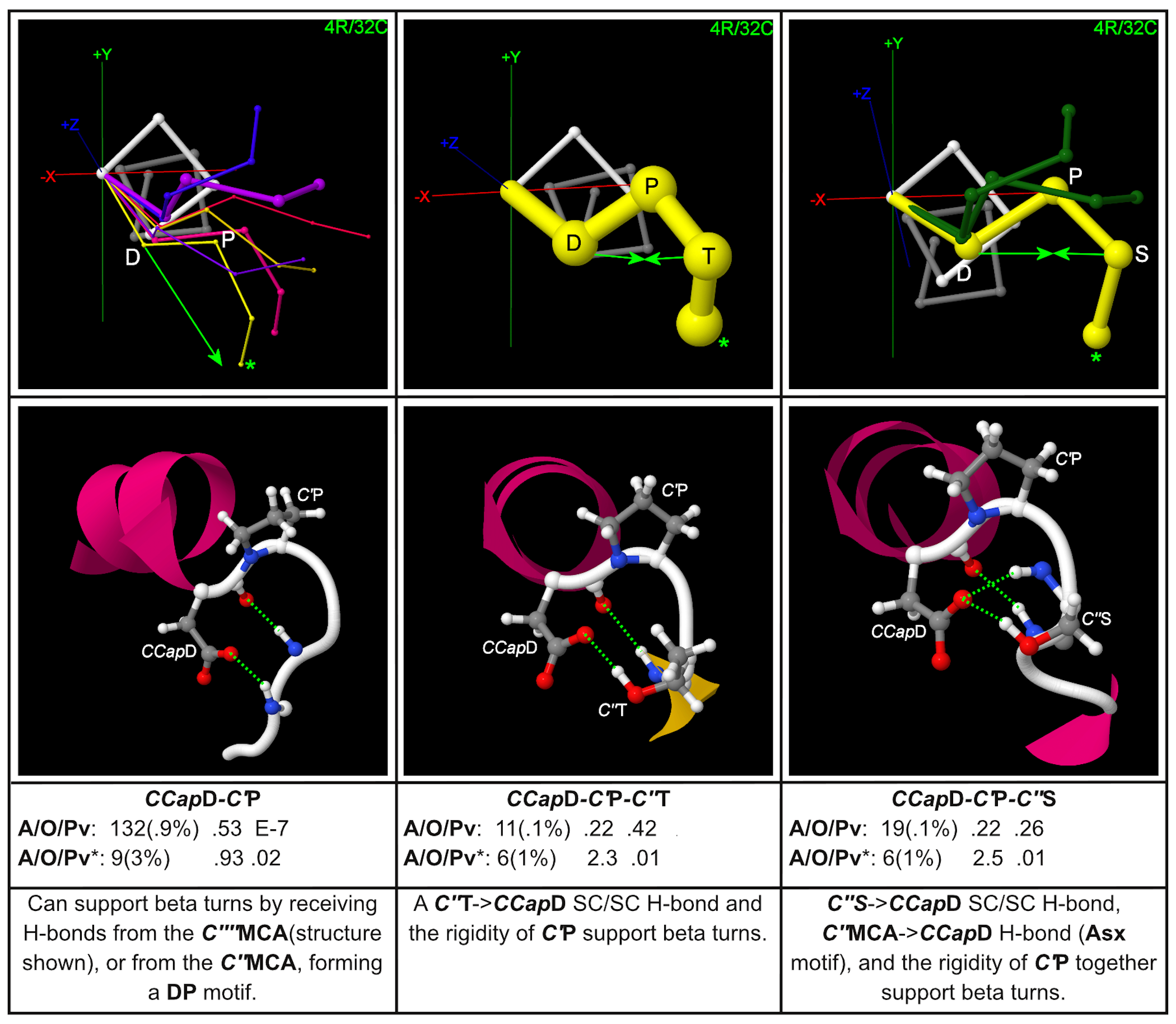

Supplement: Additional file 16: — Proline motifs at the C-terminus. A pair and two triplet motifs that include proline are mapped, with example structures and global and peak-cluster (*) motif data (Abundance/Overrepresentation/Pvalue). Exemplar width is proportional to motif abundance in the corresponding cluster/geometry, while exemplar colour is proportional to overrepresentation. Asp and Pro can form DP motifs near the C-terminus, and Ser or Thr at C”’ can join to form triplet motifs. [file 12859_2015_671_MOESM16_ESM.tif]

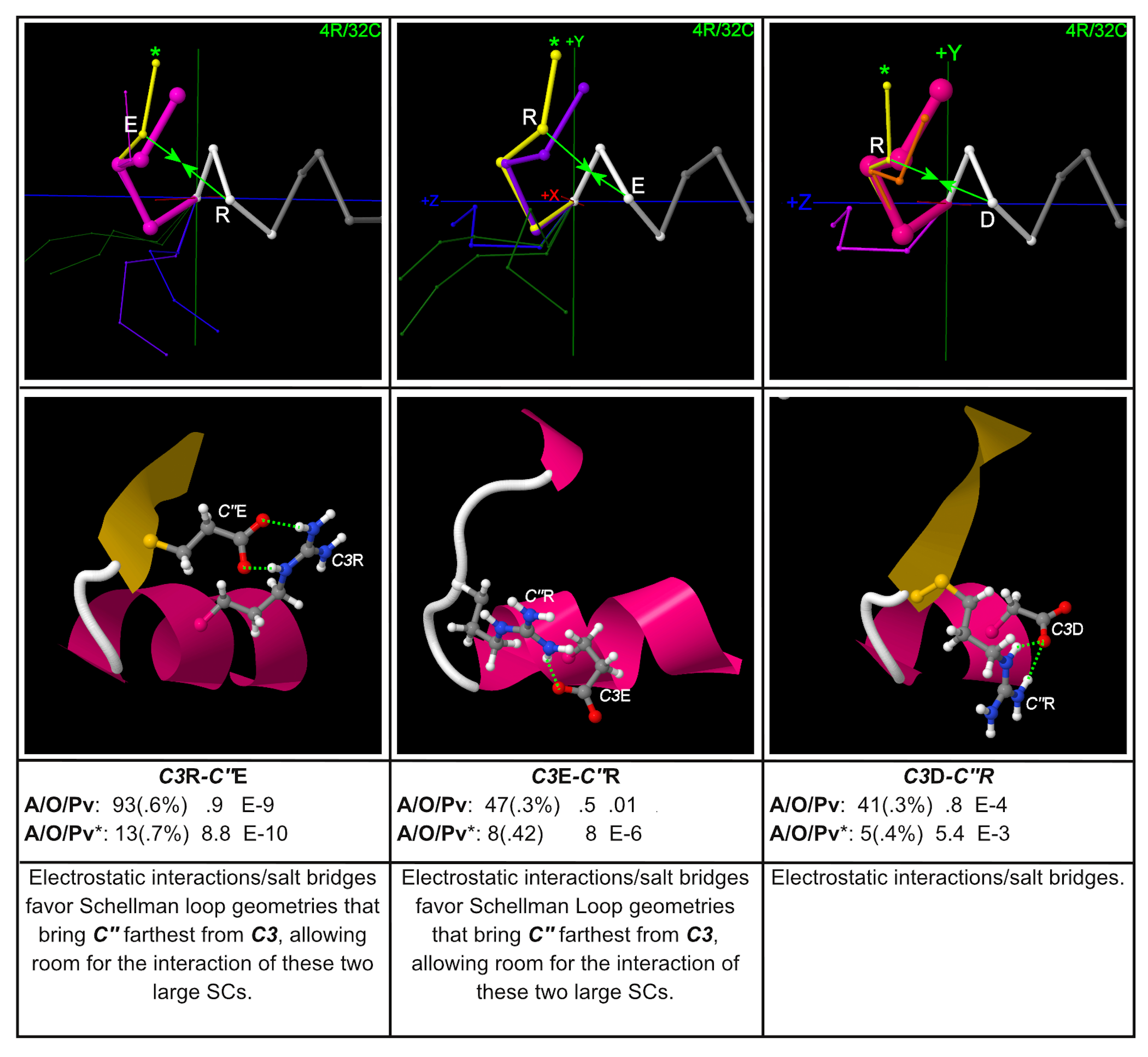

Supplement: Additional file 17: — Polar pairs at ( C3 , C” ). Three polar pairs at (C3, C”) are mapped, with example structures and global and peak-cluster (*) motif data (Abundance/Overrepresentation/Pvalue). Exemplar width is proportional to motif abundance in the corresponding cluster/geometry, while exemplar colour is proportional to overrepresentation. These motifs exhibit electrostatic interactions and salt bridges that favour Schellman loops. [file 12859_2015_671_MOESM17_ESM.tif]

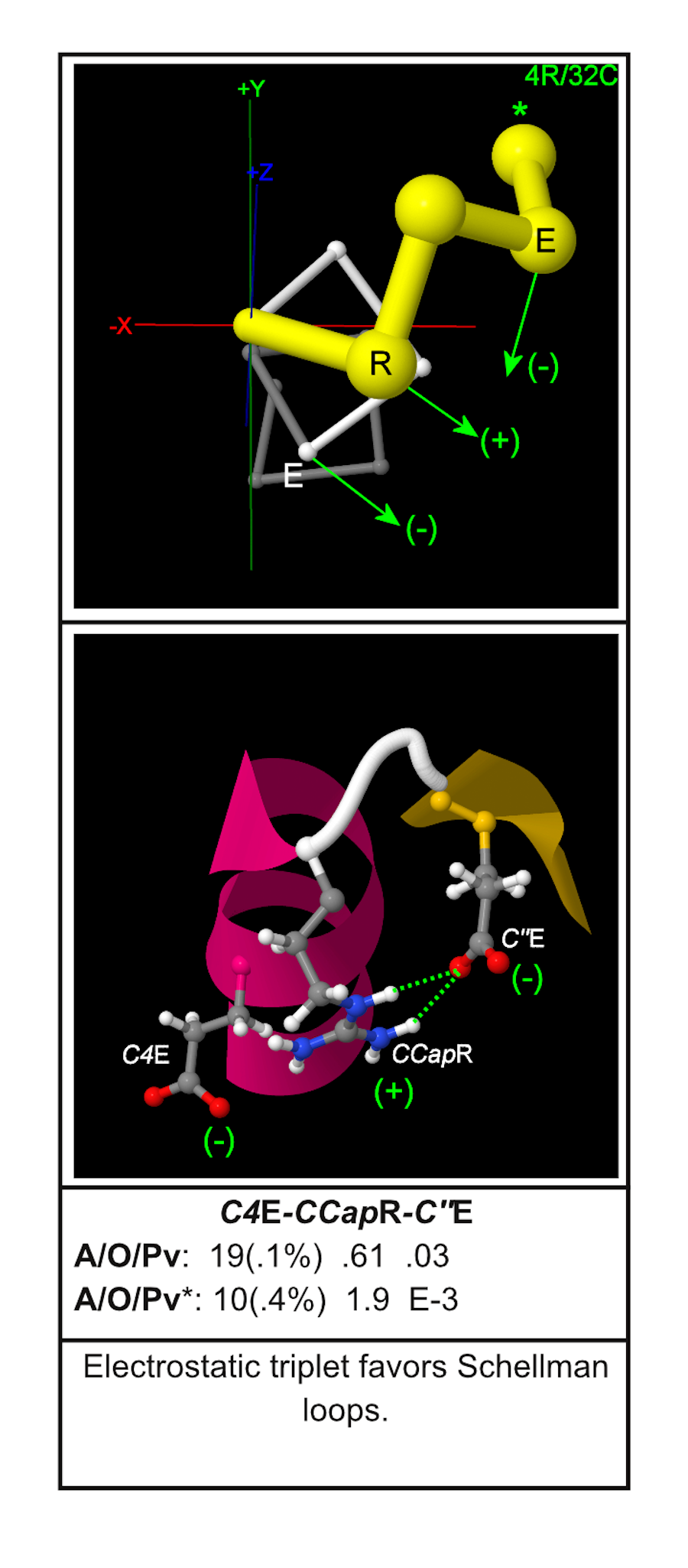

Supplement: Additional file 18: — Electrostatic triplet at the C-terminus. The electrostatic triplet C4E-CCapR-C”E is mapped, with an example structure and global and peak-cluster (*) motif data (Abundance/Overrepresentation/Pvalue). Exemplar width is proportional to motif abundance in the corresponding cluster/geometry, while exemplar colour is proportional to overrepresentation. This motif favours a Schellman loop geometry. [file 12859_2015_671_MOESM18_ESM.tif]

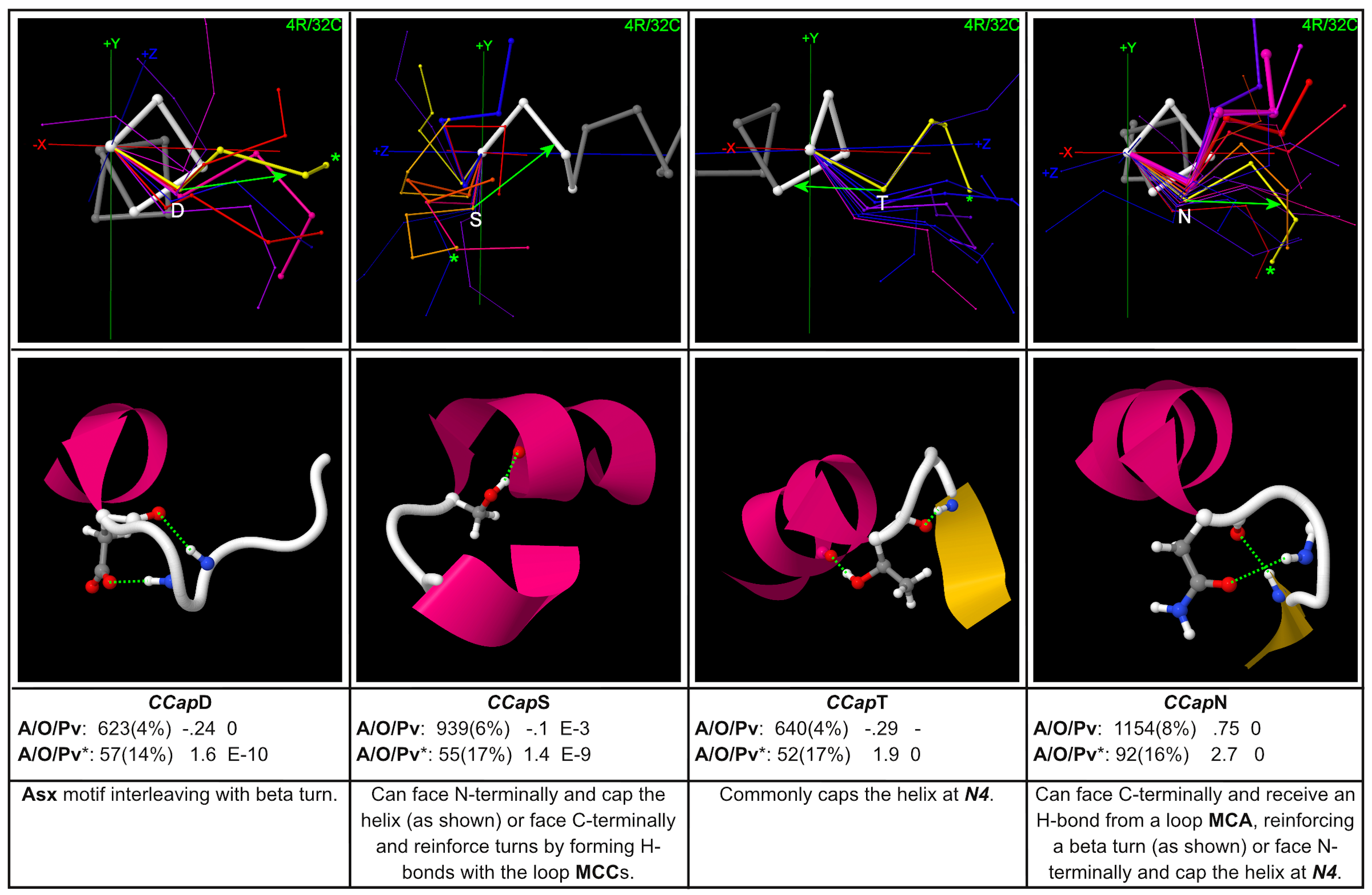

Supplement: Additional file 19: — First-order polar motifs with {Asp, Asn, Ser, Thr} at CCap . First-order polar motifs at CCap are mapped, with example structures and global and peak-cluster (*) motif data (Abundance/Overrepresentation/Pvalue). Exemplar width is proportional to motif abundance in the corresponding cluster/geometry, while exemplar colour is proportional to overrepresentation. Although the polar amino acids {Asp, Asn, Ser, Thr} are much less important at CCap than they are at NCap, they nevertheless play structural roles by capping the helix or interacting with the loop. [file 12859_2015_671_MOESM19_ESM.tiff]

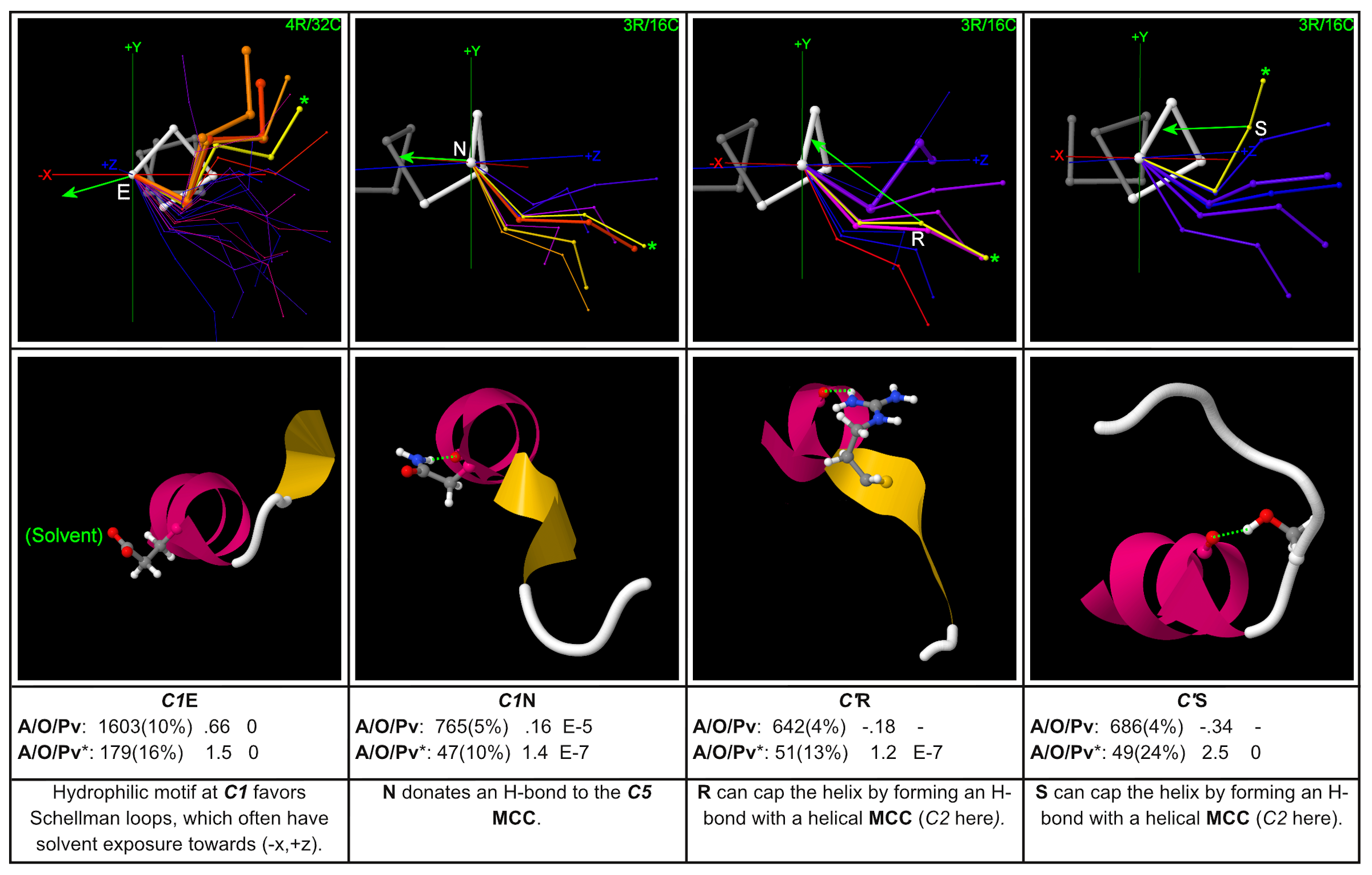

Supplement: Additional file 20: — First-order polar motifs at C1 and C’ . First-order polar motifs at C1 and C’ are mapped, with example structures and global and peak-cluster (*) motif data (Abundance/Overrepresentation/Pvalue). Exemplar width is proportional to motif abundance in the corresponding cluster/geometry, while exemplar colour is proportional to overrepresentation. Polar motifs at these positions may play a hydrophilic role (C1E), or cap the helix {C1N, C’R, C’S}. [file 12859_2015_671_MOESM20_ESM.tif]

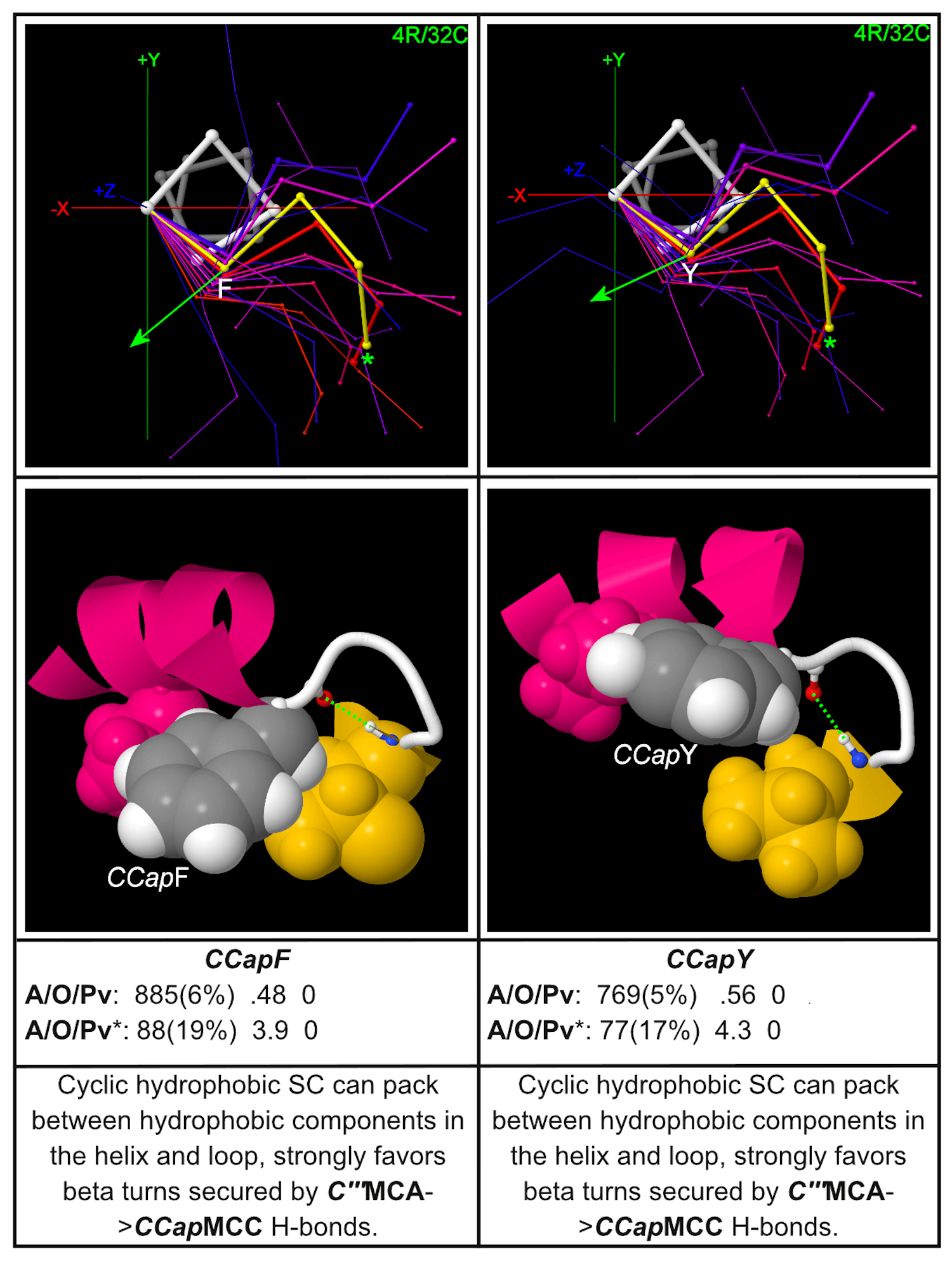

Supplement: Additional file 21: — First-order aromatic motifs at CCap . The first-order aromatic motifs CCapF and CCapY are mapped, with example structures and global and peak-cluster (*) motif data (Abundance/Overrepresentation/Pvalue). Exemplar width is proportional to motif abundance in the corresponding cluster/geometry, while exemplar colour is proportional to overrepresentation. The cyclic SCs of these motifs can pack between hydrophobic components in the helix and loop, supporting beta-turns secured by C”’MCA → CCapMCC H-bonds. [file 12859_2015_671_MOESM21_ESM.tiff]
